# Supplementary material for: Design and Synthesis of Conformationally Flexible Scaffold as Bitopic Ligands for Potent D3-Selective Antagonists
Source: Int J Mol Sci. 2022 Dec 27;24(1):432. doi: 10.3390/ijms24010432 (PMC9820167; doi:10.3390/ijms24010432)
Supplement: Supplementary file 1 [file ijms-24-00432-s001.zip › ijms-2107160-supplementary.pdf]

Supplementary materials

Index

1. Supplemental Table 1……………………………………………………………………S3

2. Supplemental Table 2……………………………………………………………………S4

3. Supplemental Table 3..……………………………….…………………………………S6

4. Analyses of HRMS…………………………….…………………………………………S8

5. ^{1}H or ^{13}C NMR spectra ………………………………………………………………S21

Supplemental Table 1. UPLC purity of investigated compounds

Condition: Mobile phase: A: 0.1% Formic acid, B: Acetonitrile.

Stationary phase: waters acquity UPLC® HSS 1.8 µm C18 (2.1 × 50 mm)

(1) 95% to 1% of A in B for 3.3 min, 1% of A in B for 0.55 min, 1% to 95% of A in B for 0.05 min, and 95% of A in B for 0.1 min. Flow rate: 0.75 mL/ min for **6a-d**, **9b**, **11a-b**, **12**, **20a-c, 21d**, and **21g-h**.

(2) 95% of A in B for 0.5 min, 95% to 10% of A in B for 2 min, 10% of A in B for 0.5 min, 10% to 95% of A in B for 0.1 min, and 95% of A in B for 0.4 min. Flow rate: 0.5 mL/ min for **9a**, **14**, **21a-c**, **21e-f**, and **21i-j**.

Name	Purity (%)	Name	Purity (%)	Name	Purity (%)
6a	99.0%	12	95.4%	21d	97.2%
6b	95.8%	24	95.1%	21e	95.1%
6c	99.5%	20a	98.8%	21f	96.2%
6d	98.8%	20b	96.1%	21g	95.3%
9a	98.5%	20c	95.8%	21h	96.2%
9b	97.6%	21a	98.0%	21i	97.0%
11a	95.8%	21b	98.9%	21j	95.4%
11b	96.7%	21c	97.5%		

Supplemental Table 2. Binding profiles of potential candidates for other GPCRs[a]

GPCRs		*K*i (nM)[b] or % inhibition at 10 µM[c]								
		9a	20a	21a	21c	21d	21e	21g	21i	21j
Serotonin	5-HT$_{1A}$	1741	194	971	509	692	575	3312	245	961
	5-HT$_{1B}$	905	1052	N/A[d]	N/A	N/A	N/A	1056	N/A	N/A
	5-HT$_{1D}$	1006	449	1204	1150	1655	N/A	1825	3137	1932
	5-HT$_{1E}$	N/A	N/A	N/A	N/A	N/A	N/A	N/A	N/A	N/A
	5-HT$_{2A}$	222	617	1244	727	844	1328	276	1368	3141
	5-HT$_{2B}$	187	170	118	157	181	443	189	269	183
	5-HT$_{2C}$	577	2021	1326	1496	2105	5782	354	9727	5057
	5-HT$_3$	195	103	53	58	92	148	195	29	246
	5-HT$_{5A}$	N/A	N/A	N/A	N/A	N/A	N/A	N/A	N/A	N/A
	5-HT$_6$	287	258	730	1857	1626	2333	363	2157	1314
	5-HT$_{7A}$	N/A	N/A	N/A	N/A	N/A	N/A	2247	N/A	N/A
Adrenergic	alpha$_{1A}$	N/A	2209	N/A	N/A	N/A	N/A	2114	N/A	N/A
	alpha$_{1B}$	2190	1400	3938	N/A	>10,000	N/A	1635	N/A	N/A
	alpha$_{1D}$	2556	2416	N/A	N/A	7311	N/A	1407	N/A	N/A
	alpha$_{2A}$	1346	740	1967	2584	2141	5573	1161	3336	3114
	alpha$_{2B}$	235	672	577	909	386	2835	571	2949	646
	alpha$_{2C}$	313	113	195	524	532	1761	78	1291	183
	beta$_1$	5897	3713	N/A	N/A	N/A	6518	1891	N/A	N/A
	beta$_2$	669	5834	N/A	N/A	N/A	N/A	616	N/A	>10,000
	beta$_3$	1496	1386	4527	N/A	N/A	5565	2958	>10,000	4359
Dopamine	D_1	2402	4292	N/A	N/A	N/A	N/A	2007	N/A	N/A
	D_2	232	185	216	74	89	N/A	170	40	76
	D_3	0.83	1.32	0.19	0.36	0.25	0.46	0.61	0.28	0.34
	D_4	579	938	377	2183	239	N/A	582	514	878
	D_5	1467	N/A	N/A	>10,000	N/A	N/A	8692	N/A	N/A
Opioid	MOR	1556	3020	1076	N/A	2405	N/A	2156	N/A	2771

	KOR	107	116	N/A	642	2326	N/A	170	N/A	N/A
	DOR	N/A	N/A	N/A	N/A	N/A	N/A	N/A	N/A	N/A
Histamine	H_1	1492	1357	1508	591	0.95	675	102	84	1465
	H_2	2614	1650	2793	N/A	N/A	N/A	2478	N/A	3683
	H_3	5580	N/A	N/A	4725	>10,000	N/A	4812	N/A	N/A
	H_4	6425	3462	N/A	N/A	N/A	N/A	N/A	N/A	N/A
Muscarinic	M_1	2355	N/A	N/A	3392	>10,000	N/A	3432	N/A	N/A
	M_2	1205	4931	N/A	1657	N/A	9441	N/A	N/A	2968
	M_3	4750	N/A	1264	N/A	N/A	1547	N/A	N/A	N/A
	M_4	1602	N/A	N/A	930	N/A	N/A	1094	N/A	N/A
	M_5	5038	N/A	N/A	2388	4223	N/A	N/A	N/A	N/A
Sigma	$sigma_1$	473	522	241	N/A	293	N/A	694	381	204
	$sigma_2$	391	1149	1774	N/A	>10,000	N/A	1543	992	1491
BZP Rat Brain Site		N/A	N/A	N/A	N/A	N/A	N/A	N/A	N/A	N/A
$GABA_A$		N/A	N/A	N/A	N/A	N/A	N/A	N/A	N/A	N/A
Dopamine active transporter (DAT)		87	511	89	57	90	N/A	100	289	390
Norepinephrine transporter (NET)		772	3514	391	N/A	842	N/A	1940	3198	1104
Serotonin transporter (SERT)		N/A	5845	3009	N/A	N/A	N/A	N/A	N/A	415
Benzodiazepine receptor (PBR)		1454	9	9	N/A	1220	11	6	N/A	8

[a]All compounds were converted to HCl salts prior to tests. [b]*K*i values were determined by at least three experiments. [c]% of inhinition was measured using 10 μM of each compound. [d]N/A; not active means less than 50% inhibition that was obtained in primary binding assay at 10 μM of the compound.

Supplemental Table 3. List of reagents and solvents

	Name	Formula	Purity	Purchased from
Reagents	HBTU	$C_{11}H_{16}F_6N_5OP$	97%	Matrix Scientific
	DIPEA	$C_8H_{19}N$	98%	Advanced chemtech
	TFA	CF_3COOH	99%	Sigma-Aldrich
	Sodium carbonate	Na_2CO_3	≥99.5%	Sigma-Aldrich
	Dimethylamine	$(CH_3)_2NH$	2 M in THF	Sigma-Aldrich
	Sodium triacetoxyborohydride	$(CH_3COO)_3BHNa$	97%	Sigma-Aldrich
	N-(3-Boromopropyl) phthalimide	$C_{11}H_{10}BrNO_2$	98%	Sigma-Aldrich
	N-(4-Boromobutyl) phthalimide	$C_{12}H_{12}BrNO_2$	98%	Sigma-Aldrich
	Potassium carbonate	K_2CO_3	99%	Fisher chemical
	Hydrazine hydrate	$NH_2NH_2 \cdot H_2O$	50-60%	Sigma-Aldrich
	Thionyl chloride	$SOCl_2$	≥99%	Sigma-Aldrich
	4-(Thiophen-2-yl)benzoic acid	$C_{11}H_8O_2S$	97%	Sigma-Aldrich
	Triethylamine (Et_3N)	$(C_2H_5)_3N$	≥99%	Sigma-Aldrich
	Ammonia solution	NH_3	7 N in MeOH	Sigma-Aldrich
	4-methyl-5-phenyl-4*H*-1,2,4-triazole-3-thiol	$C_9H_9N_3S$	95%	Chembridge
	Triphenylphosphine	PPh_3	99%	Sigma-Aldrich
	DIAD	$(CH_3)_2CHOOCN{=}NCOOCH(CH_3)_2$	98%	Sigma-Aldrich
	Iodomethane	CH_3I	99.5%	Sigma-Aldrich
	3-Bromopropylamine hydrobromide	$BrCH_2CH_2CH_2NH_2 \cdot HBr$	98%	Sigma-Aldrich
	N-Methylethanamine	$C_2H_5NHCH_3$	97%	Sigma-Aldrich

	Ethylenediamine	$C_2H_8N_2$	>98.0%	TCI
	Ethyl trifluoroacetate	$C_4H_5F_3O_2$	>99.0%	TCI
	Allyl bromide	$CH_2=CHCH_2Br$	97%	Sigma-Aldrich
	Di-*tert*-butyl dicarbonate ((Boc)$_2$O)	$[(CH_3)_3COCO]_2O$	>98.0%	Sigma-Aldrich
	4-Fluorobenzaldehyde	FC_6H_4CHO	98%	Sigma-Aldrich
	2-Naphthoyl chloride	$C_{10}H_7COCl$	98%	Sigma-Aldrich
	4-Quinolinecarboxylic acid	$C_{10}H_7NO_2$	97%	Sigma-Aldrich
	4-(4-Pyridiyl)benzoic acid	$C_{12}H_9NO_2$	≥99%	Sigma-Aldrich
	Indole-2-carboxylic acid	$C_9H_7NO_2$	98%	Sigma-Aldrich
	Imidazo[1,2-*a*]pyridine-2-carboxylic acid	$C_8H_6N_2O_2$	85%	Sigma-Aldrich
	Isonicotinic acid	$C_6H_5NO_2$	99%	Sigma-Aldrich
	4-(Thiophen-3-yl)benzoic acid	$C_{11}H_8O_2S$	95%	Ambeed
	3-(Dimethylamino)benzoic acid	$(CH_3)_2NC_6H_4CO_2H$	≥97%	Sigma-Aldrich
	3-Thiophenecarboxylic acid	$C_5H_4O_2S$	99%	Sigma-Aldrich
	1-Methylindole-2-carboxylic acid	$C_{10}H_9NO_2$	98%	Sigma-Aldrich
Solvents	DMF	$HCON(CH_3)_2$	99.8%	Sigma-Aldrich
	Dichloromethane	CH_2Cl_2	≥99.8%	Sigma-Aldrich
	MeCN	CH_3CN	99.8%	Sigma-Aldrich
	THF	C_4H_8O	≥99.9%	Sigma-Aldrich
	Dichloroethane	$ClCH_2CH_2Cl$	99.8%	Acros organics
	EtOH	CH_3CH_2OH	100%	Decon laboratories
	Acetone	CH_3COCH_3	≥99.5%	Sigma-Aldrich
	MeOH	CH_3OH	99.8%	Sigma-Aldrich

Analyses of HRMS

Compound: 6a

Single Mass Analysis
Tolerance = 3.0 mDa / DBE: min = -1.5, max = 50.0
Element prediction: Off
Number of isotope peaks used for i-FIT = 2

Monoisotopic Mass, Even Electron Ions
989 formula(e) evaluated with 4 results within limits (up to 100 closest results for each mass)
Elements Used:
C: 0-52 H: 0-103 N: 0-3 O: 0-9 F: 1-3 23Na: 0-1

24-Nov-2019 KIM
HY_I_085 32 (0.941) 1: TOF MS ES+
3.89e+003

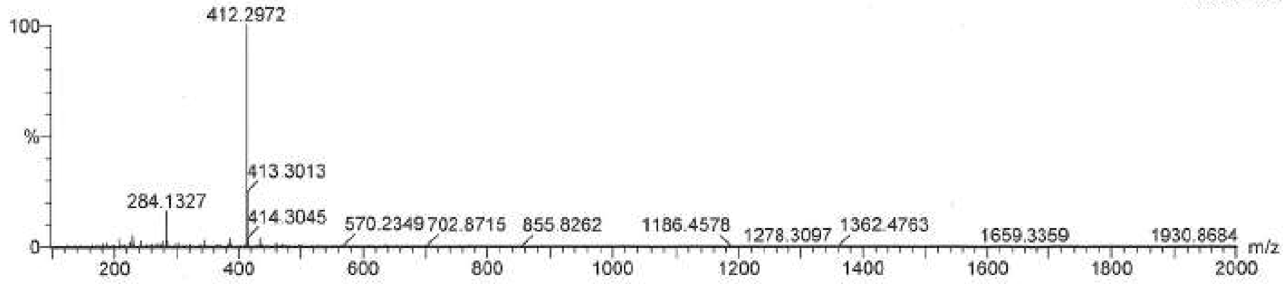

Compound: 6b

Single Mass Analysis
Tolerance = 3.0 mDa / DBE: min = -1.5, max = 50.0
Element prediction: Off
Number of isotope peaks used for i-FIT = 3

Monoisotopic Mass, Even Electron Ions
1239 formula(e) evaluated with 3 results within limits (up to 50 best isotopic matches for each mass)
Elements Used:
C: 0-80 H: 0-160 N: 0-12 O: 0-11 23Na: 0-1 F: 1-1

13-Dec-202207:03:06
HY_1_83 23 (1.128) 1: TOF MS ES+
5.36e+002

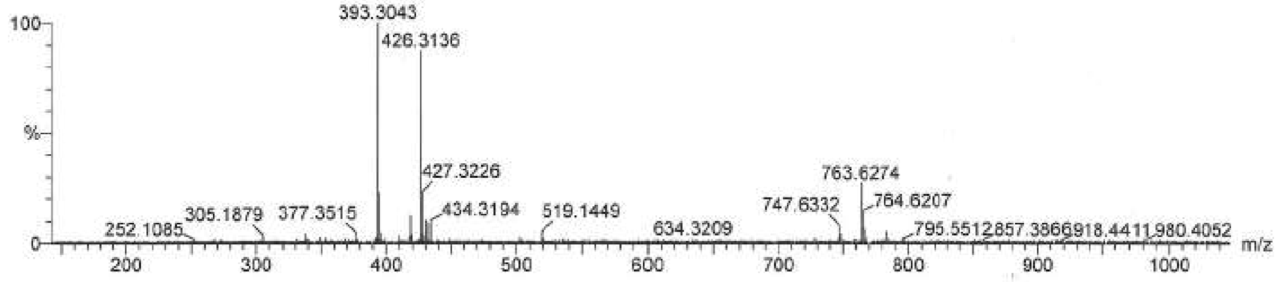

Compound: 6c

Single Mass Analysis
Tolerance = 3.0 mDa / DBE: min = -1.5, max = 50.0
Element prediction: Off
Number of isotope peaks used for i-FIT = 3

Monoisotopic Mass, Even Electron Ions
1027 formula(e) evaluated with 4 results within limits (up to 50 best isotopic matches for each mass)
Elements Used:
C: 0-80 H: 0-160 N: 0-12 O: 0-11 23Na: 0-1 Br: 1-1

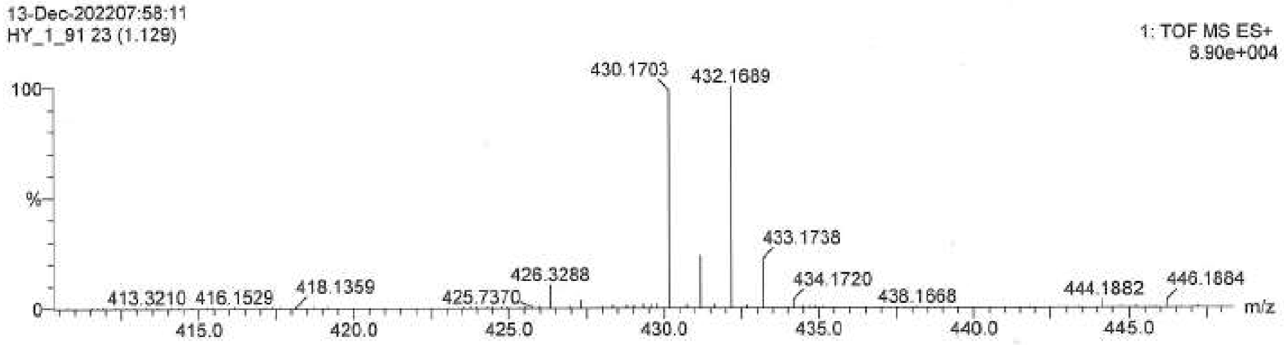

Compound: 6d

Single Mass Analysis
Tolerance = 10.0 mDa / DBE: min = -1.5, max = 50.0
Element prediction: Off
Number of isotope peaks used for i-FIT = 2

Monoisotopic Mass, Even Electron Ions
148 formula(e) evaluated with 4 results within limits (up to 100 closest results for each mass)
Elements Used:
C: 0-20 H: 0-50 N: 0-5 C: 0-5 23Na: 0-1 Br: 1-1

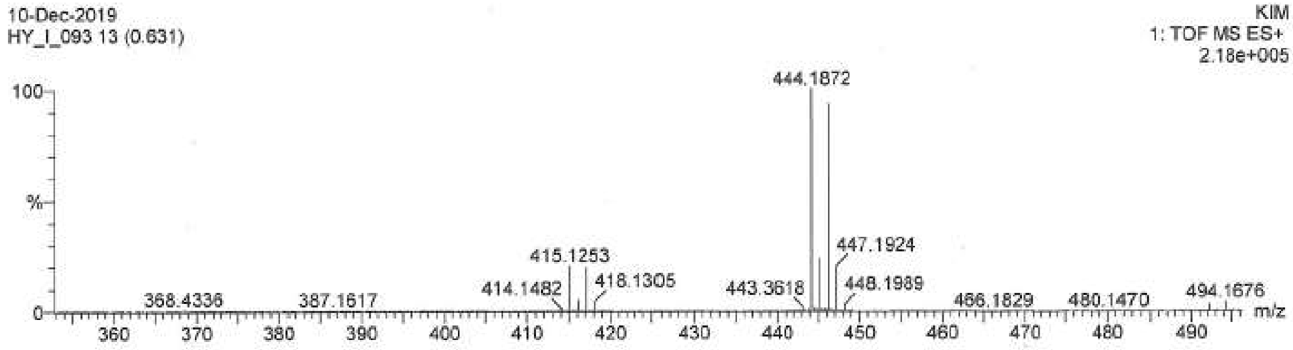

Compound: 9a

Single Mass Analysis
Tolerance = 3.0 mDa / DBE: min = -1.5, max = 100.0
Element prediction: Off
Number of isotope peaks used for i-FIT = 3

Monoisotopic Mass, Even Electron Ions
1179 formula(e) evaluated with 8 results within limits (up to 10 best isotopic matches for each mass)
Elements Used:
C: 0-50 H: 0-100 N: 0-10 O: 0-10 23Na: 0-1 S: 1-1 Br: 1-1

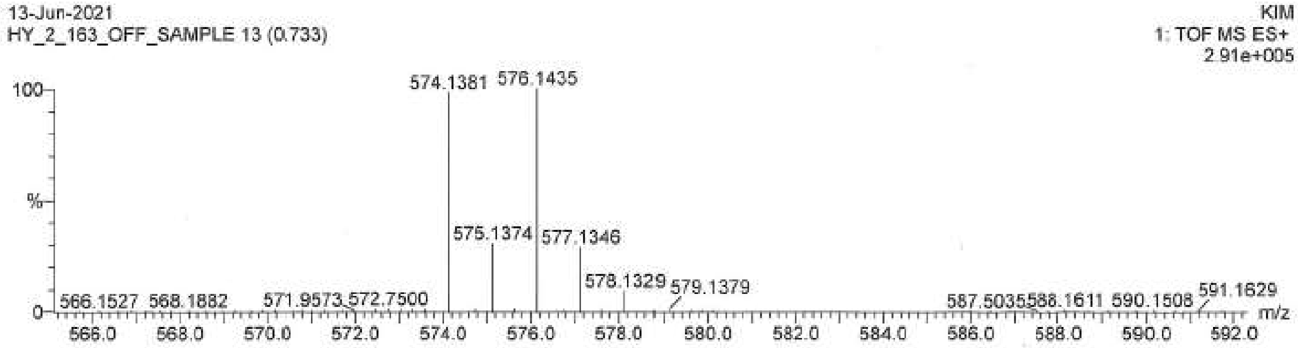

Compound: 9b

Single Mass Analysis
Tolerance = 4.0 mDa / DBE: min = -1.5, max = 50.0
Element prediction: Off
Number of isotope peaks used for i-FIT = 3

Monoisotopic Mass, Even Electron Ions
2209 formula(e) evaluated with 11 results within limits (up to 50 best isotopic matches for each mass)
Elements Used:
C: 0-50 H: 0-100 N: 0-6 O: 0-6 F: 0-1 23Na: 0-1 S: 0-1 Br: 1-1

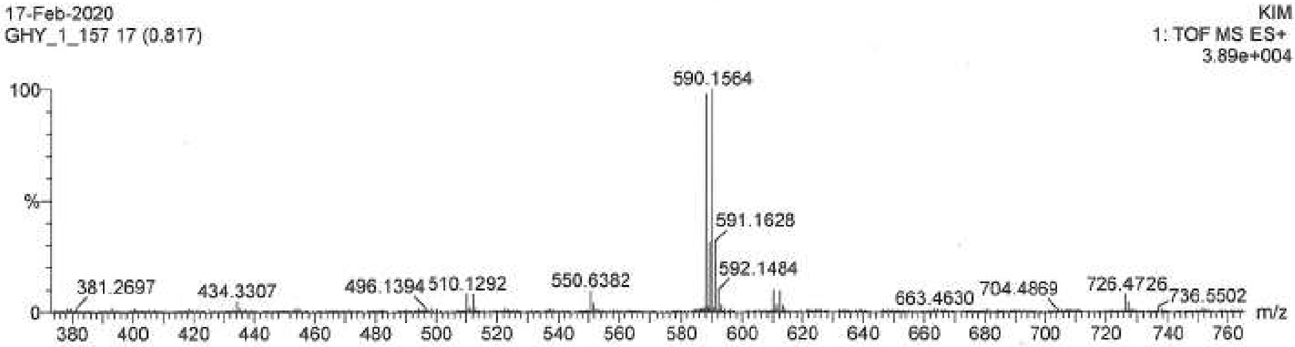

Compound: 11a

Single Mass Analysis
Tolerance = 3.0 mDa / DBE: min = -1.5, max = 100.0
Element prediction: Off
Number of isotope peaks used for i-FIT = 3

Monoisotopic Mass, Even Electron Ions
1177 formula(e) evaluated with 5 results within limits (up to 10 best isotopic matches for each mass)
Elements Used:
C: 0-50 H: 0-100 N: 0-10 O: 0-10 23Na: 0-1 S: 1-1 Br: 1-1

27-Feb-2021 KIM
HY_2_174_2 54 (1.597) Cm (54:57) 1: TOF MS ES+
2.31e+004

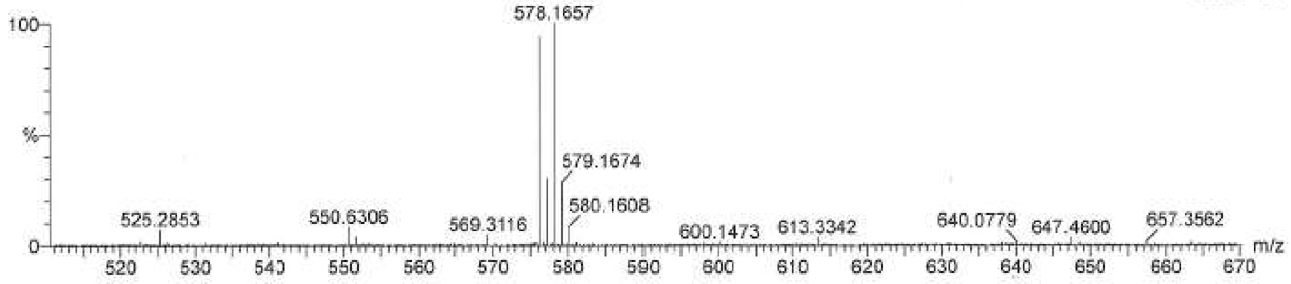

Compound: 11b

Single Mass Analysis
Tolerance = 3.0 mDa / DBE: min = -1.5, max = 50.0
Element prediction: Off
Number of isotope peaks used for i-FIT = 3

Monoisotopic Mass, Even Electron Ions
1221 formula(e) evaluated with 6 results within limits (up to 50 best isotopic matches for each mass)
Elements Used:
C: 0-100 H: 0-200 N: 0-10 O: 0-10 23Na: 0-1 S: 1-1 Br: 1-1

13-Feb-2020 KIM
HY_I_157 12 (0.596) 1: TOF MS ES+
3.30e+004

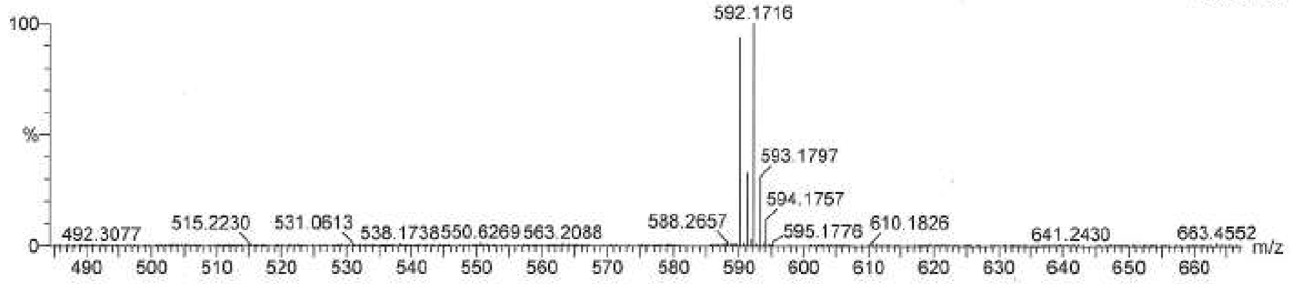

Compound: 12

Single Mass Analysis
Tolerance = 3.0 mDa / DBE: min = -1.5, max = 100.0
Element prediction: Off
Number of isotope peaks used for i-FIT = 3

Monoisotopic Mass, Even Electron Ions
619 formula(e) evaluated with 4 results within limits (up to 10 best isotopic matches for each mass)
Elements Used:
C: 0-50 H: 0-92 N: 0-10 O: 0-13 23Na: 0-1 Br: 1-1

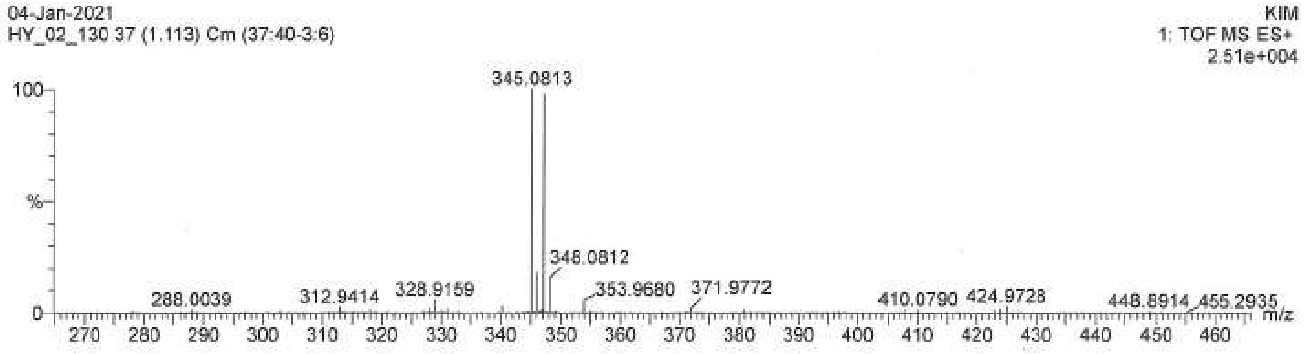

Compound: 14

Single Mass Analysis
Tolerance = 3.0 mDa / DBE: min = -1.5, max = 100.0
Element prediction: Off
Number of isotope peaks used for i-FIT = 3

Monoisotopic Mass, Even Electron Ions
224 formula(e) evaluated with 1 results within limits (up to 10 best isotopic matches for each mass)
Elements Used:
C: 17-17 H: 0-100 N: 0-10 O: 0-10 23Na: 0-1 S: 1-1

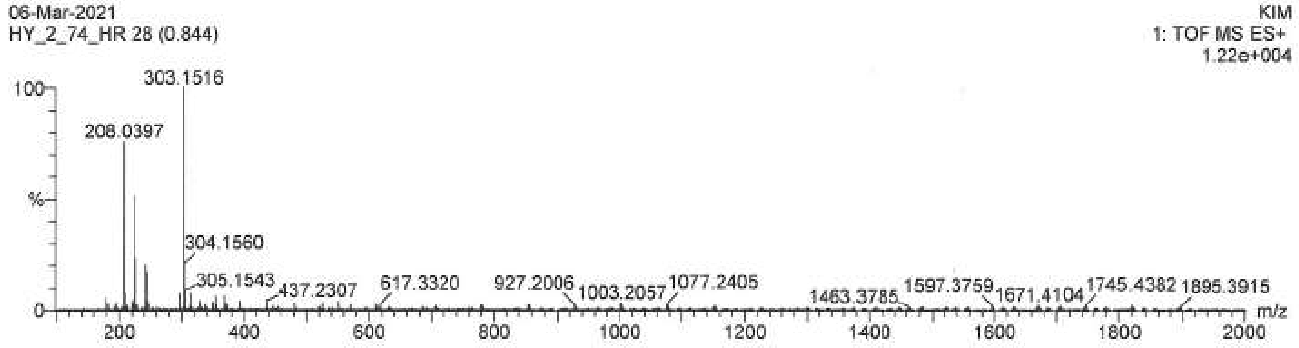

Compound: 20a

Single Mass Analysis
Tolerance = 2.0 mDa / DBE: min = -1.5, max = 100.0
Element prediction: Off
Number of isotope peaks used for i-FIT = 3

Monoisotopic Mass, Even Electron Ions
2724 formula(e) evaluated with 8 results within limits (up to 25 best isotopic matches for each mass)
Elements Used:
C: 0-108 H: 0-200 N: 0-16 O: 0-24 23Na: 0-1 S: 1-1 Br: 1-1

03-Dec-2020 KIM
HY_02_109 16 (0.773) 1: TOF MS ES+
7.09e+004

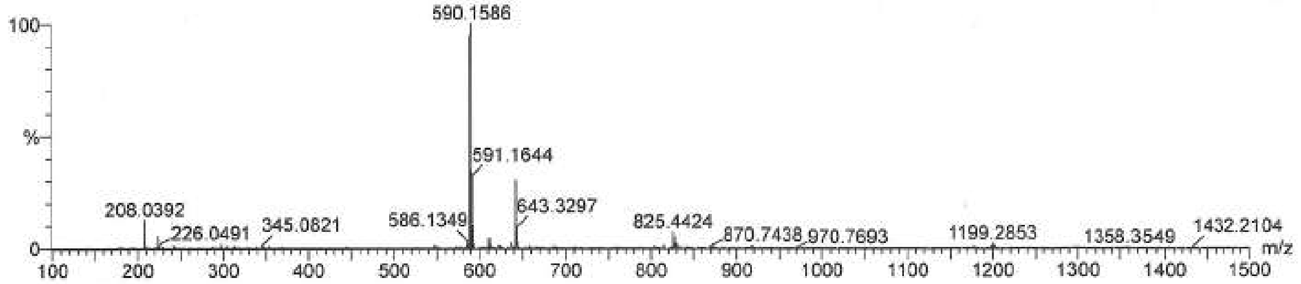

Compound: 20b

Single Mass Analysis
Tolerance = 3.0 PPM / DBE: min = -1.5, max = 100.0
Element prediction: Off
Number of isotope peaks used for i-FIT = 3

Monoisotopic Mass, Even Electron Ions
547 formula(e) evaluated with 2 results within limits (up to 25 best isotopic matches for each mass)
Elements Used:
C: 0-108 H: 0-200 N: 0-6 O: 0-6 23Na: 0-1 S: 1-1 Br: 1-1

05-Dec-2020 KIM
HY_02_114 28 (0.844) Cm (28:29) 1: TOF MS ES+
1.66e+004

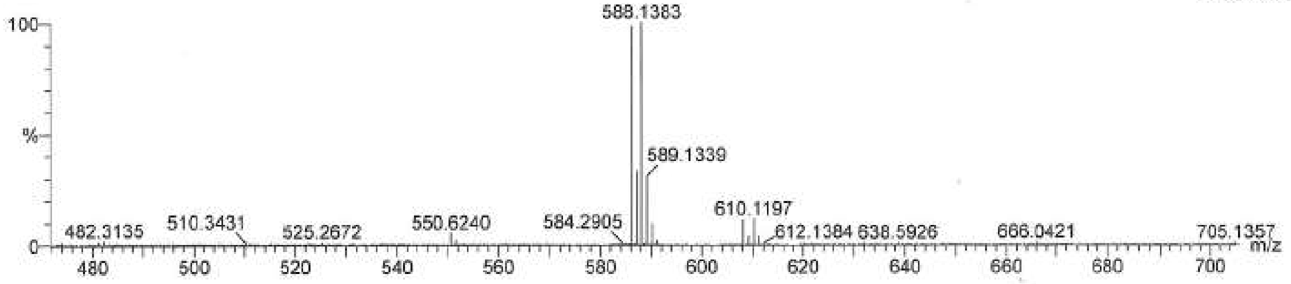

Compound: 20c

Single Mass Analysis
Tolerance = 3.0 mDa / DBE: min = -1.5, max = 100.0
Element prediction: Off
Number of isotope peaks used for i-FIT = 3

Monoisotopic Mass, Even Electron Ions
1609 formula(e) evaluated with 9 results within limits (up to 10 best isotopic matches for each mass)
Elements Used:
C: 0-50 H: 0-92 N: 0-10 O: 0-13 23Na: 0-1 S: 1-1 Br: 1-1 F: 1-1

13-Jan-2021
HY_2_134 47 (1.409) Cm (47:48)

KIM
1: TOF MS ES+
2.80e+004

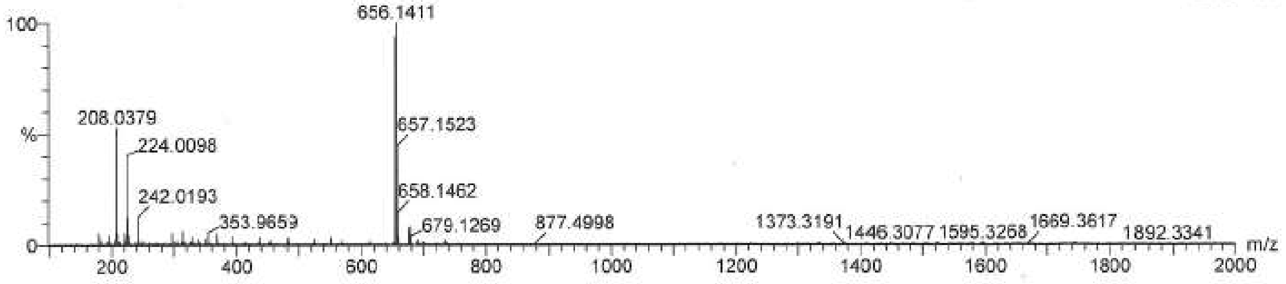

Compound: 21a

Single Mass Analysis
Tolerance = 3.0 mDa / DBE: min = -1.5, max = 100.0
Element prediction: Off
Number of isotope peaks used for i-FIT = 3

Monoisotopic Mass, Even Electron Ions
1051 formula(e) evaluated with 6 results within limits (up to 25 best isotopic matches for each mass)
Elements Used:
C: 0-59 H: 0-75 N: 0-8 O: 0-11 23Na: 0-1 Br: 1-1

24-Nov-2020
HY_02_090_24nov2020 13 (0.641)

KIM
1: TOF MS ES+
5.15e+004

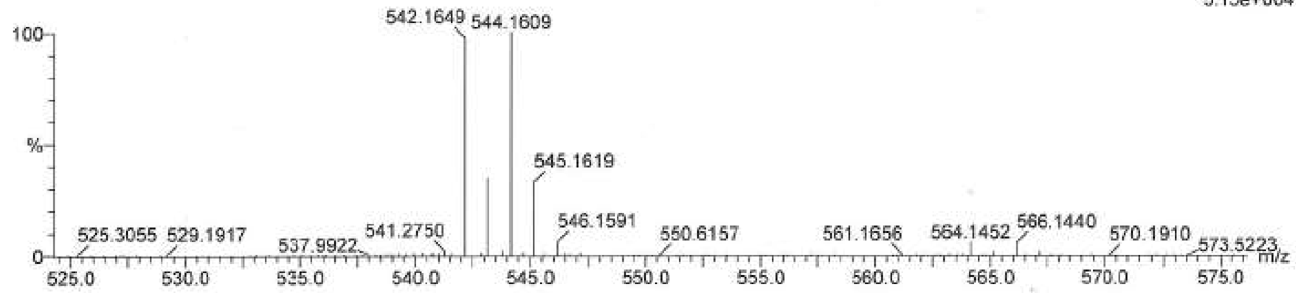

Compound: 21b

Single Mass Analysis

Tolerance = 3.0 mDa / DBE: min = -1.5, max = 100.0
Element prediction: Off
Number of isotope peaks used for i-FIT = 3

Monoisotopic Mass, Even Electron Ions
1049 formula(e) evaluated with 8 results within limits (up to 25 best isotopic matches for each mass)
Elements Used:
C: 0-59 H: 0-75 N: 0-8 O: 0-11 23Na: 0-1 Br: 1-1

24-Nov-2020 KIM
HY_02_091 12 (0.596) 1: TOF MS ES+
4.35e+004

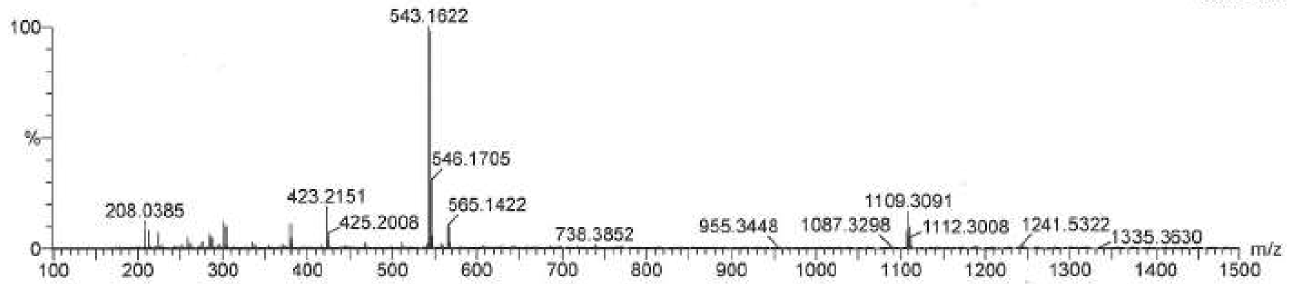

Compound: 21c

Single Mass Analysis

Tolerance = 3.0 mDa / DBE: min = -1.5, max = 100.0
Element prediction: Off
Number of isotope peaks used for i-FIT = 3

Monoisotopic Mass, Even Electron Ions
1116 formula(e) evaluated with 8 results within limits (up to 25 best isotopic matches for each mass)
Elements Used:
C: 0-59 H: 0-75 N: 0-8 O: 0-11 23Na: 0-1 Br: 1-1

24-Nov-2020 KIM
HY_02_092 6 (0.284) 1: TOF MS ES+
2.38e+005

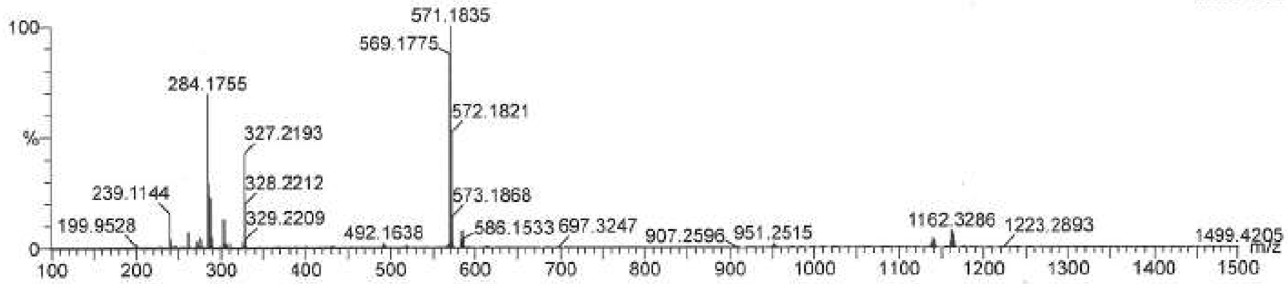

Compound: 21d

Single Mass Analysis
Tolerance = 3.0 mDa / DBE: min = -1.5, max = 100.0
Element prediction: Off
Number of isotope peaks used for i-FIT = 3

Monoisotopic Mass, Even Electron Ions
1018 formula(e) evaluated with 9 results within limits (up to 25 best isotopic matches for each mass)
Elements Used:
C: 0-59 H: 0-75 N: 0-8 O: 0-11 23Na: 0-1 Br: 1-1

24-Nov-2020
HY_02_093 18 (0.861)

KIM
1: TOF MS ES+
7.13e+003

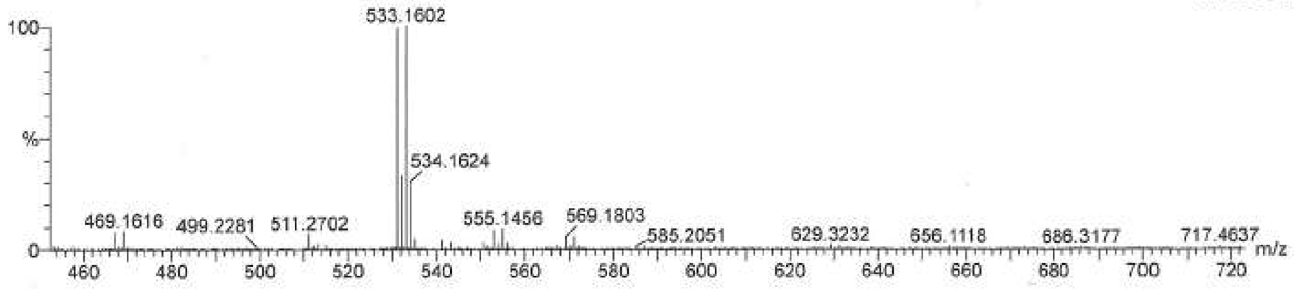

Compound: 21e

Single Mass Analysis
Tolerance = 3.0 mDa / DBE: min = -1.5, max = 100.0
Element prediction: Off
Number of isotope peaks used for i-FIT = 3

Monoisotopic Mass, Even Electron Ions
1020 formula(e) evaluated with 5 results within limits (up to 25 best isotopic matches for each mass)
Elements Used:
C: 0-59 H: 0-75 N: 0-8 O: 0-11 23Na: 0-1 Br: 1-1

24-Nov-2020
HY_02_095 18 (0.861)

KIM
1: TOF MS ES+
9.47e+002

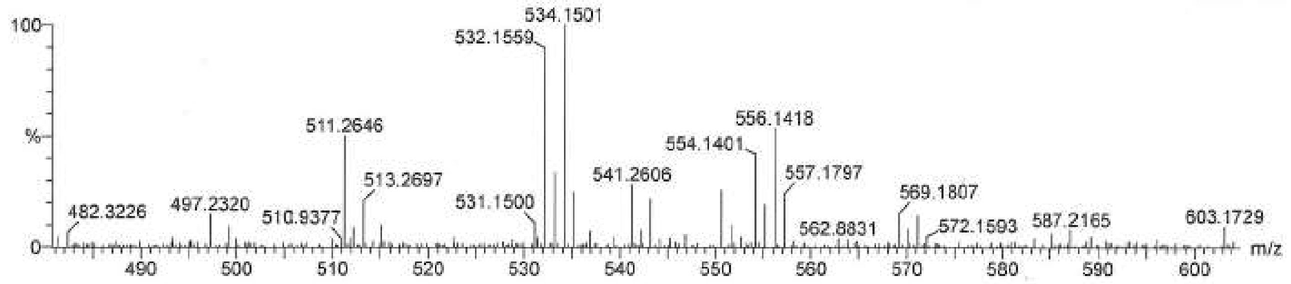

Compound: 21f

Single Mass Analysis
Tolerance = 2.0 mDa / DBE: min = -1.5, max = 100.0
Element prediction: Off
Number of isotope peaks used for i-FIT = 3

Monoisotopic Mass, Even Electron Ions
2045 formula(e) evaluated with 8 results within limits (up to 25 best isotopic matches for each mass)
Elements Used:
C: 0-108 H: 0-200 N: 0-16 O: 0-24 23Na: 0-1 Br: 1-1

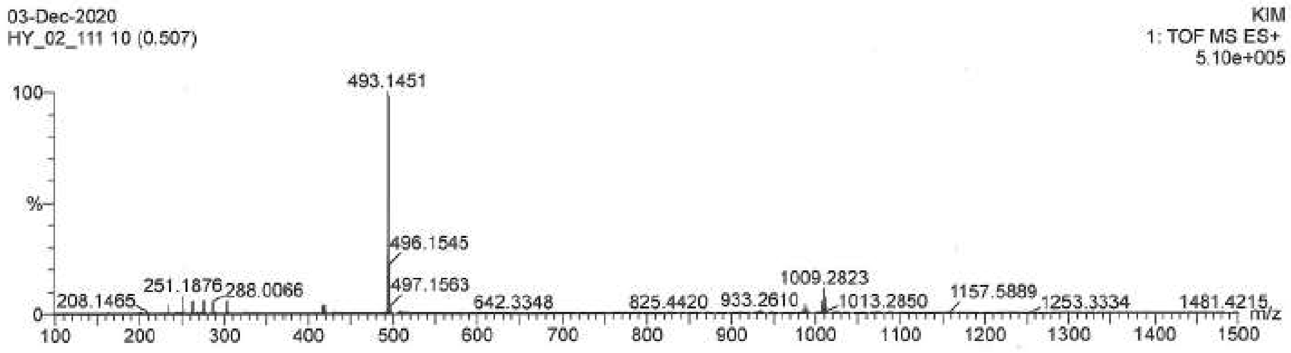

Compound: 21g

Single Mass Analysis
Tolerance = 3.0 mDa / DBE: min = -1.5, max = 100.0
Element prediction: Off
Number of isotope peaks used for i-FIT = 3

Monoisotopic Mass, Even Electron Ions
1179 formula(e) evaluated with 6 results within limits (up to 10 best isotopic matches for each mass)
Elements Used:
C: 0-50 H: 0-100 N: 0-10 O: 0-10 23Na: 0-1 S: 1-1 Br: 1-1

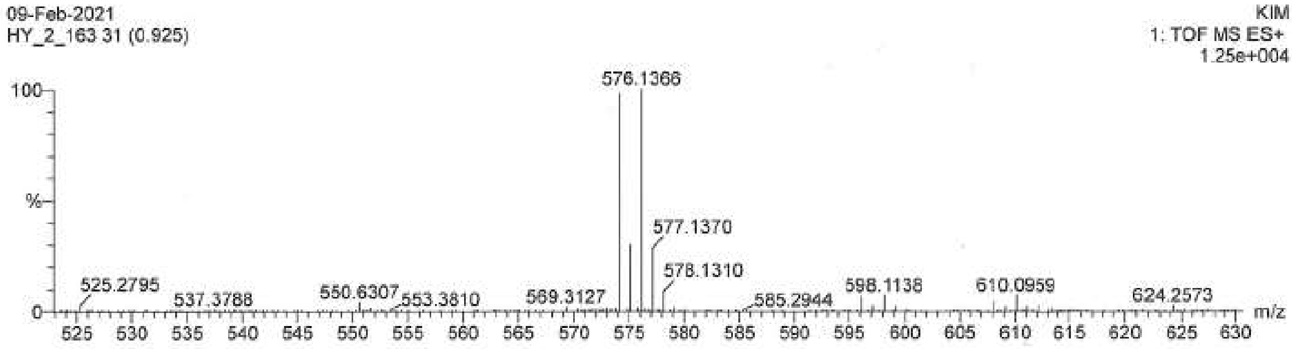

Compound: 21h

Single Mass Analysis
Tolerance = 3.0 mDa / DBE: min = -1.5, max = 50.0
Element prediction: Off
Number of isotope peaks used for i-FIT = 3

Monoisotopic Mass, Even Electron Ions
1426 formula(e) evaluated with 8 results within limits (up to 50 best isotopic matches for each mass)
Elements Used:
C: 0-80 H: 0-160 N: 0-12 O: 0-11 23Na: 0-1 Br: 1-1

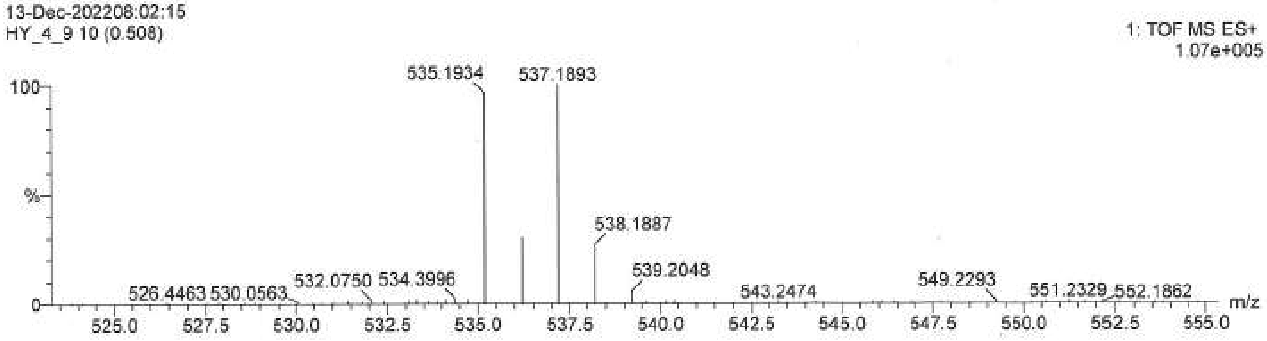

Compound: 21i

Single Mass Analysis
Tolerance = 3.0 mDa / DBE: min = -1.5, max = 100.0
Element prediction: Off
Number of isotope peaks used for i-FIT = 3

Monoisotopic Mass, Even Electron Ions
2007 formula(e) evaluated with 14 results within limits (up to 10 best isotopic matches for each mass)
Elements Used:
C: 0-50 H: 0-100 N: 0-10 O: 0-10 23Na: 0-1 S: 0-1 Br: 1-1

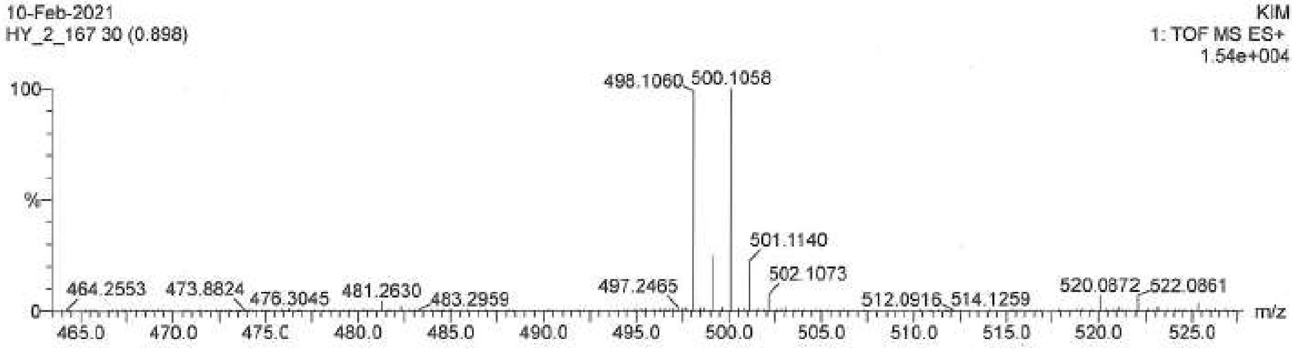

Compound: 21j

Single Mass Analysis
Tolerance = 3.0 mDa / DBE: min = -1.5, max = 100.0
Element prediction: Off
Number of isotope peaks used for i-FIT = 3

Monoisotopic Mass, Even Electron Ions
1181 formula(e) evaluated with 8 results within limits (up to 10 best isotopic matches for each mass)
Elements Used:
C: 0-50 H: 0-100 N: 0-10 O: 0-10 23Na: 0-1 Br: 1-1

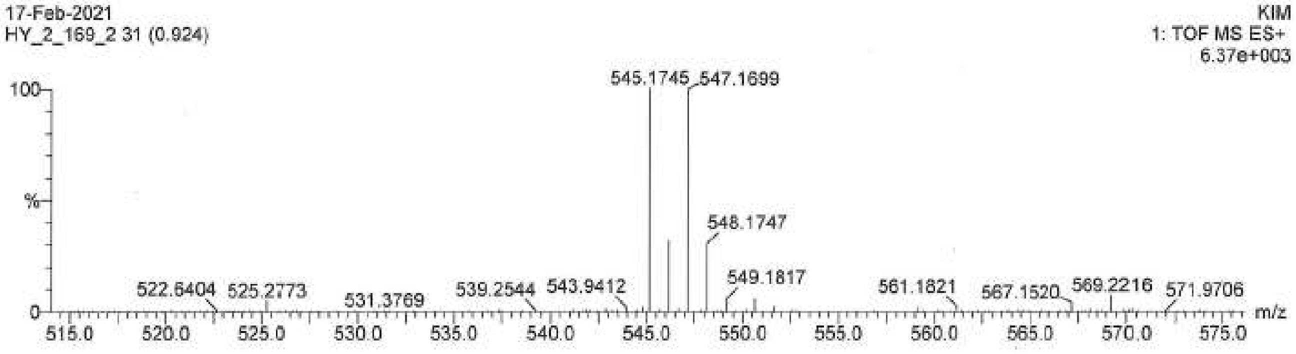

^{1}H or ^{13}C NMR spectra

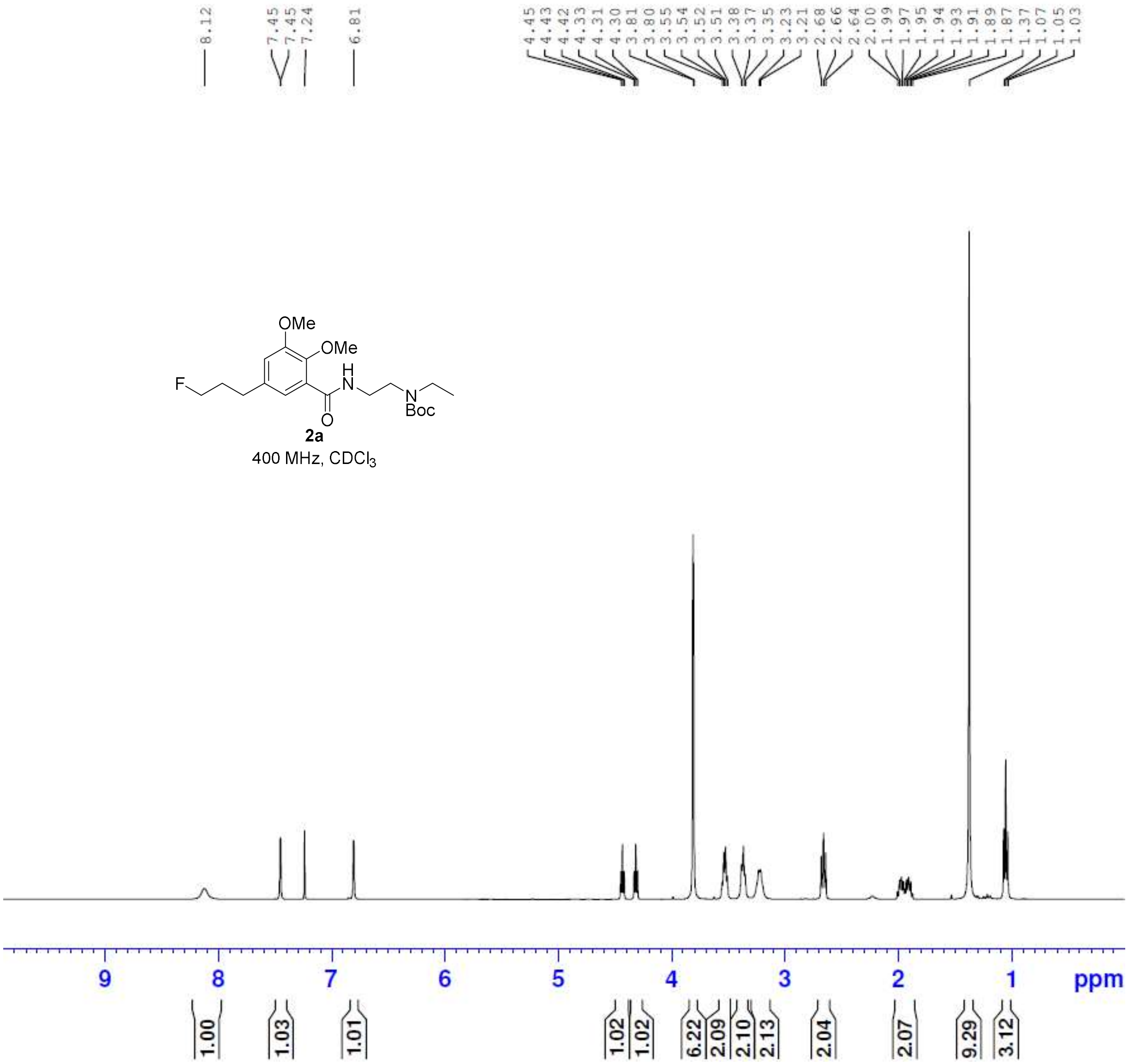

8.12
7.45
7.45
7.24
6.81
4.45
4.43
4.42
4.33
4.31
4.30
3.81
3.80
3.55
3.54
3.52
3.51
3.38
3.37
3.35
3.23
3.21
2.68
2.66
2.64
2.00
1.99
1.97
1.95
1.94
1.93
1.91
1.89
1.87
1.37
1.07
1.05
1.03
OMe
OMe
H
N
F
O
N
Boc
2a
400 MHz, CDCl3
9
8
7
6
5
4
3
2
1
ppm
1.00
1.03
1.01
1.02
1.02
6.22
2.09
2.10
2.13
2.04
2.07
9.29
3.12

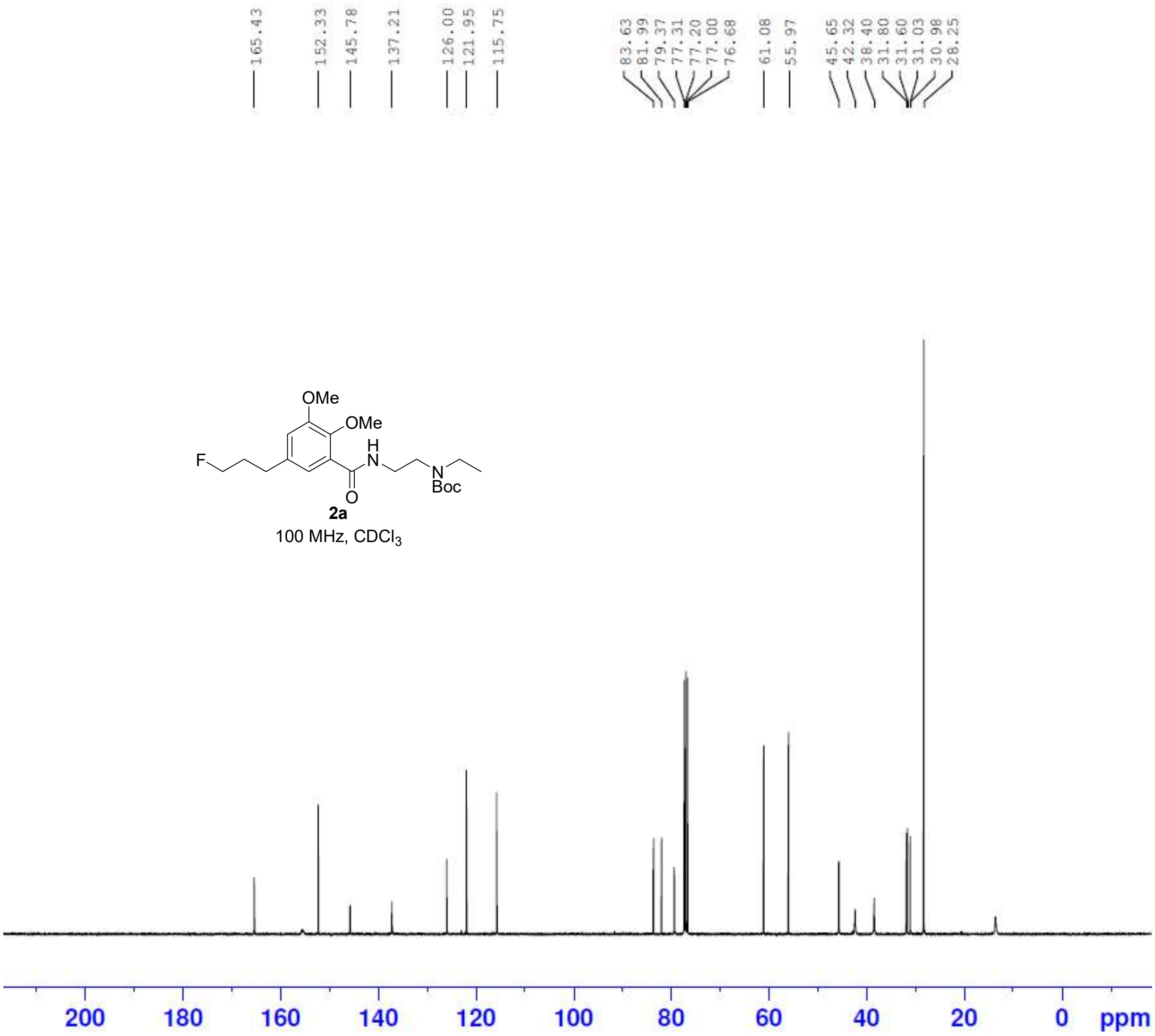

165.43
152.33
145.78
137.21
126.00
121.95
115.75
83.63
81.99
79.37
77.31
77.20
77.00
76.68
61.08
55.97
45.65
42.32
38.40
31.80
31.60
31.03
30.98
28.25
OMe
OMe
H
N
F
N
Boc
O
2a
100 MHz, $CDCl_3$
200
180
160
140
120
100
80
60
40
20
0
ppm

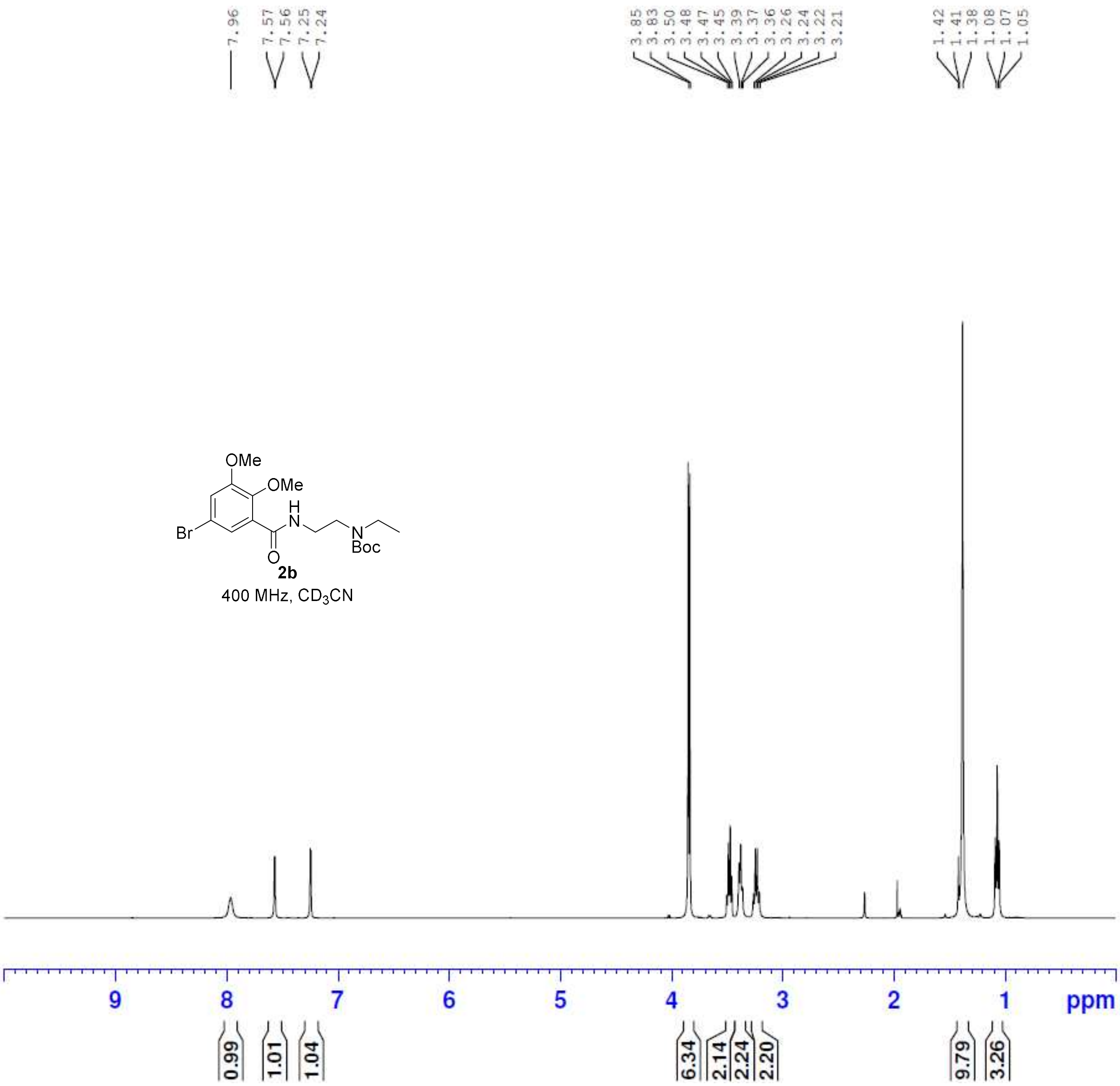
7.96
7.57
7.56
7.25
7.24
3.85
3.83
3.50
3.48
3.47
3.45
3.39
3.37
3.36
3.26
3.24
3.22
3.21
1.42
1.41
1.38
1.08
1.07
1.05
OMe
OMe
H
N
Br
O
N
Boc
2b
400 MHz, CD_3CN
9
8
7
6
5
4
3
2
1
ppm
0.99
1.01
1.04
6.34
2.14
2.24
2.20
9.79
3.26

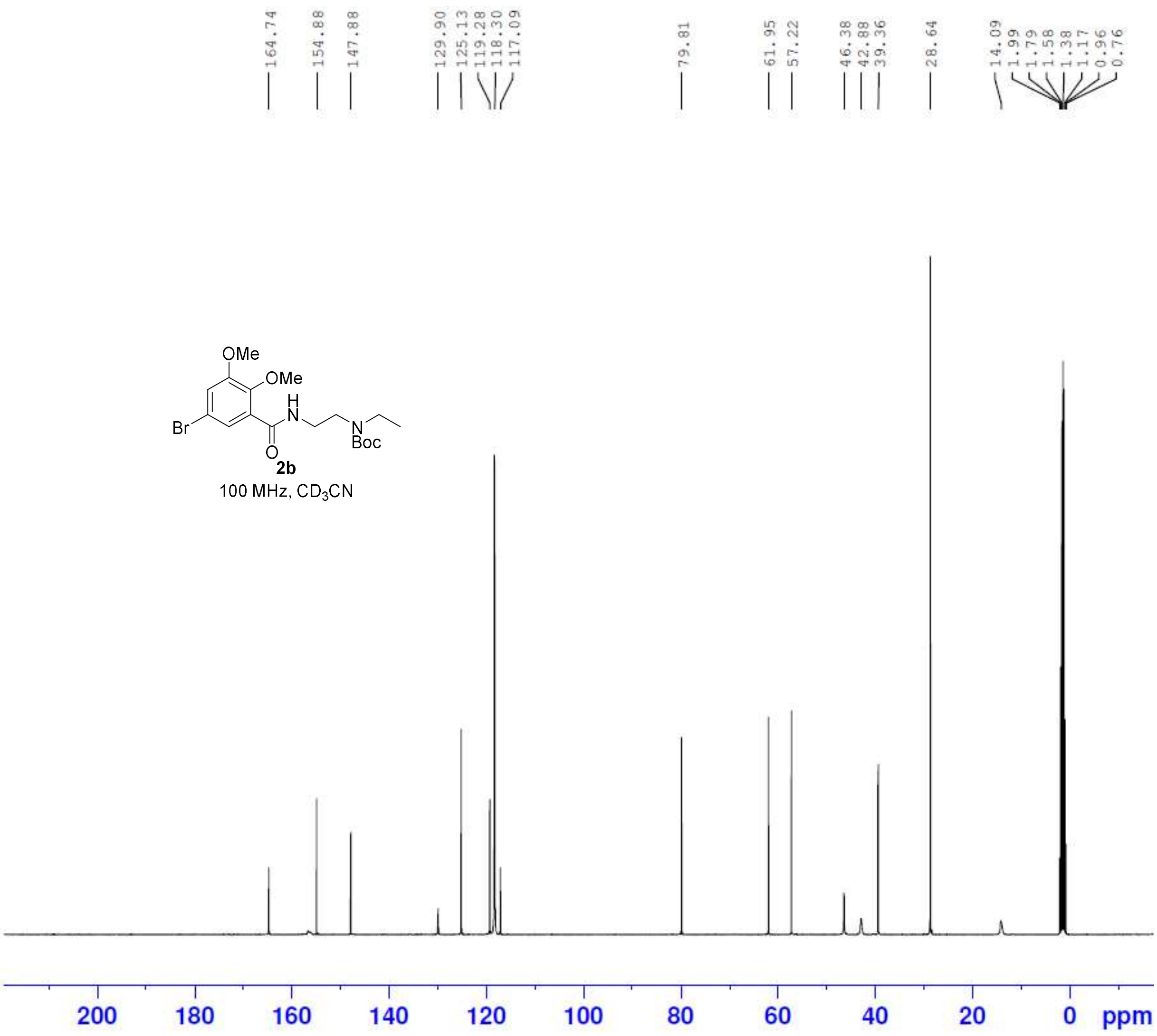
164.74
154.88
147.88
129.90
125.13
119.28
118.30
117.09
79.81
61.95
57.22
46.38
42.88
39.36
28.64
14.09
1.99
1.79
1.58
1.38
1.17
0.96
0.76
OMe
OMe
H
N
Br
O
N
Boc
2b
100 MHz, CD_3CN
200
180
160
140
120
100
80
60
40
20
0
ppm

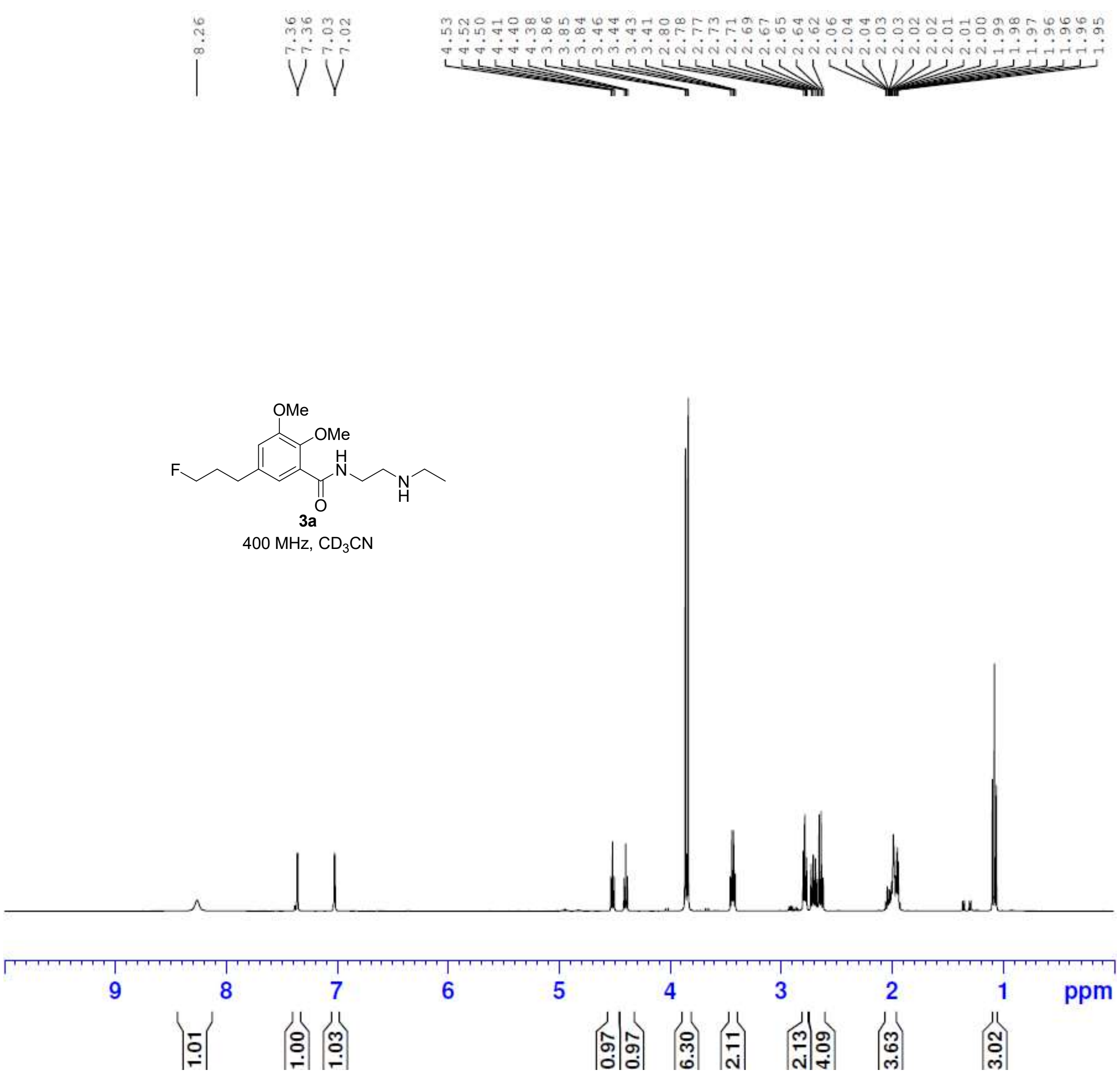

8.26
7.36
7.36
7.03
7.02
4.53
4.52
4.50
4.41
4.40
4.38
3.86
3.85
3.84
3.46
3.44
3.43
3.41
2.80
2.78
2.77
2.73
2.71
2.69
2.67
2.65
2.64
2.62
2.06
2.04
2.03
2.03
2.02
2.02
2.01
2.01
2.00
1.99
1.98
1.97
1.96
1.96
1.95
OMe
OMe
F
H
N
N
H
O
3a
400 MHz, CD_3CN
9
8
7
6
5
4
3
2
1
ppm
1.01
1.00
1.03
0.97
0.97
6.30
2.11
2.13
4.09
3.63
3.02

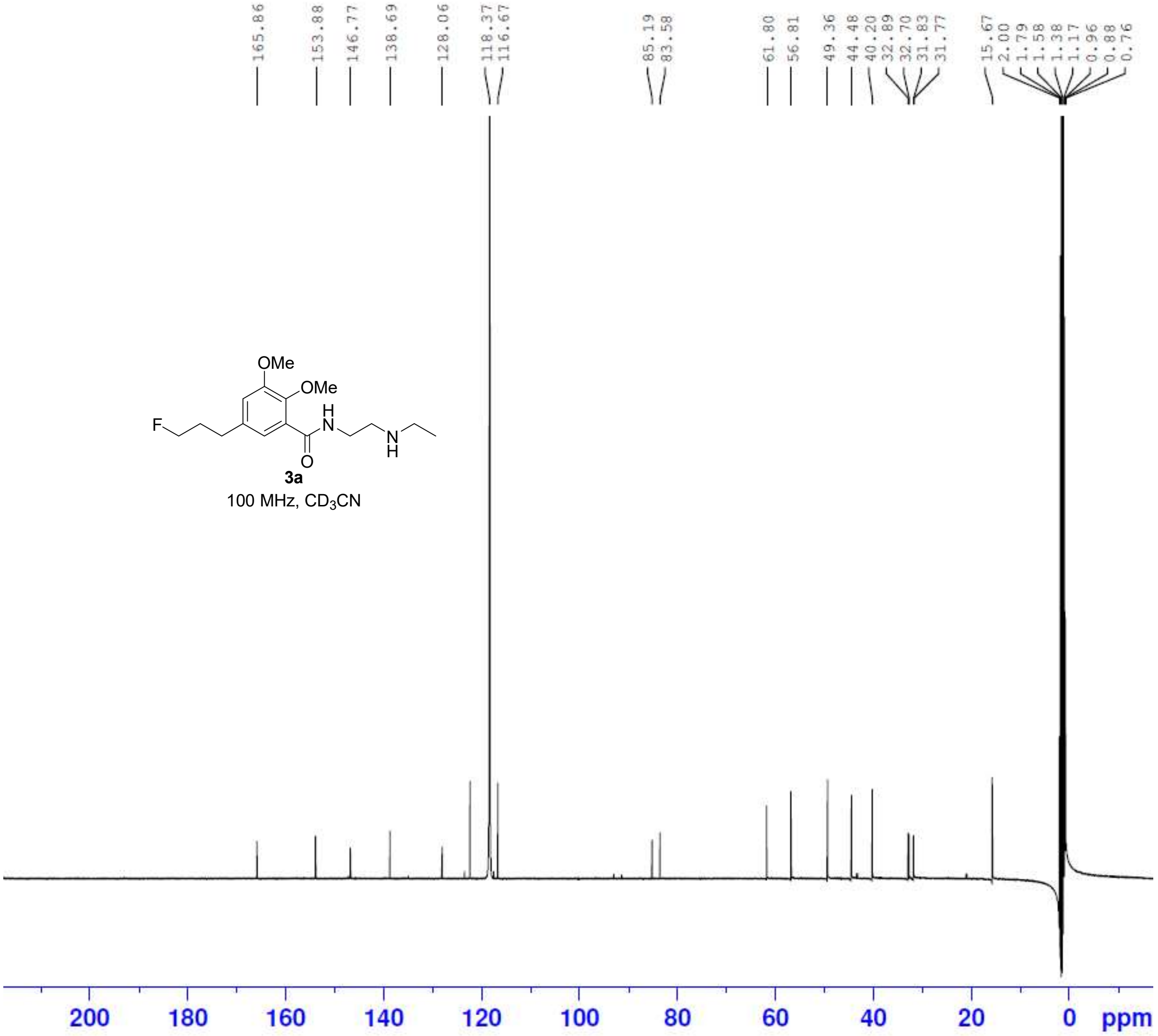
165.86
153.88
146.77
138.69
128.06
118.37
116.67
85.19
83.58
61.80
56.81
49.36
44.48
40.20
32.89
32.70
31.83
31.77
15.67
2.00
1.79
1.58
1.38
1.17
0.96
0.88
0.76
OMe
OMe
F
H
N
O
N
H
3a
100 MHz, CD_3CN
200
180
160
140
120
100
80
60
40
20
0
ppm

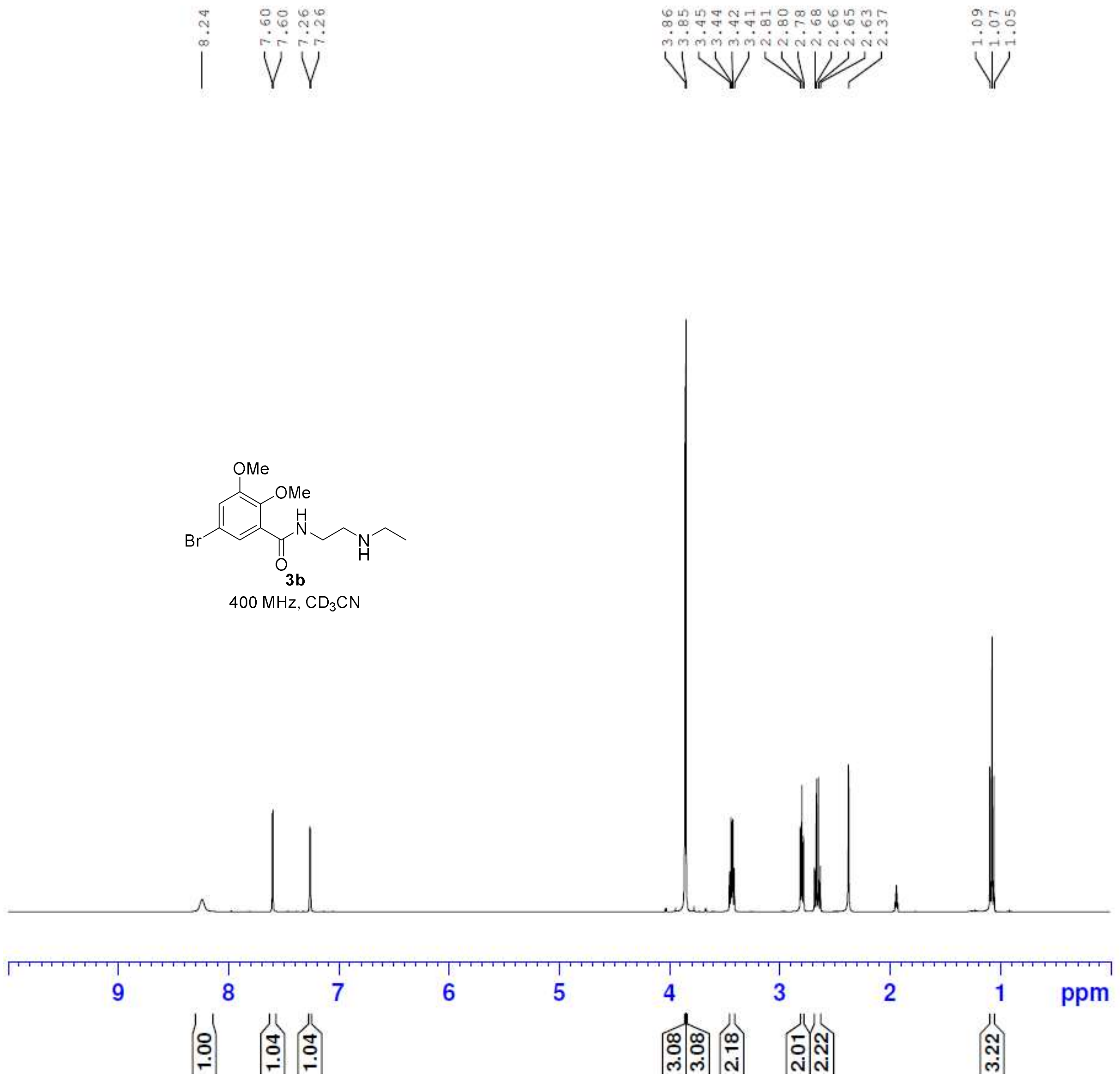
8.24
7.60
7.60
7.26
7.26
3.86
3.85
3.45
3.44
3.42
3.41
2.81
2.80
2.78
2.68
2.66
2.65
2.63
2.37
1.09
1.07
1.05
OMe
OMe
H
N
Br
O
N
H
3b
400 MHz, CD_3CN
9
8
7
6
5
4
3
2
1
ppm
1.00
1.04
1.04
3.08
3.08
2.18
2.01
2.22
3.22

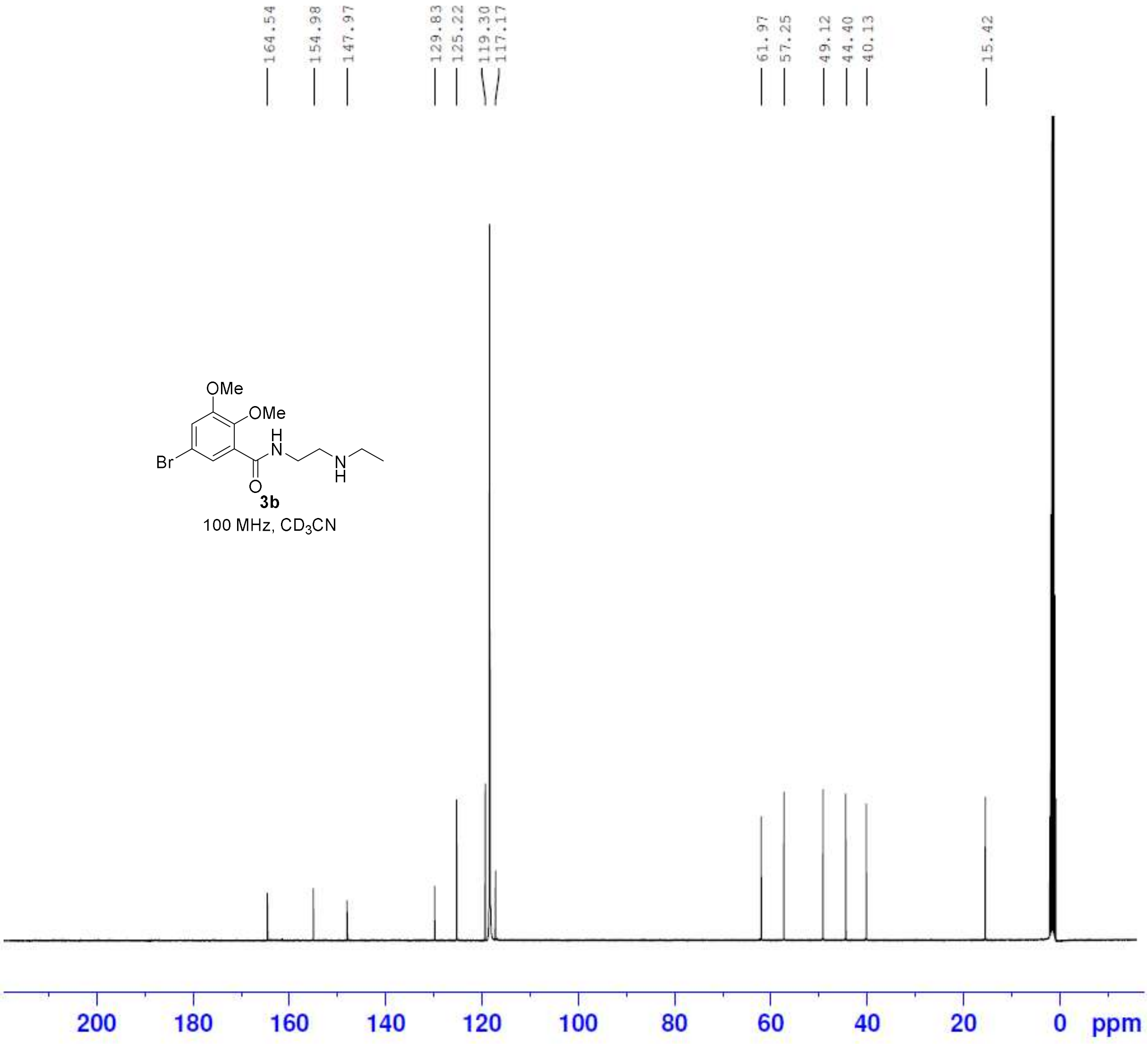
164.54
154.98
147.97
129.83
125.22
119.30
117.17
61.97
57.25
49.12
44.40
40.13
15.42
OMe
OMe
Br
H
N
N
H
O
3b
100 MHz, CD_3CN
200
180
160
140
120
100
80
60
40
20
0
ppm

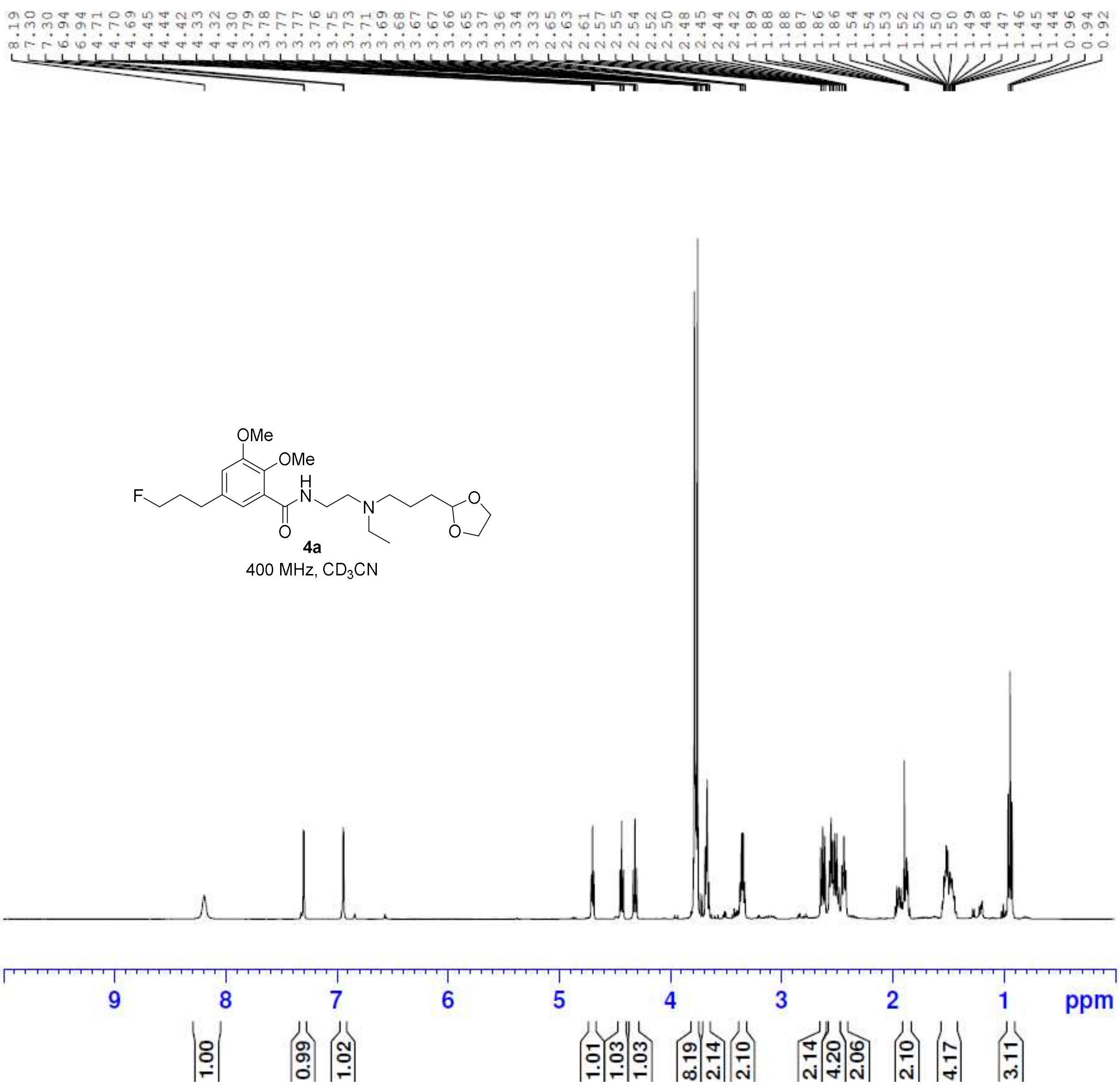
OMe
OMe
H
N
F
N
O
O
O
4a
400 MHz, CD3CN
9
8
7
6
5
4
3
2
1
ppm
1.00
0.99
1.02
1.01
1.03
1.03
8.19
2.14
2.10
2.14
4.20
2.06
2.10
4.17
3.11

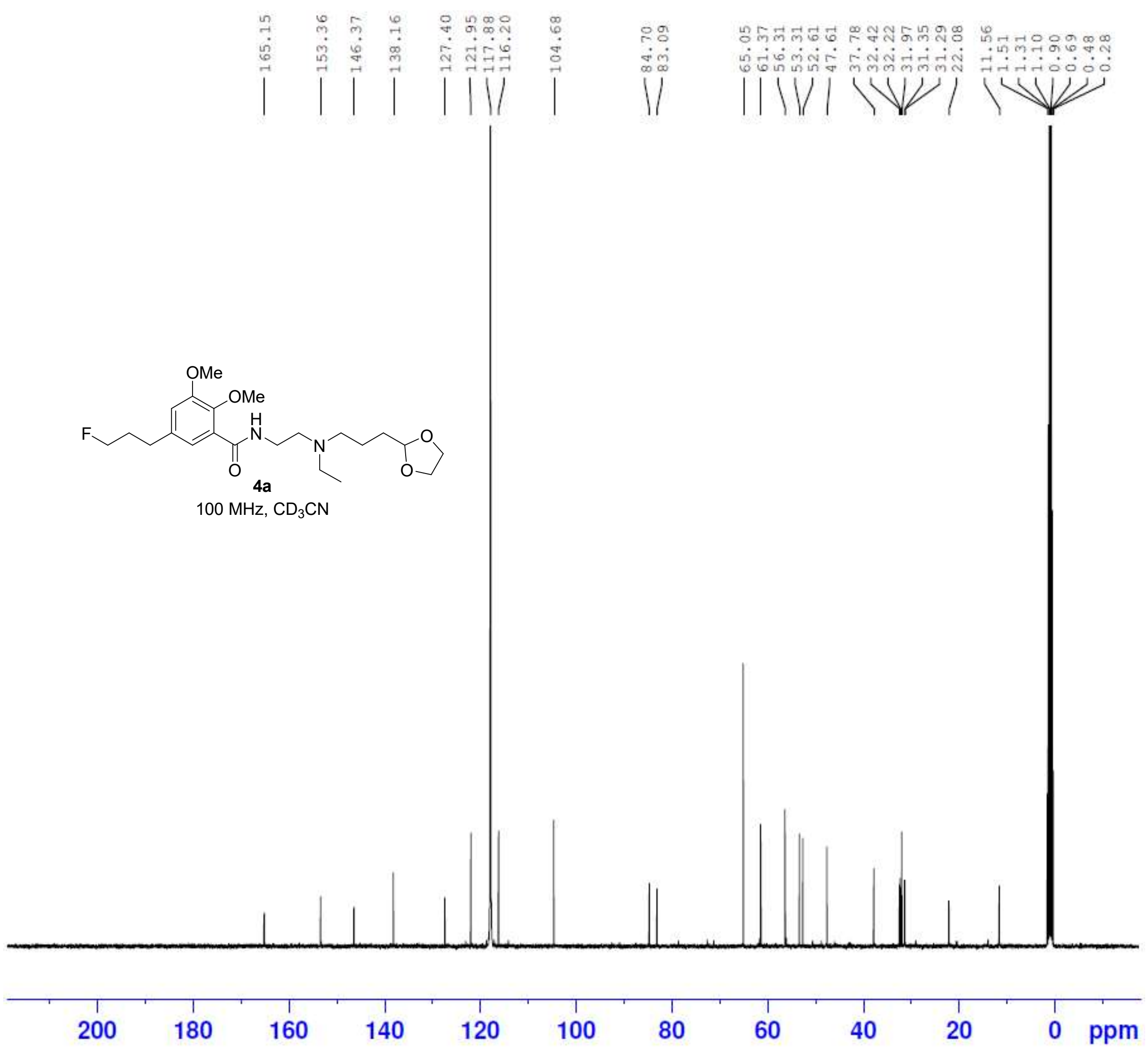
165.15
153.36
146.37
138.16
127.40
121.95
117.88
116.20
104.68
84.70
83.09
65.05
61.37
56.31
53.31
52.61
47.61
37.78
32.42
32.22
31.97
31.35
31.29
22.08
11.56
1.51
1.31
1.10
0.90
0.69
0.48
0.28
OMe
OMe
H
N
F
O
N
O
O
4a
100 MHz, CD3CN
200
180
160
140
120
100
80
60
40
20
0
ppm

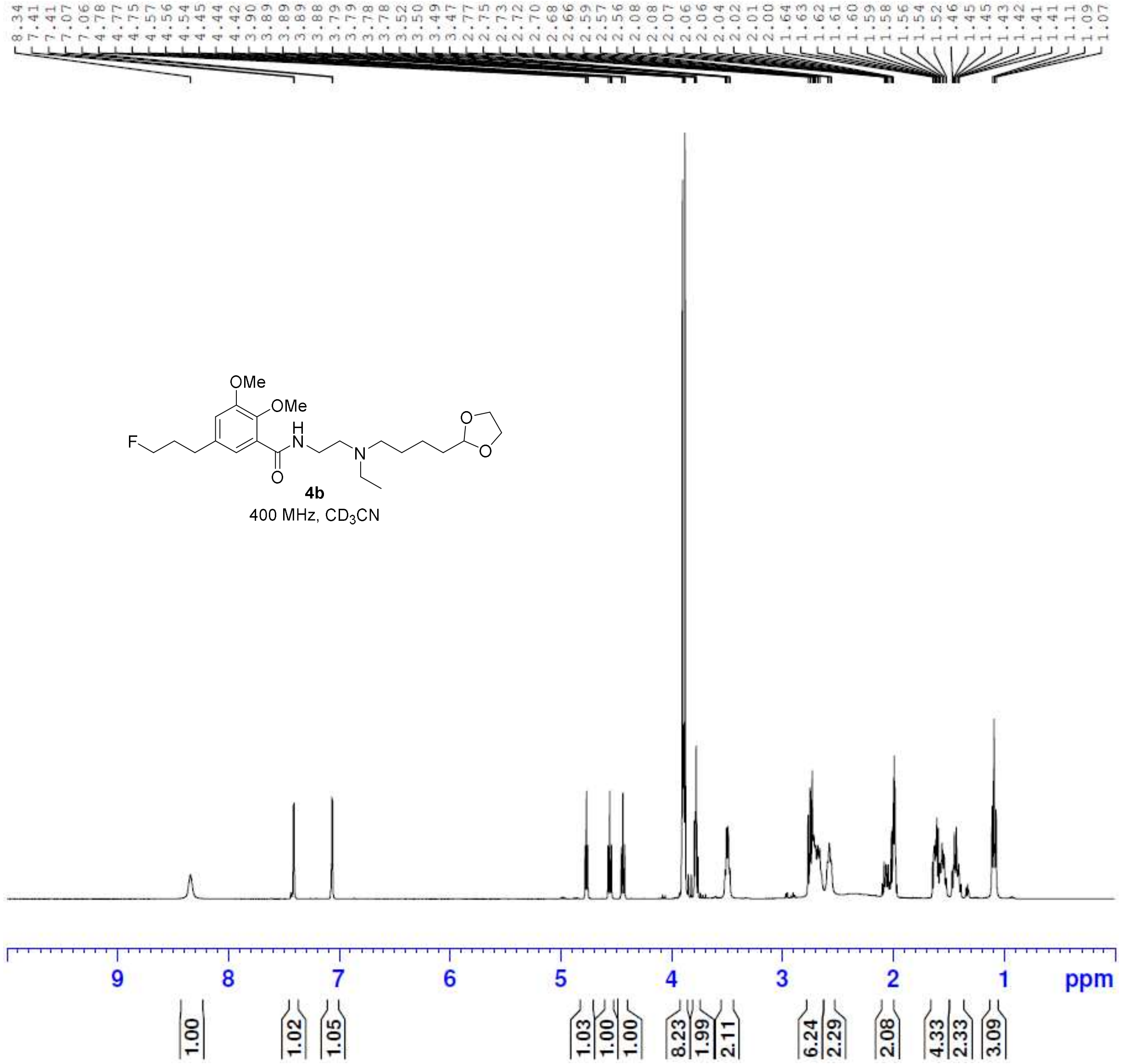

4b

400 MHz, CD_3CN

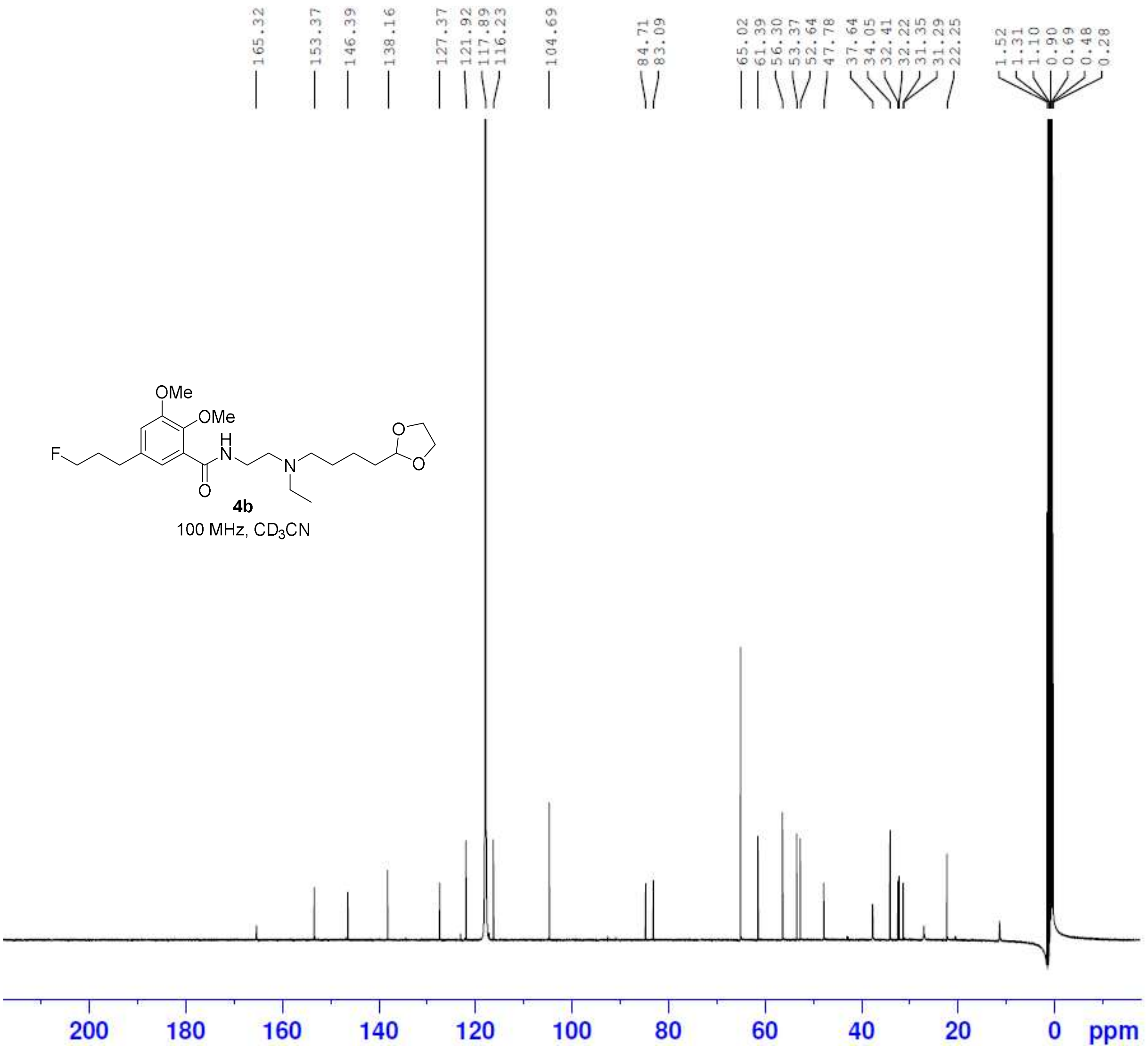

4b

100 MHz, CD_3CN

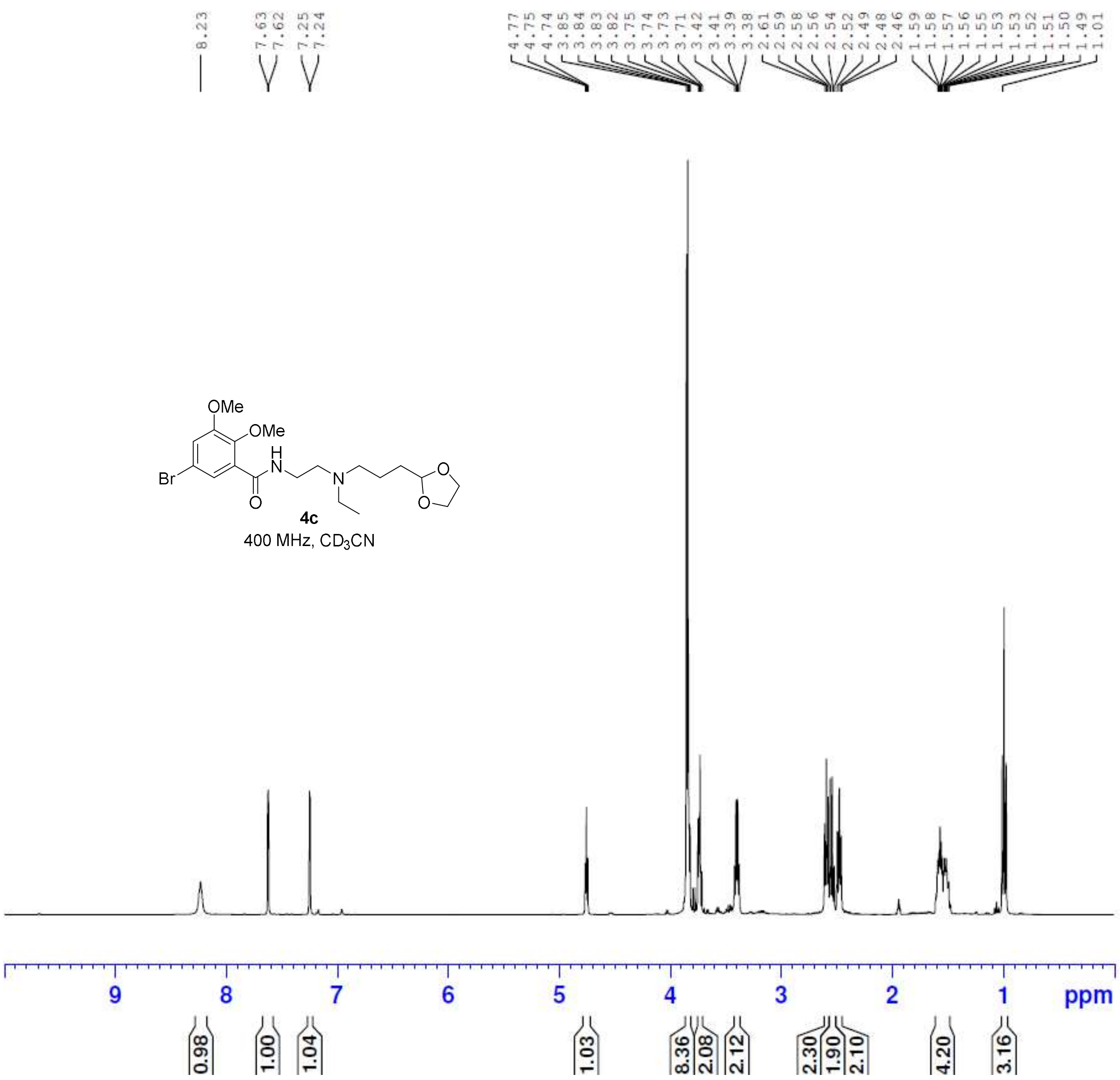
8.23
7.63
7.62
7.25
7.24
4.77
4.75
4.74
3.85
3.84
3.83
3.82
3.75
3.74
3.73
3.71
3.42
3.41
3.39
3.38
2.61
2.59
2.58
2.56
2.54
2.52
2.49
2.48
2.46
1.59
1.58
1.57
1.56
1.55
1.53
1.53
1.52
1.51
1.50
1.49
1.01
OMe
OMe
H
N
Br
O
N
O
O
4c
400 MHz, CD_3CN
9
8
7
6
5
4
3
2
1
ppm
0.98
1.00
1.04
1.03
8.36
2.08
2.12
2.30
1.90
2.10
4.20
3.16

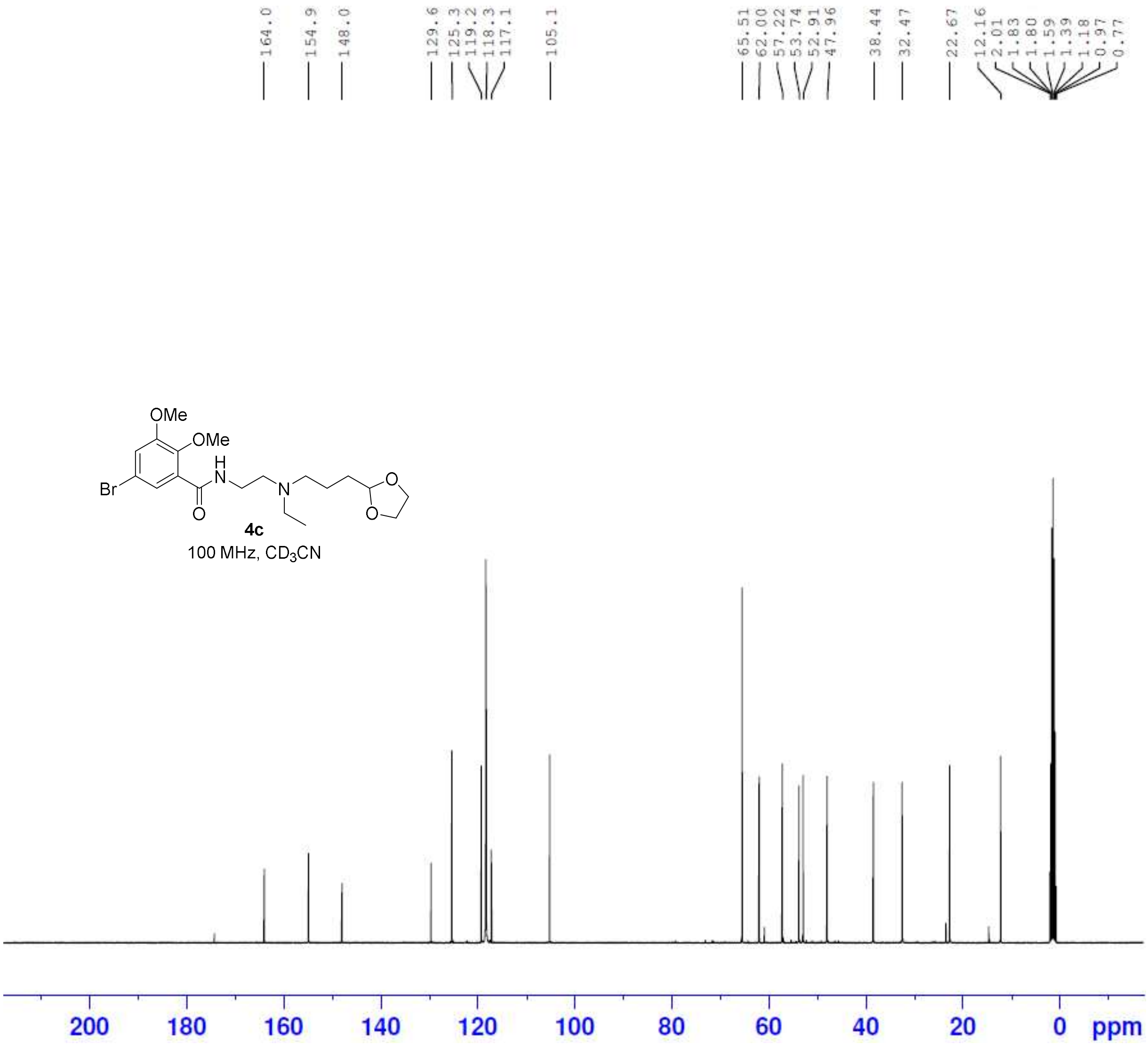
164.0
154.9
148.0
129.6
125.3
119.2
118.3
117.1
105.1
65.51
62.00
57.22
53.74
52.91
47.96
38.44
32.47
22.67
12.16
2.01
1.83
1.80
1.59
1.39
1.18
0.97
0.77
OMe
OMe
H
N
Br
O
N
O
O
4c
100 MHz, CD3CN
200
180
160
140
120
100
80
60
40
20
0
ppm

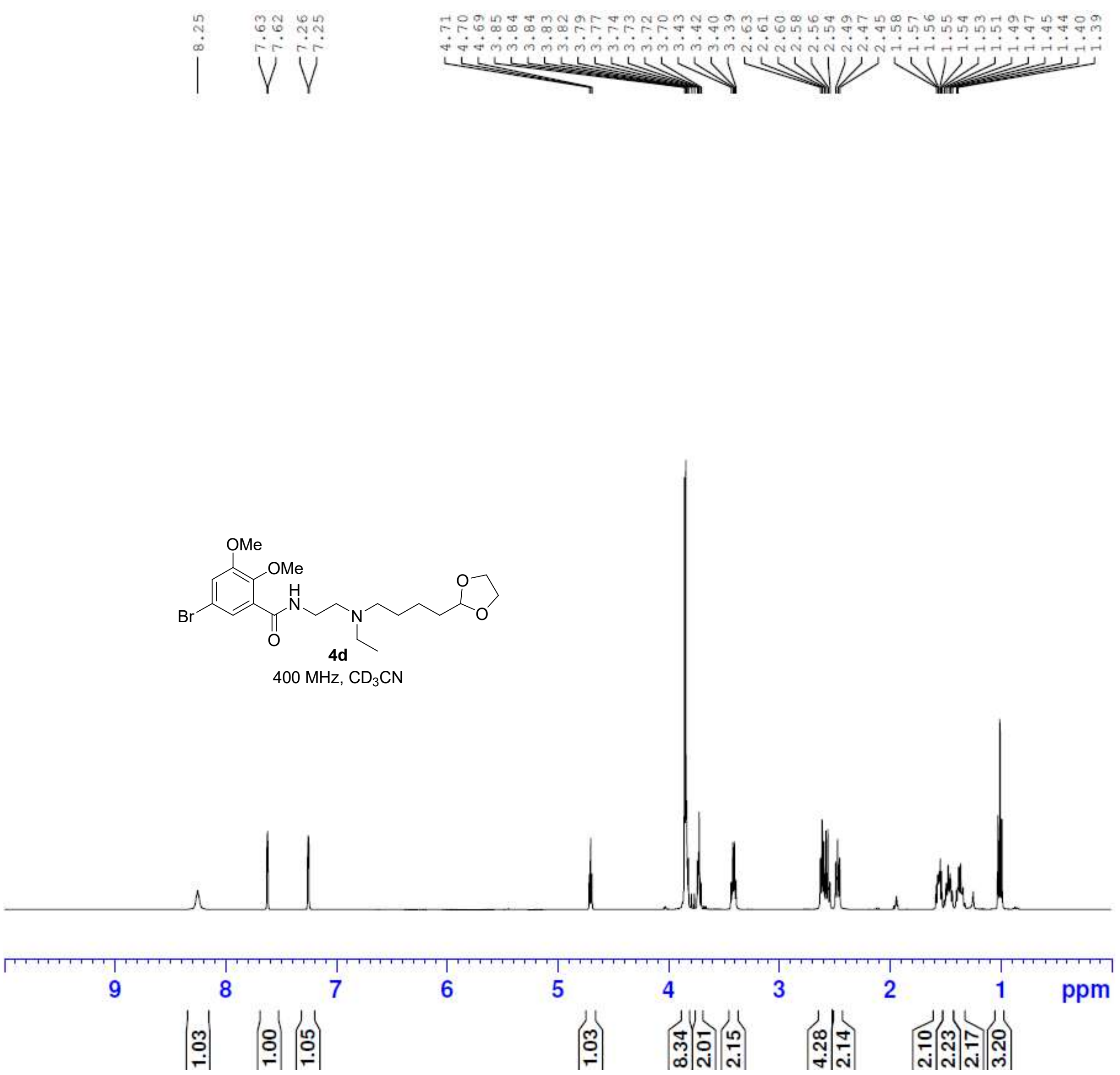
OMe
OMe
Br
H
N
O
N
O
O
4d
400 MHz, CD3CN
ppm

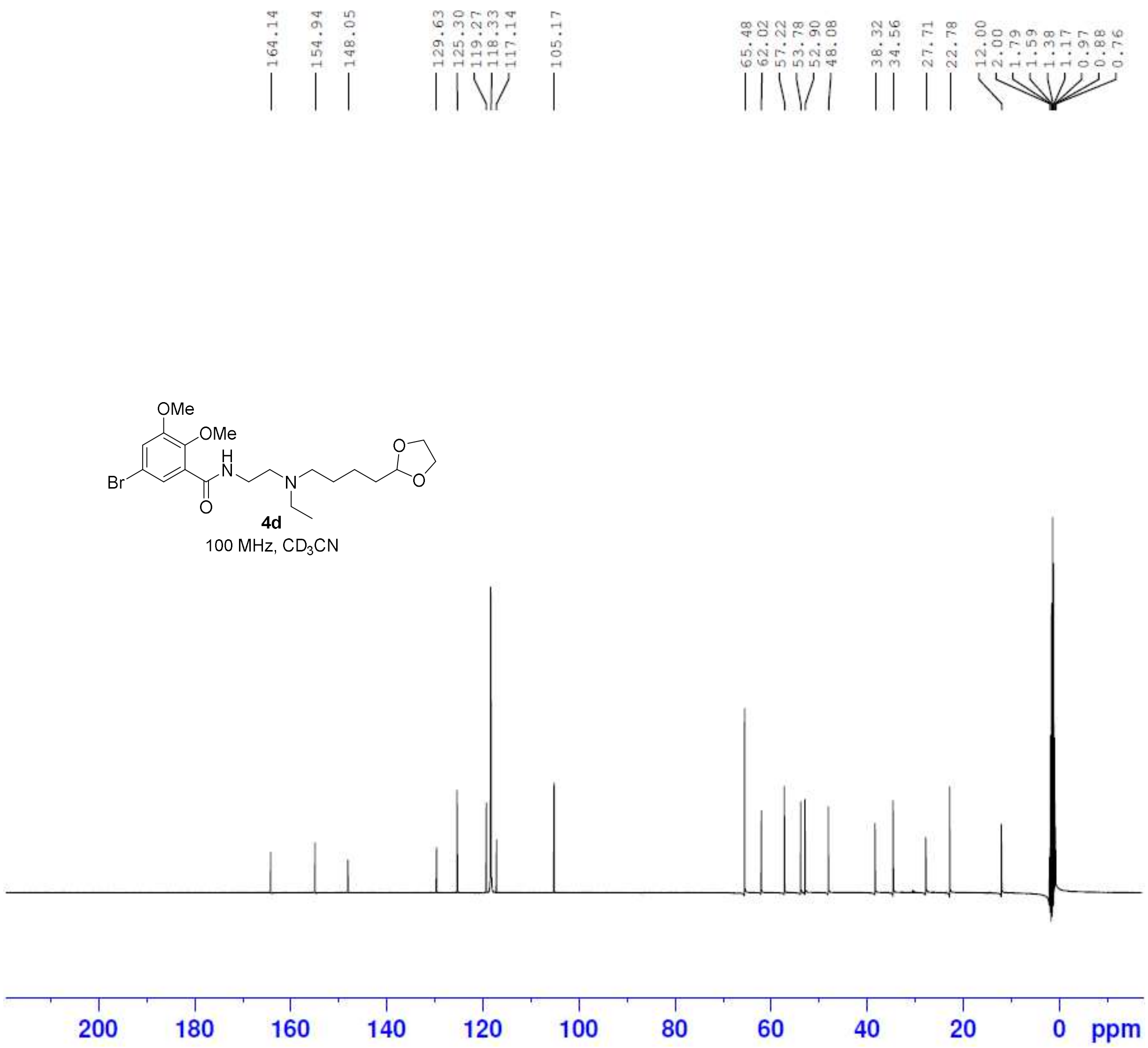

164.14
154.94
148.05
129.63
125.30
119.27
118.33
117.14
105.17
65.48
62.02
57.22
53.78
52.90
48.08
38.32
34.56
27.71
22.78
12.00
2.00
1.79
1.59
1.38
1.17
0.97
0.88
0.76
OMe
OMe
H
N
N
O
O
Br
O
4d
100 MHz, CD3CN
200
180
160
140
120
100
80
60
40
20
0
ppm

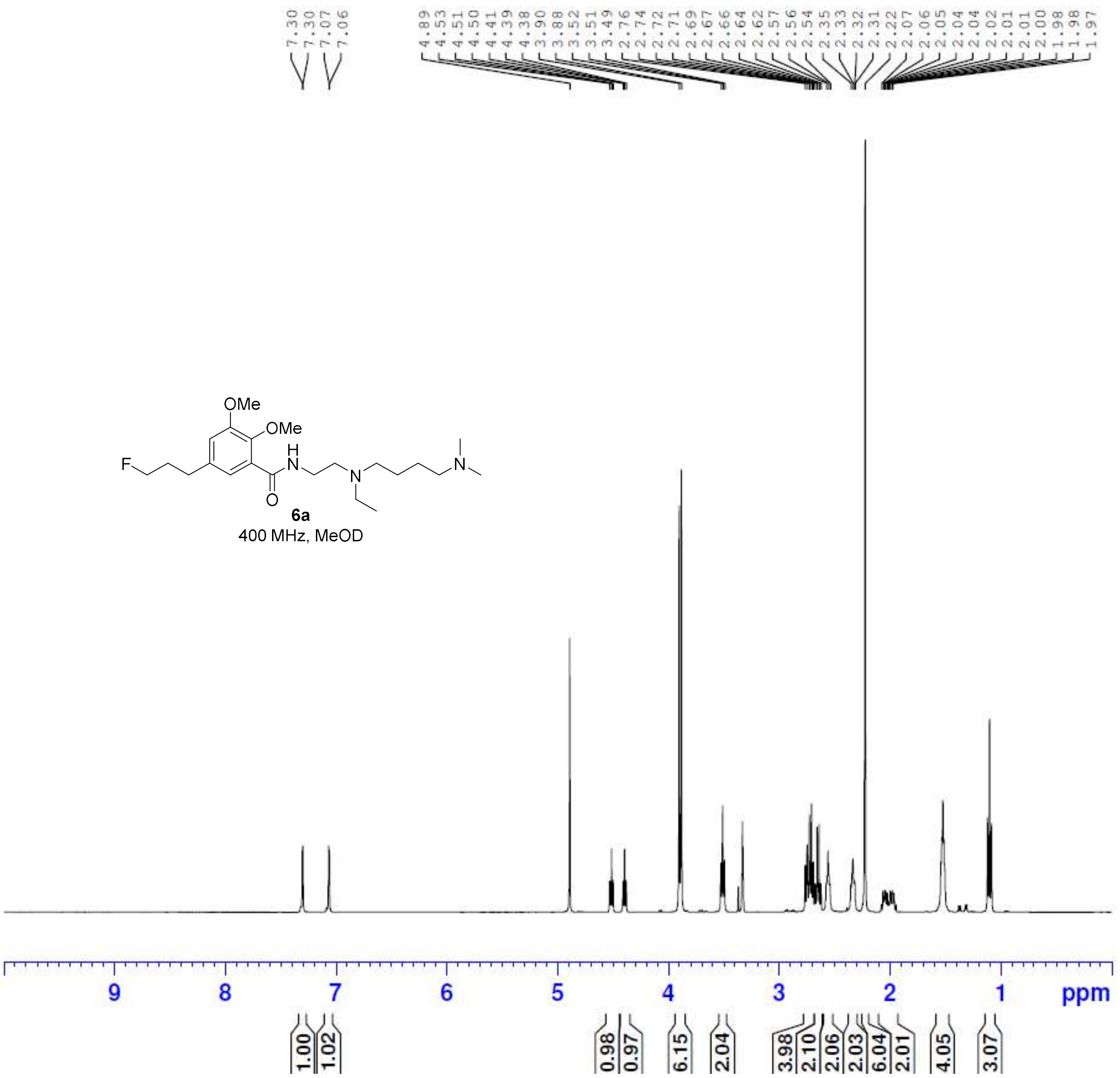
OMe
OMe
H
N
F
N
N
O
6a
400 MHz, MeOD
ppm

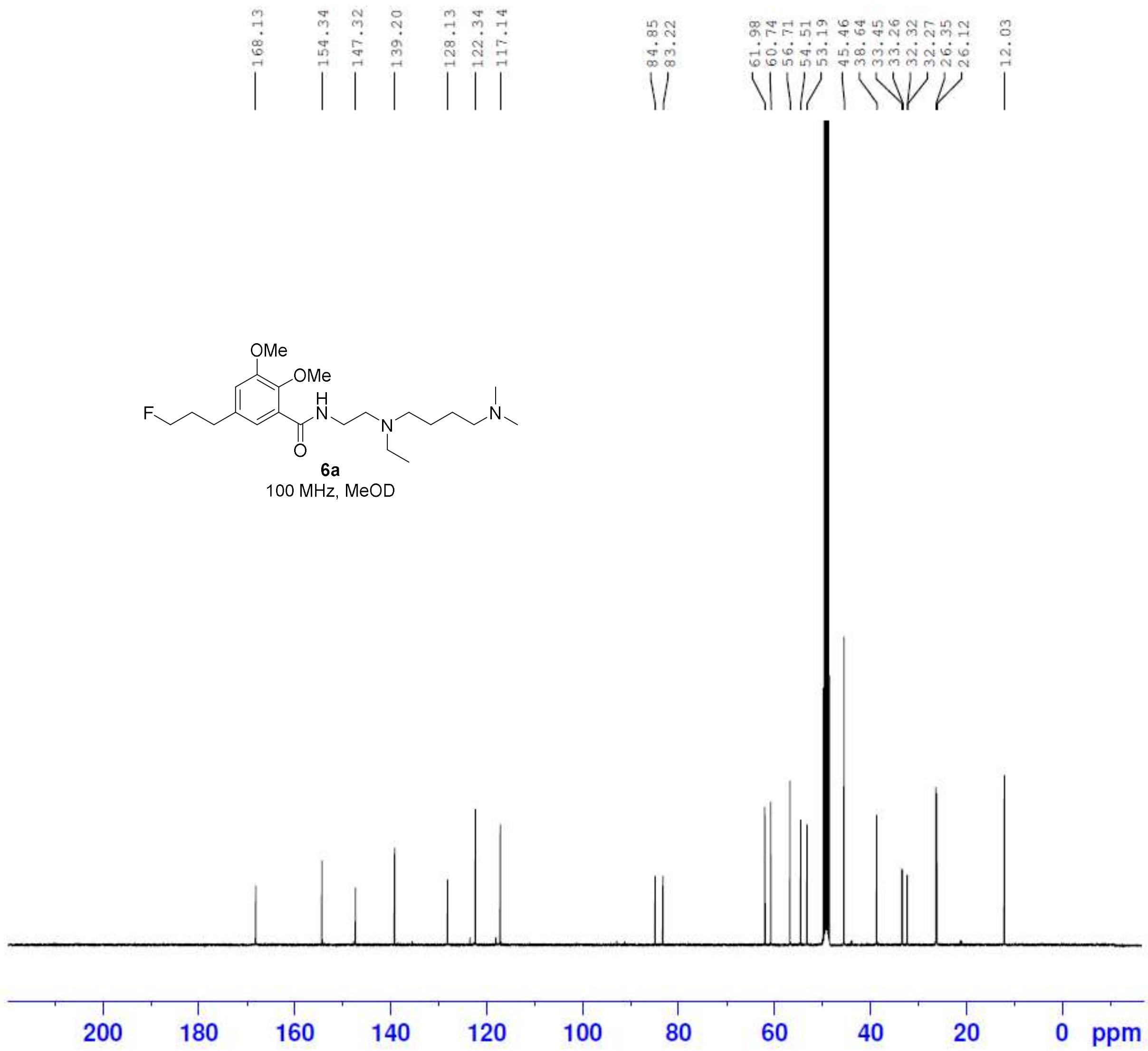
168.13
154.34
147.32
139.20
128.13
122.34
117.14
84.85
83.22
61.98
60.74
56.71
54.51
53.19
45.46
38.64
33.45
33.26
32.32
32.27
26.35
26.12
12.03
OMe
OMe
F
H
N
N
N
O
6a
100 MHz, MeOD
200
180
160
140
120
100
80
60
40
20
0
ppm

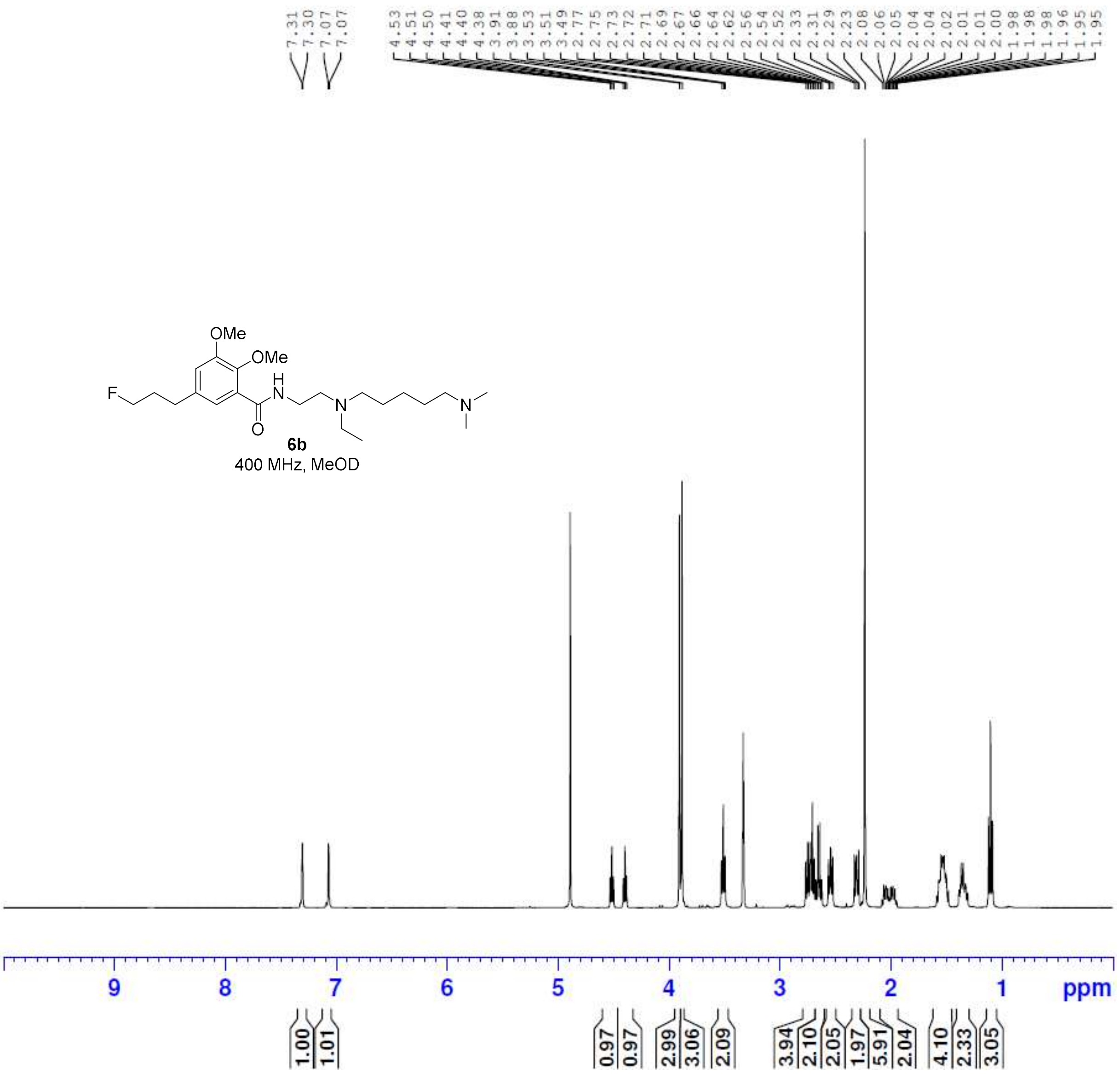
7.31
7.30
7.07
7.07
4.53
4.51
4.50
4.41
4.40
4.38
3.91
3.88
3.53
3.51
3.49
2.77
2.75
2.73
2.72
2.71
2.69
2.67
2.66
2.64
2.62
2.56
2.54
2.52
2.33
2.31
2.29
2.23
2.08
2.06
2.05
2.04
2.04
2.02
2.01
2.01
2.00
1.98
1.98
1.96
1.95
1.95
OMe
OMe
H
N
F
O
N
N
6b
400 MHz, MeOD
9
8
7
6
5
4
3
2
1
ppm
1.00
1.01
0.97
0.97
2.99
3.06
2.09
3.94
2.10
2.05
1.97
5.91
2.04
4.10
2.33
3.05

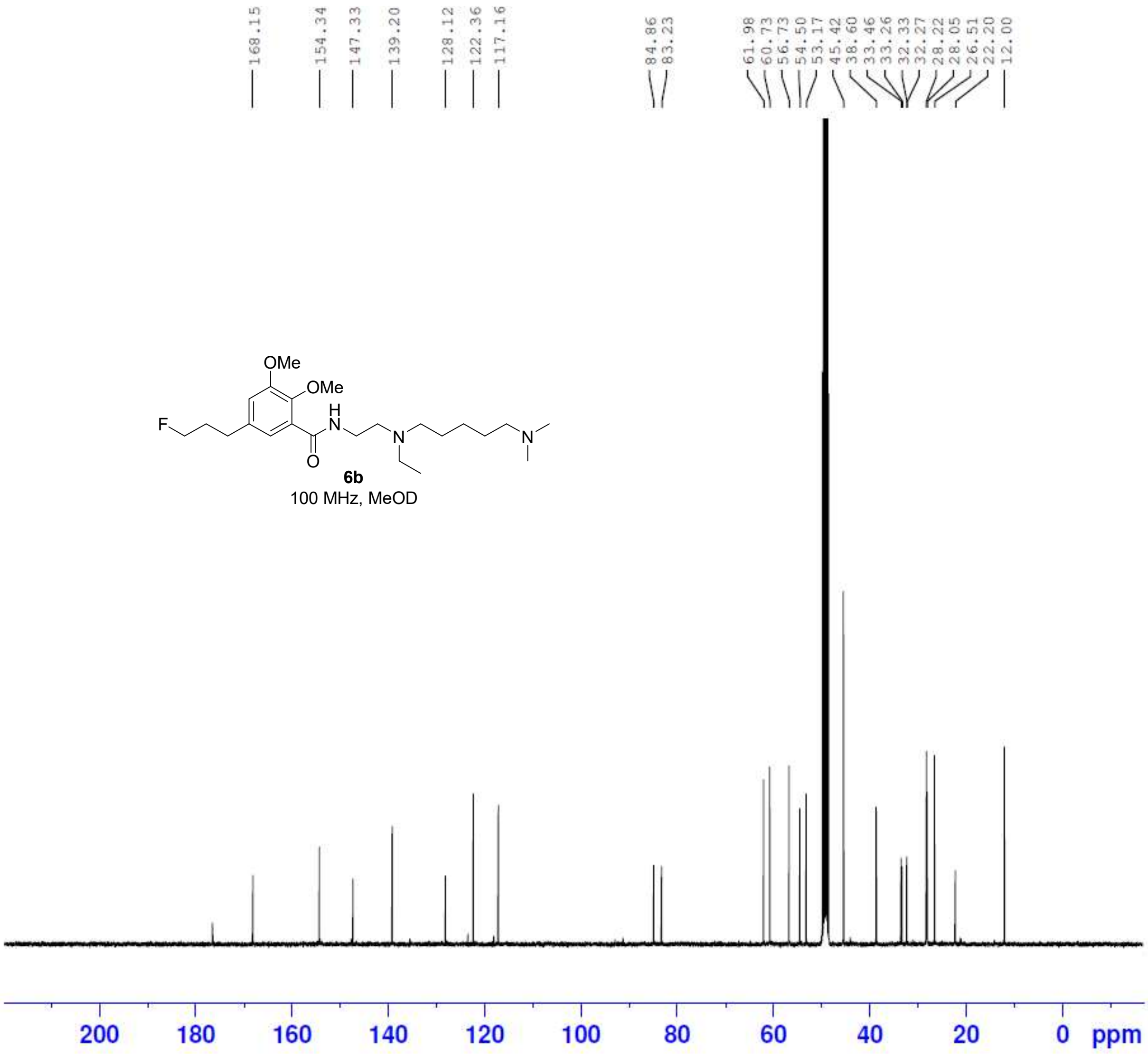

168.15
154.34
147.33
139.20
128.12
122.36
117.16
84.86
83.23
61.98
60.73
56.73
54.50
53.17
45.42
38.60
33.46
33.26
32.33
32.27
28.22
28.05
26.51
22.20
12.00
OMe
OMe
F
H
N
O
N
N
6b
100 MHz, MeOD
200
180
160
140
120
100
80
60
40
20
0
ppm

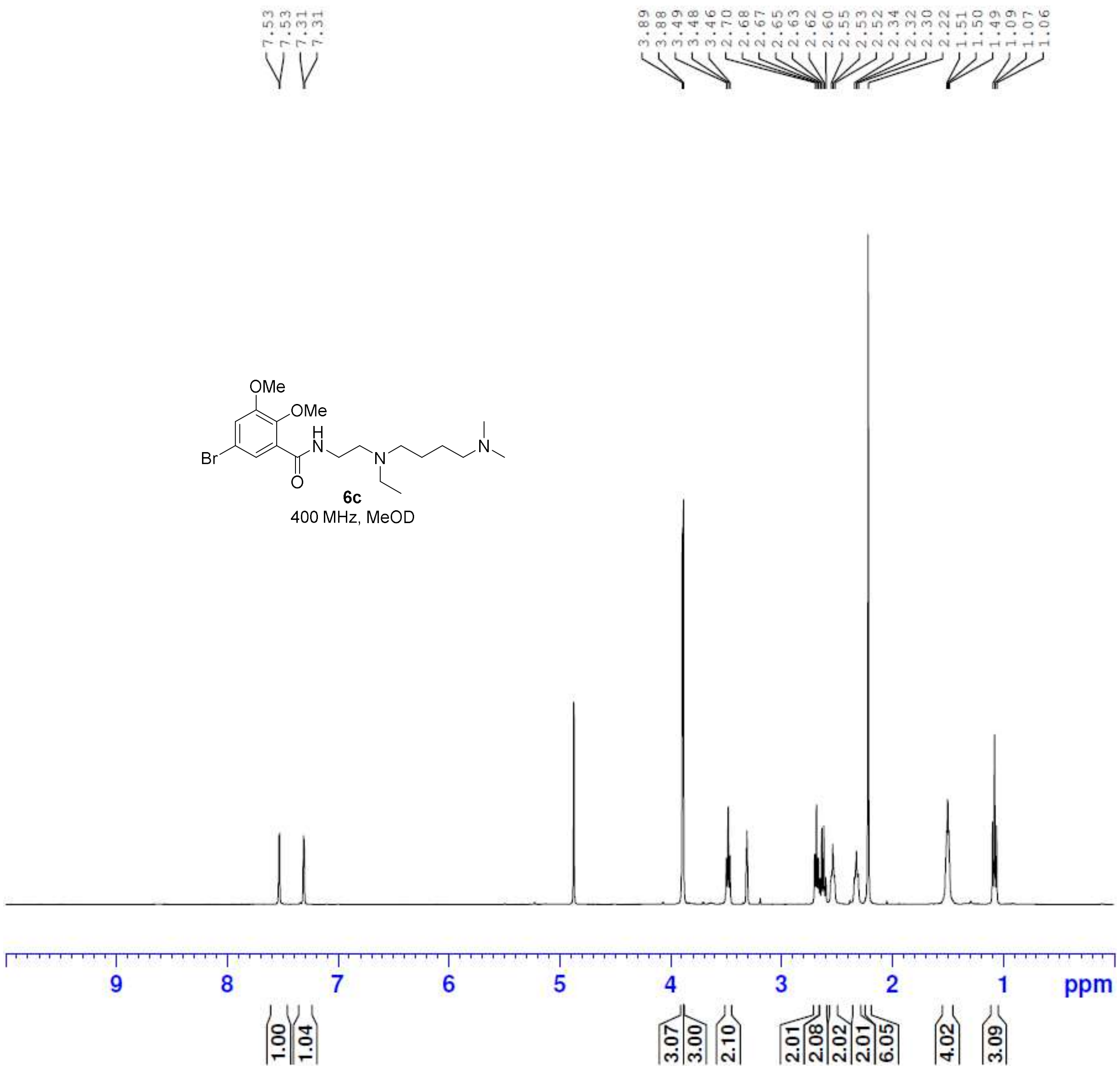

OMe
OMe
H
N
N
N
Br
O
6c
400 MHz, MeOD
7.53
7.53
7.31
7.31
3.89
3.88
3.49
3.48
3.46
2.70
2.68
2.67
2.65
2.63
2.62
2.60
2.55
2.53
2.52
2.34
2.32
2.30
2.22
1.51
1.50
1.49
1.09
1.07
1.06
9
8
7
6
5
4
3
2
1
ppm
1.00
1.04
3.07
3.00
2.10
2.01
2.08
2.02
2.01
6.05
4.02
3.09

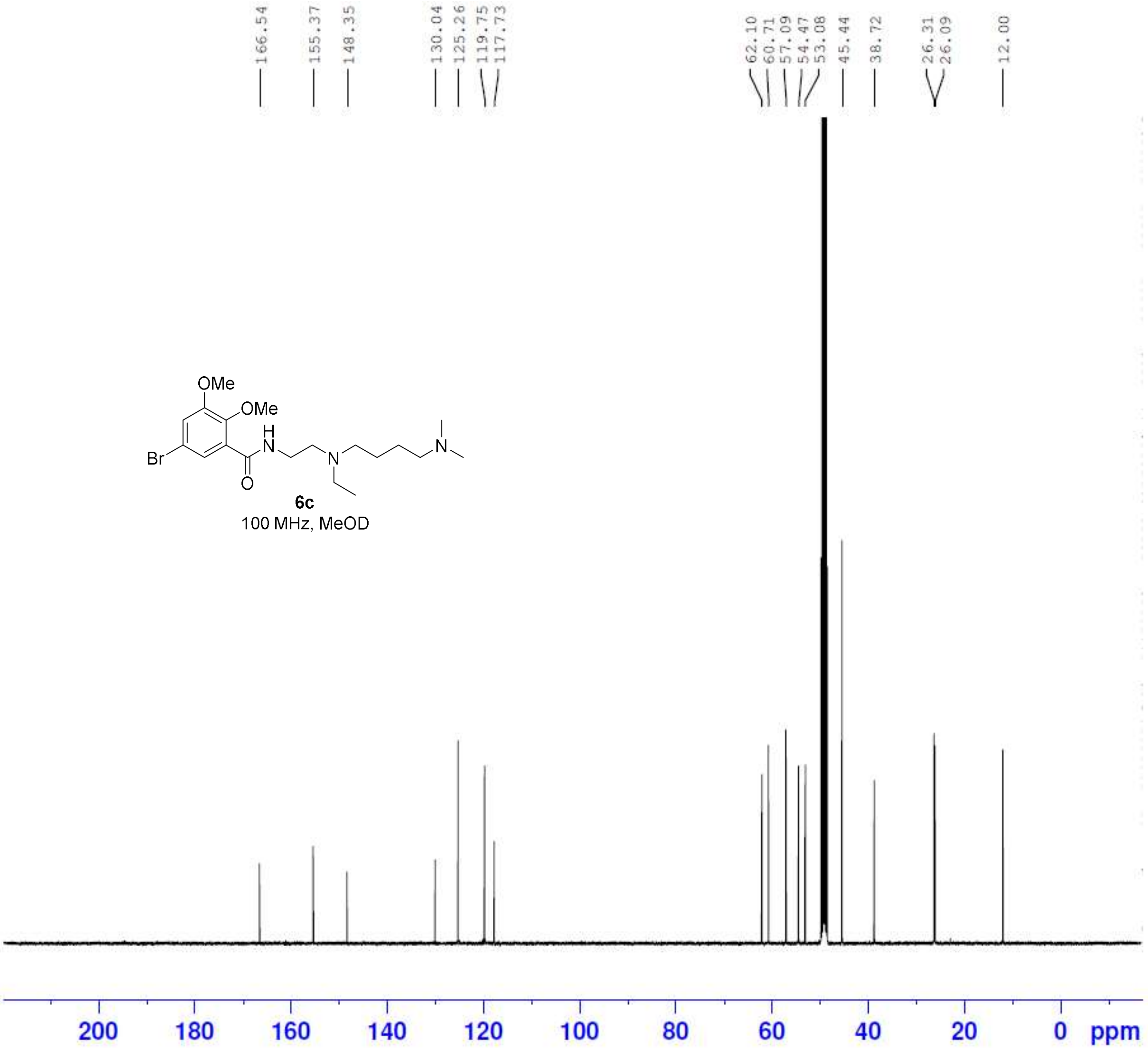
166.54
155.37
148.35
130.04
125.26
119.75
117.73
62.10
60.71
57.09
54.47
53.08
45.44
38.72
26.31
26.09
12.00
OMe
OMe
H
N
N
N
Br
O
6c
100 MHz, MeOD
200
180
160
140
120
100
80
60
40
20
0
ppm

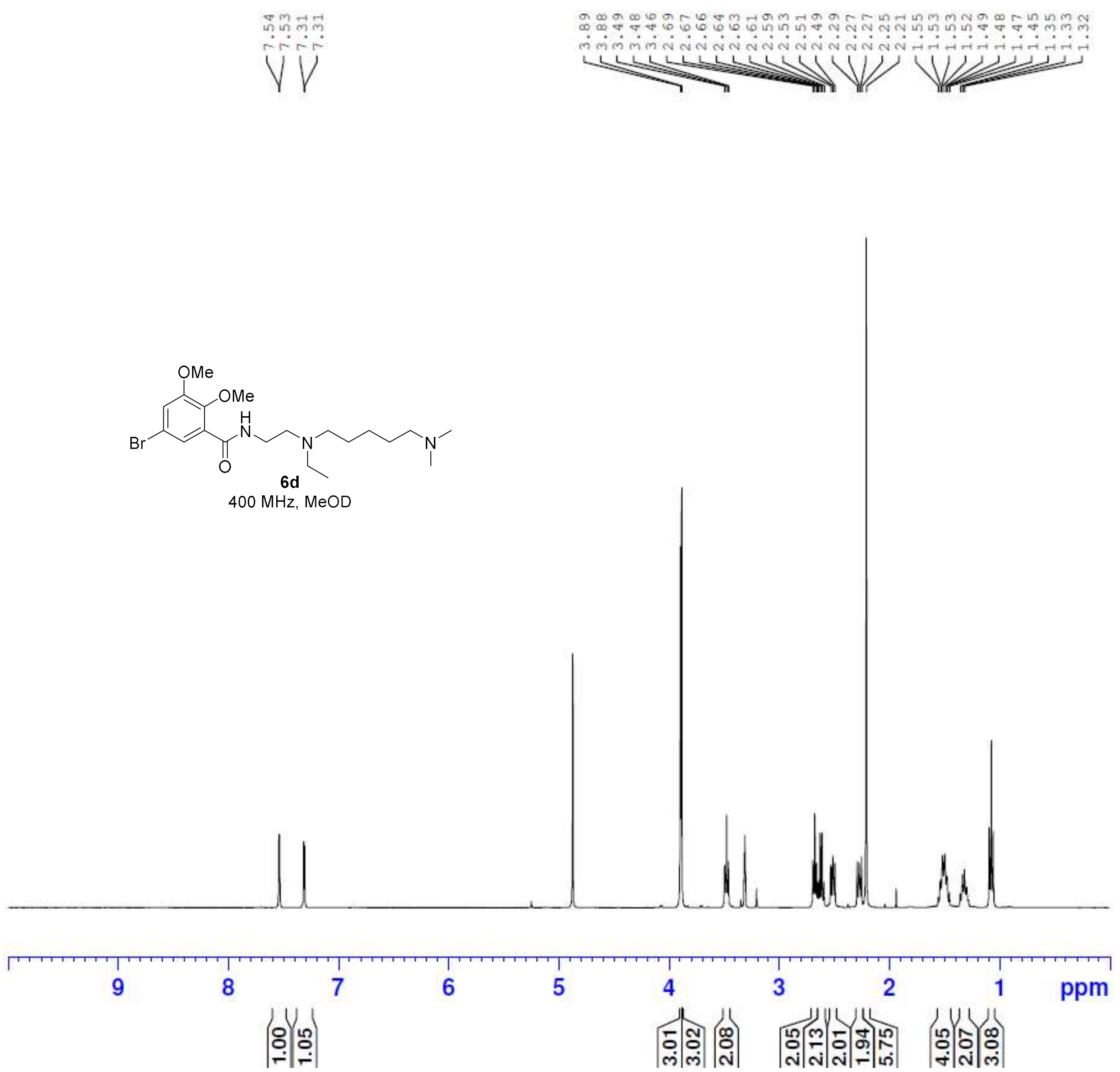
7.54
7.53
7.31
7.31
3.89
3.88
3.49
3.48
3.46
2.69
2.67
2.66
2.64
2.63
2.61
2.59
2.53
2.51
2.49
2.29
2.27
2.27
2.25
2.21
1.55
1.53
1.53
1.52
1.49
1.48
1.47
1.45
1.35
1.33
1.32
OMe
OMe
H
N
Br
O
N
N
6d
400 MHz, MeOD
9
8
7
6
5
4
3
2
1
ppm
1.00
1.05
3.01
3.02
2.08
2.05
2.13
2.01
1.94
5.75
4.05
2.07
3.08

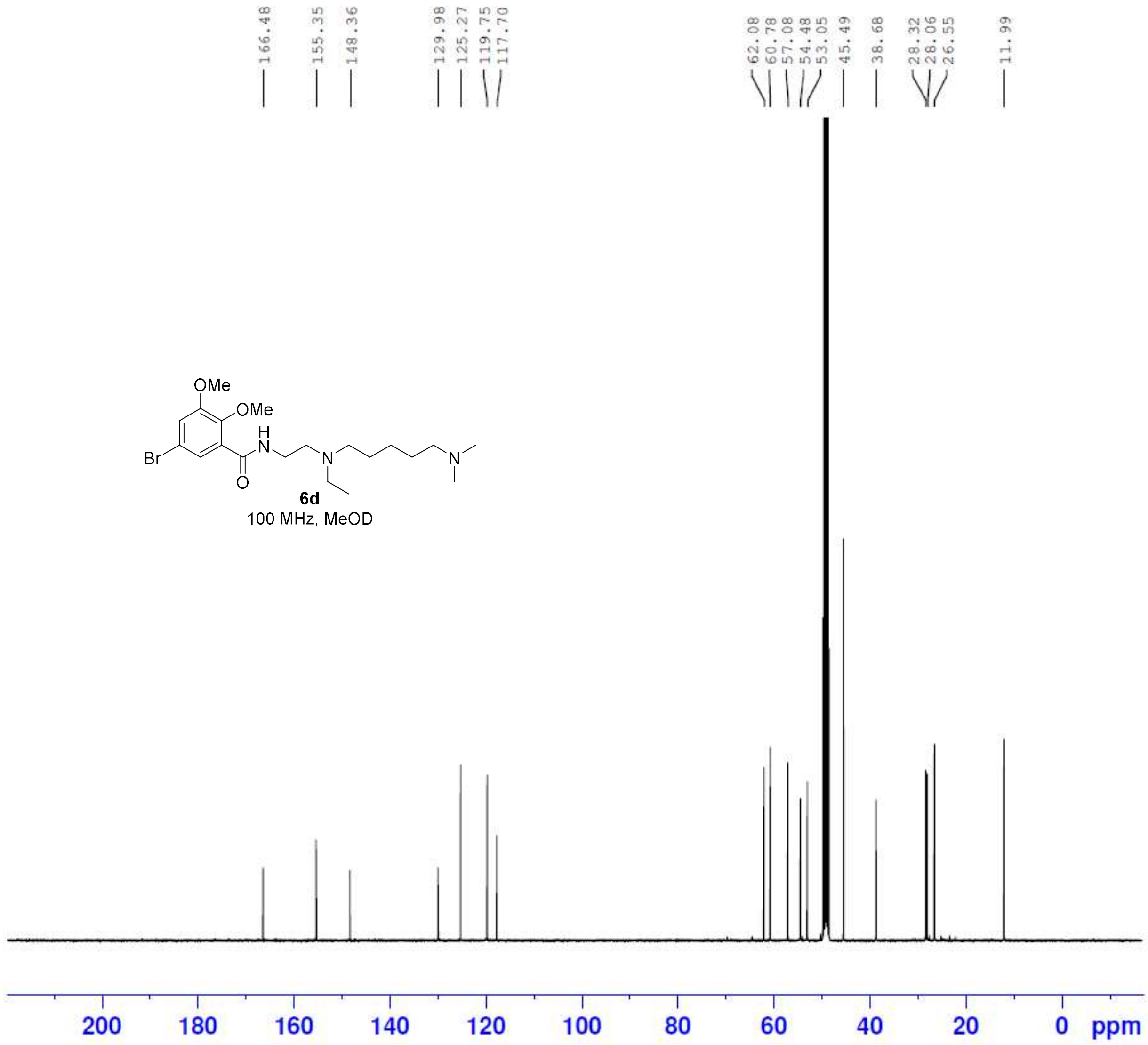
166.48
155.35
148.36
129.98
125.27
119.75
117.70
62.08
60.78
57.08
54.48
53.05
45.49
38.68
28.32
28.06
26.55
11.99
OMe
OMe
H
N
Br
O
N
N
6d
100 MHz, MeOD
200
180
160
140
120
100
80
60
40
20
0
ppm

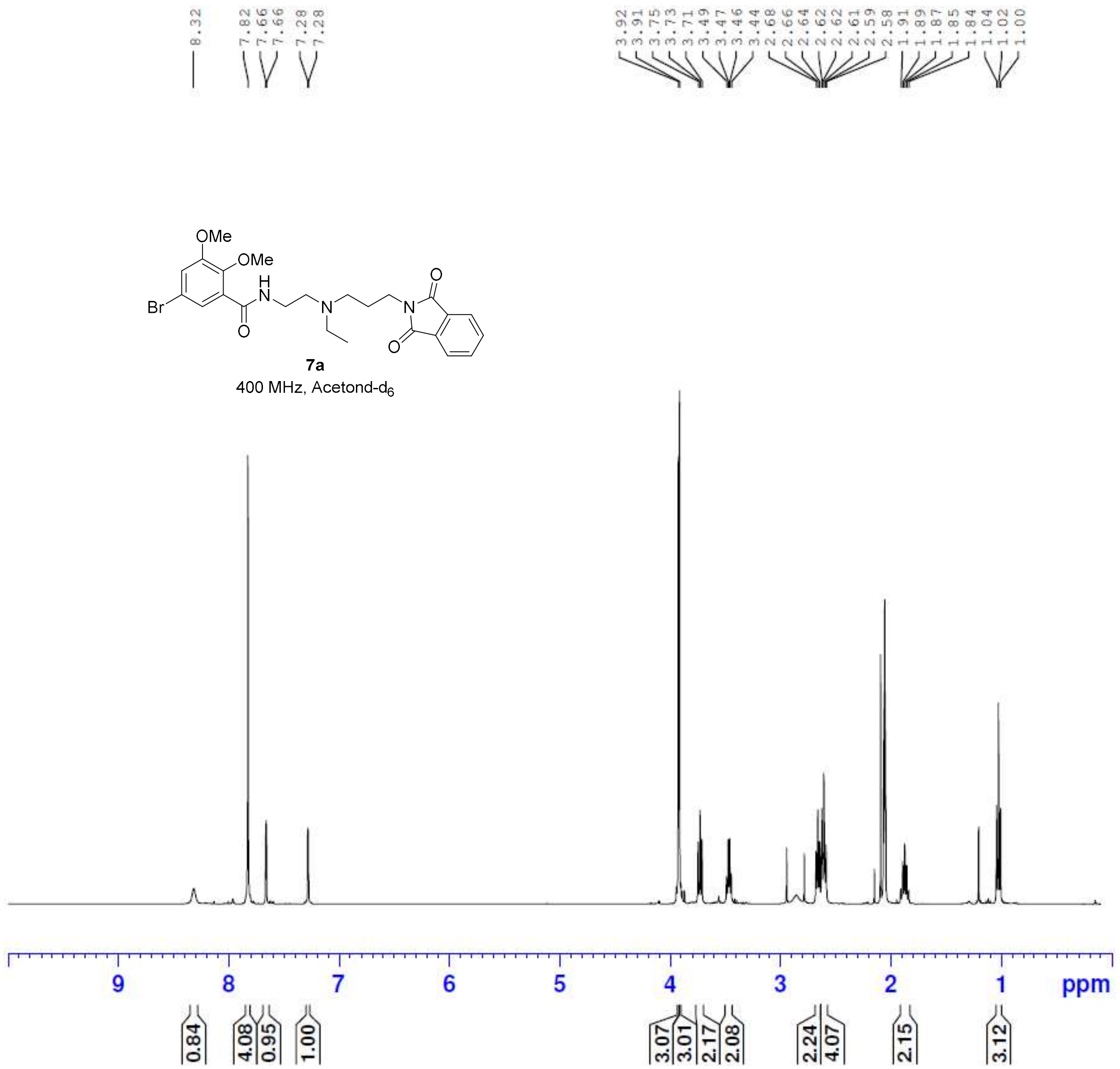

7a

400 MHz, Acetond-d$_6$

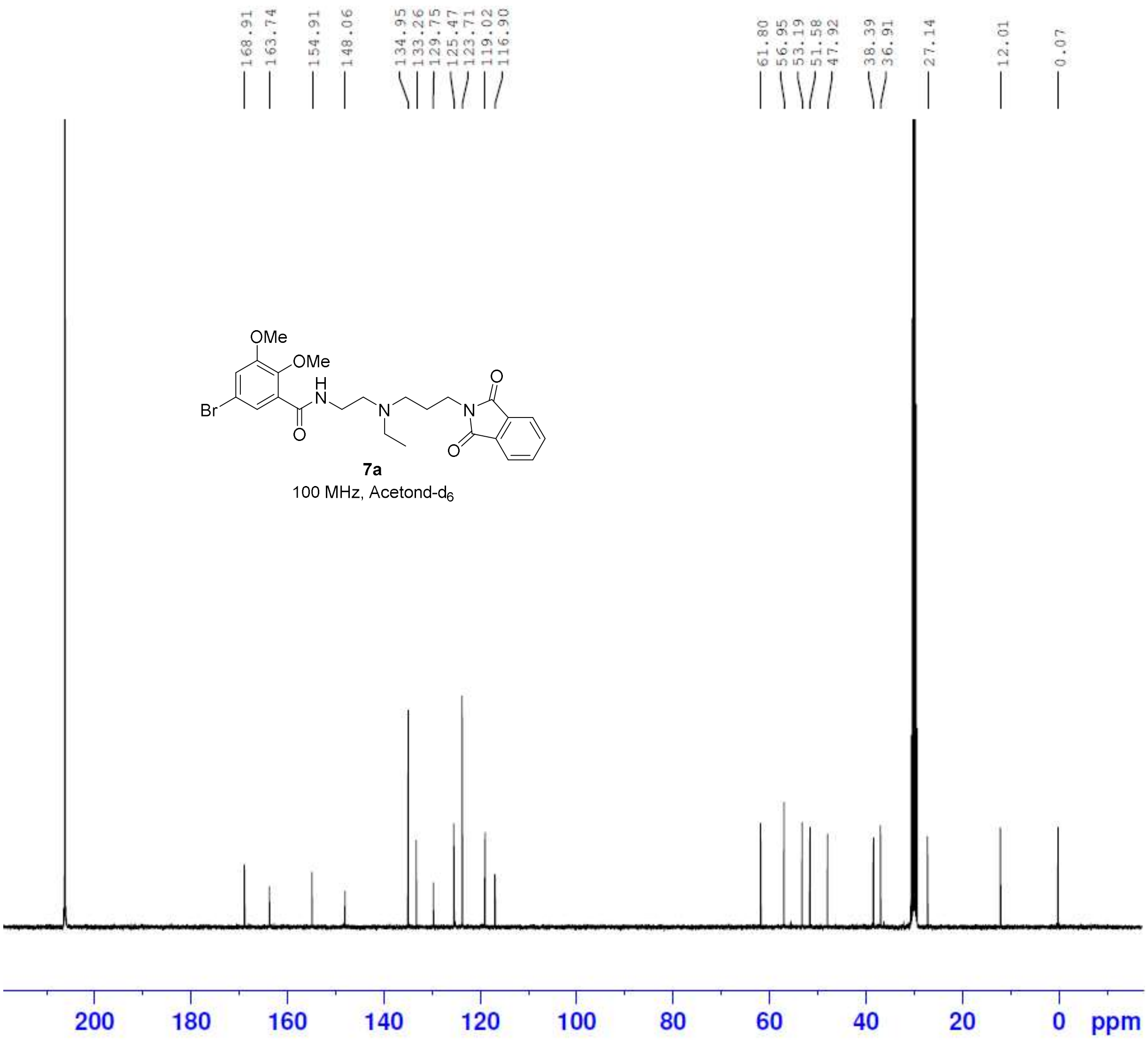
168.91
163.74
154.91
148.06
134.95
133.26
129.75
125.47
123.71
119.02
116.90
61.80
56.95
53.19
51.58
47.92
38.39
36.91
27.14
12.01
0.07
OMe
OMe
H
N
Br
O
N
O
N
O
7a
100 MHz, Acetond-d6
200
180
160
140
120
100
80
60
40
20
0
ppm

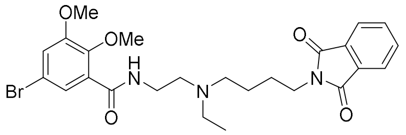

7b

400 MHz, Acetond-d_6

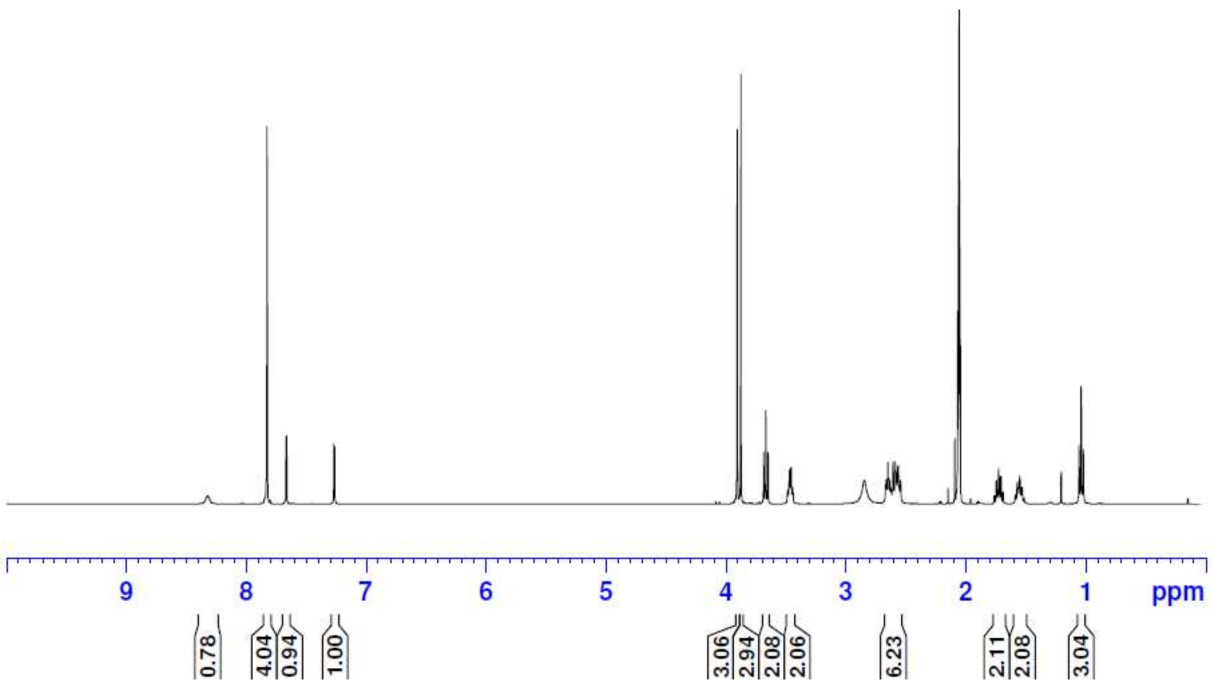

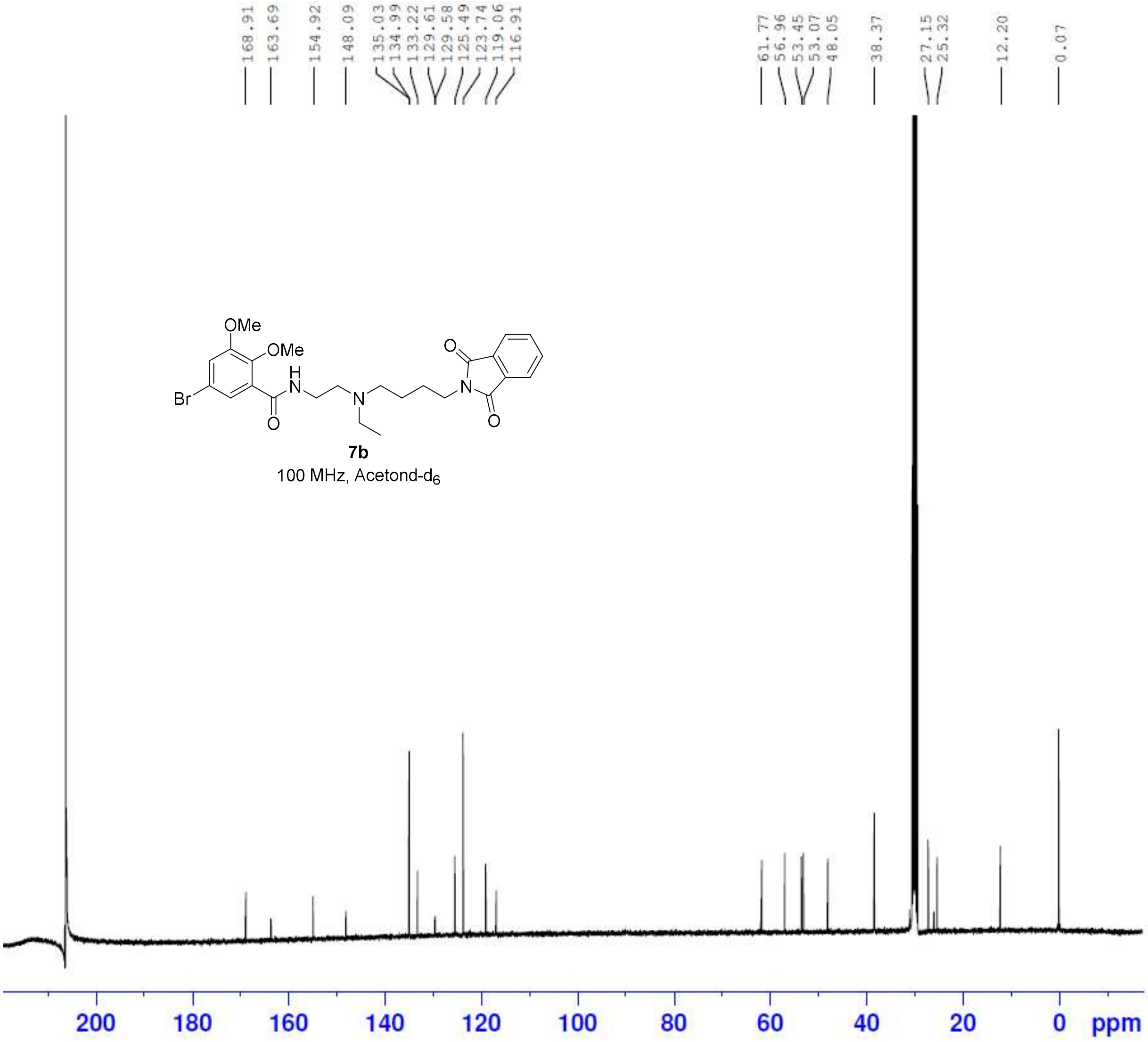
168.91
163.69
154.92
148.09
135.03
134.99
133.22
129.61
129.58
125.49
123.74
119.06
116.91
61.77
56.96
53.45
53.07
48.05
38.37
27.15
25.32
12.20
0.07
OMe
OMe
H
N
Br
O
N
O
N
O
7b
100 MHz, Acetond-d6
200
180
160
140
120
100
80
60
40
20
0
ppm

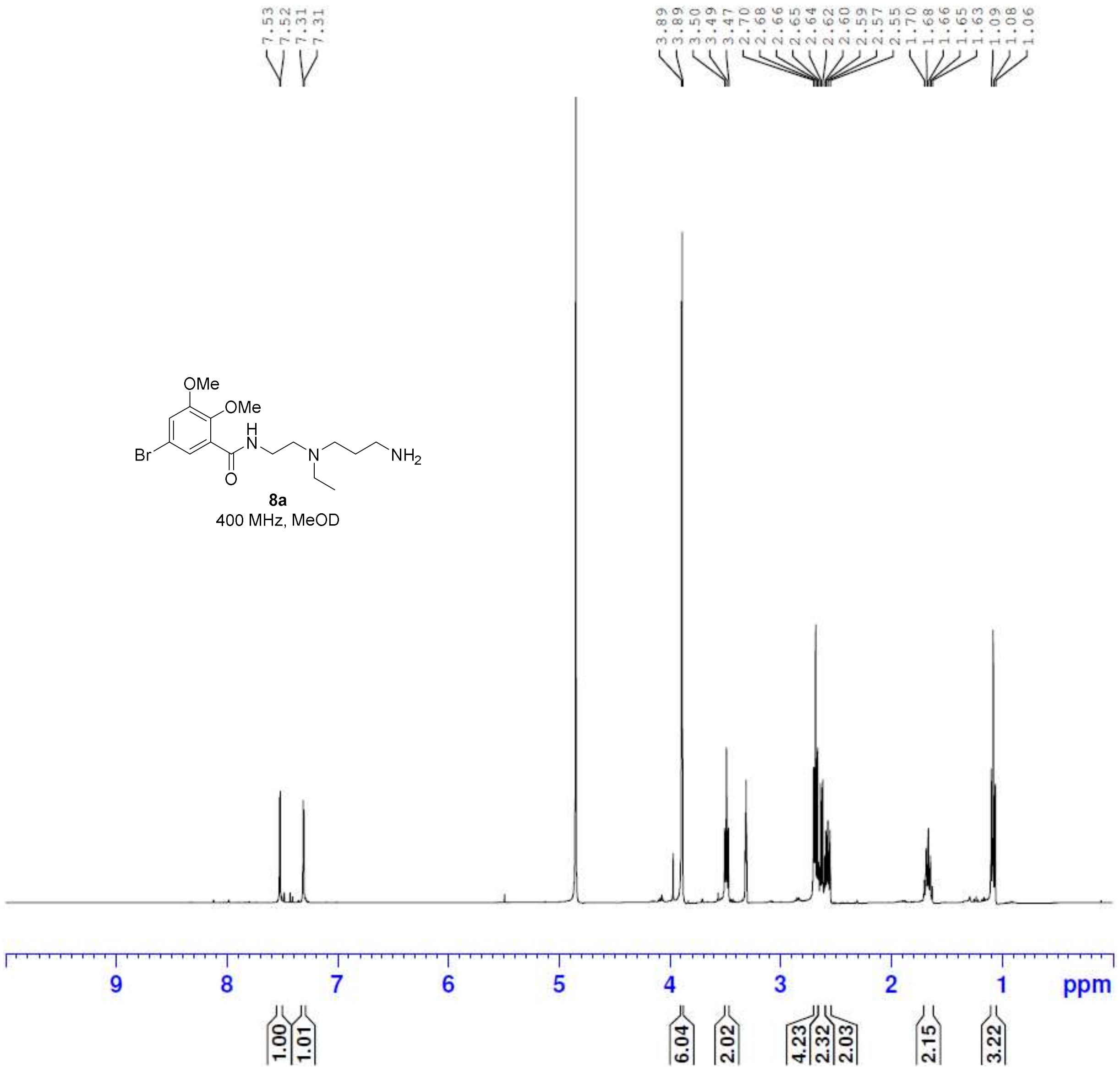
7.53
7.52
7.31
7.31
3.89
3.89
3.50
3.49
3.47
2.70
2.68
2.66
2.65
2.64
2.62
2.60
2.59
2.57
2.55
1.70
1.68
1.66
1.65
1.63
1.09
1.08
1.06
OMe
OMe
H
N
Br
O
N
NH2
8a
400 MHz, MeOD
9
8
7
6
5
4
3
2
1
ppm
1.00
1.01
6.04
2.02
4.23
2.32
2.32
2.03
2.15
3.22

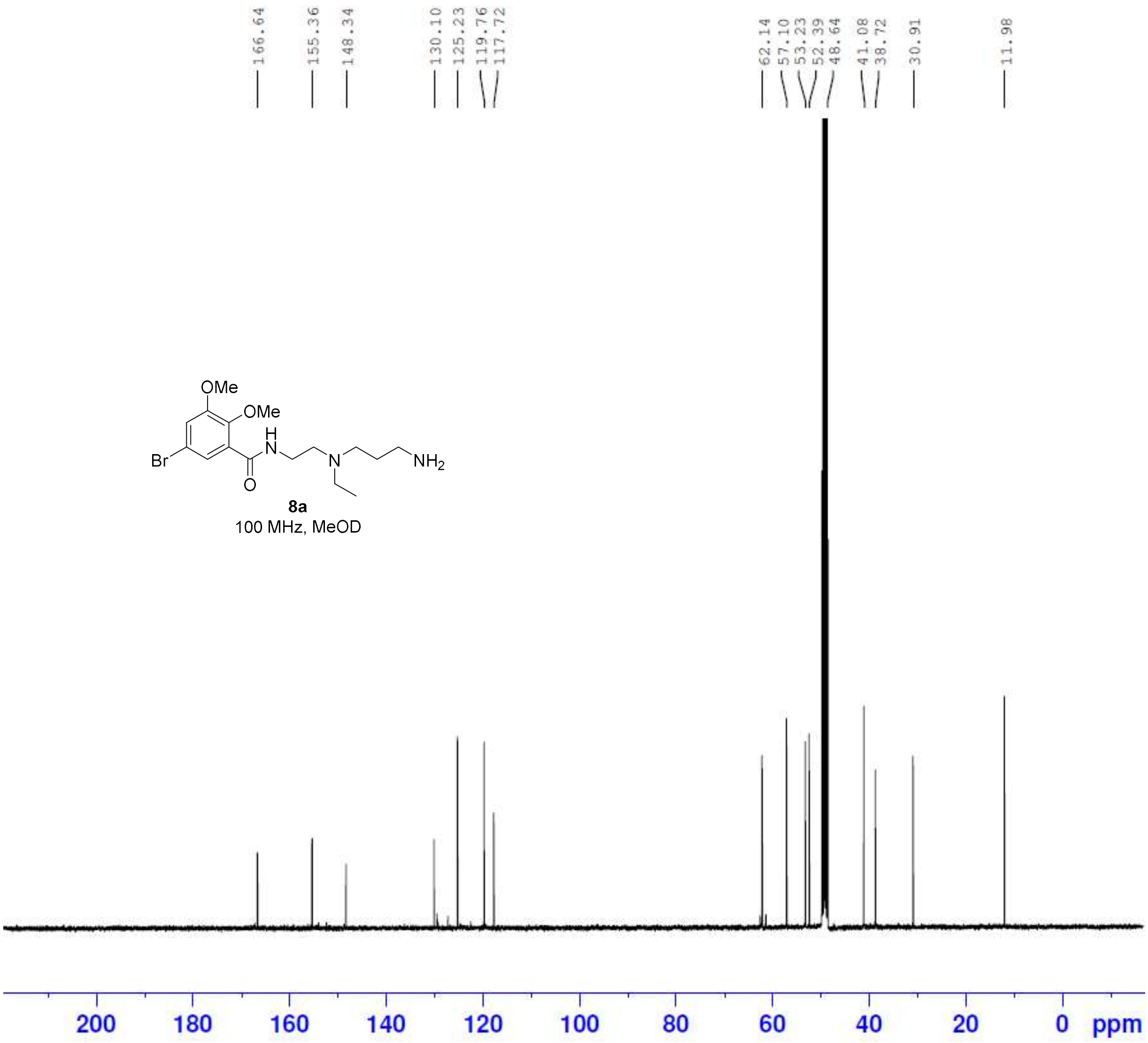
166.64
155.36
148.34
130.10
125.23
119.76
117.72
62.14
57.10
53.23
52.39
48.64
41.08
38.72
30.91
11.98
OMe
OMe
H
N
Br
N
NH2
O
8a
100 MHz, MeOD
200
180
160
140
120
100
80
60
40
20
0
ppm

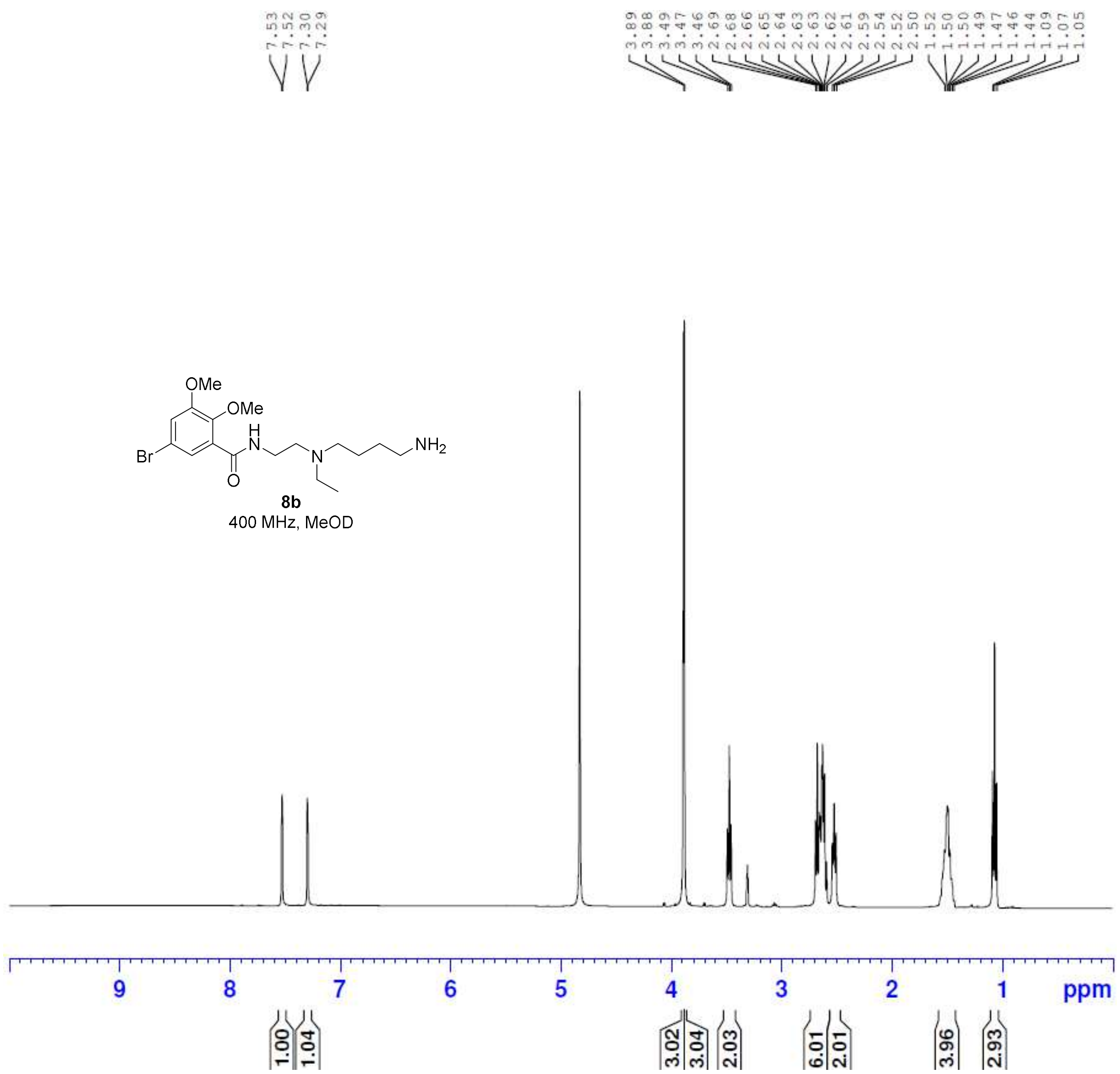
7.53
7.52
7.30
7.29
3.89
3.88
3.49
3.47
3.46
2.69
2.68
2.66
2.65
2.64
2.63
2.63
2.62
2.61
2.59
2.54
2.52
2.50
1.52
1.50
1.50
1.49
1.47
1.46
1.44
1.09
1.07
1.05
OMe
OMe
H
N
Br
O
N
NH2
8b
400 MHz, MeOD
9
8
7
6
5
4
3
2
1
ppm
1.00
1.04
3.02
3.04
2.03
6.01
2.01
3.96
2.93

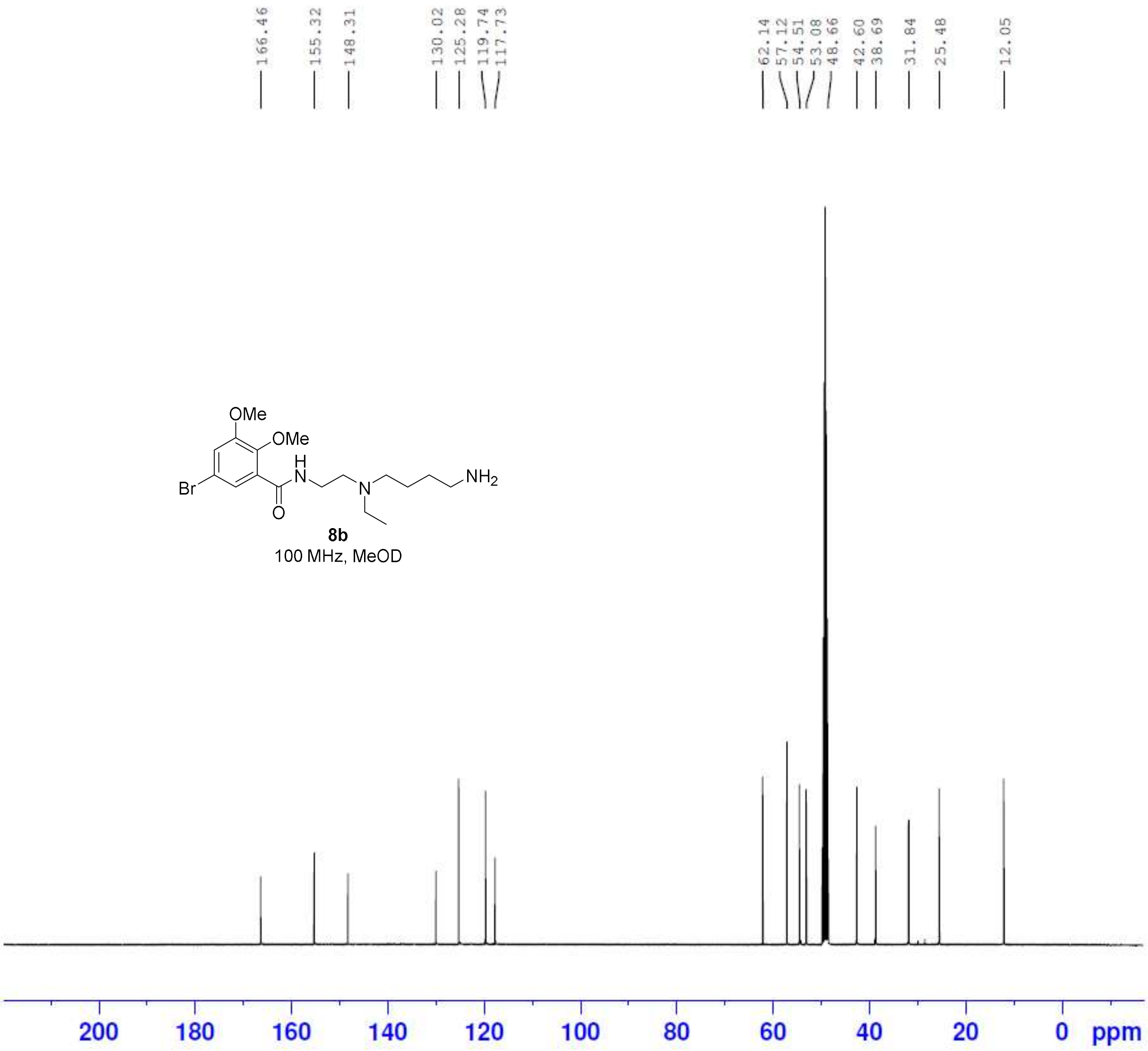
166.46
155.32
148.31
130.02
125.28
119.74
117.73
62.14
57.12
54.51
53.08
48.66
42.60
38.69
31.84
25.48
12.05
OMe
OMe
H
N
Br
O
N
NH2
8b
100 MHz, MeOD
200
180
160
140
120
100
80
60
40
20
0
ppm

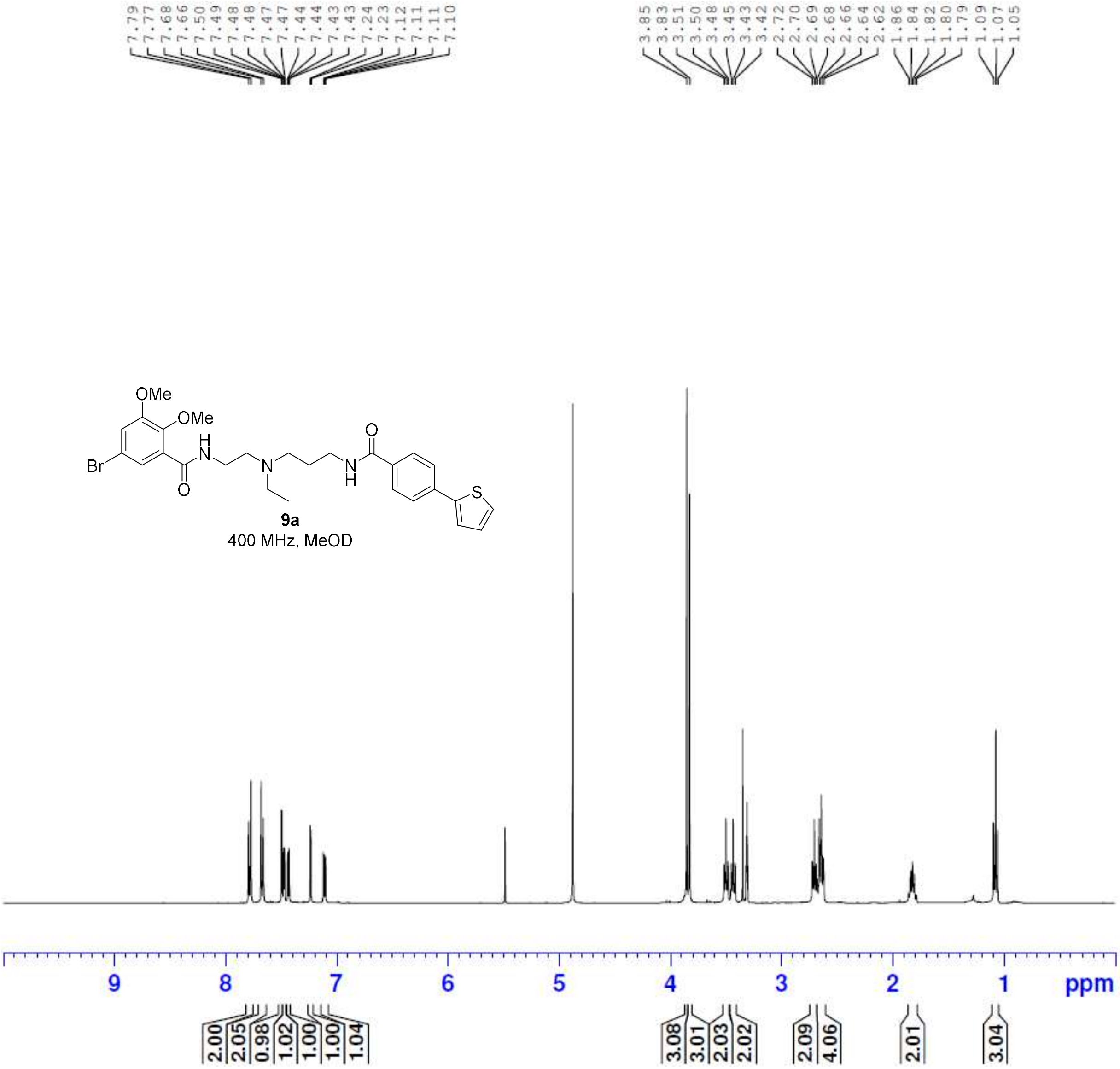

9a
400 MHz, MeOD

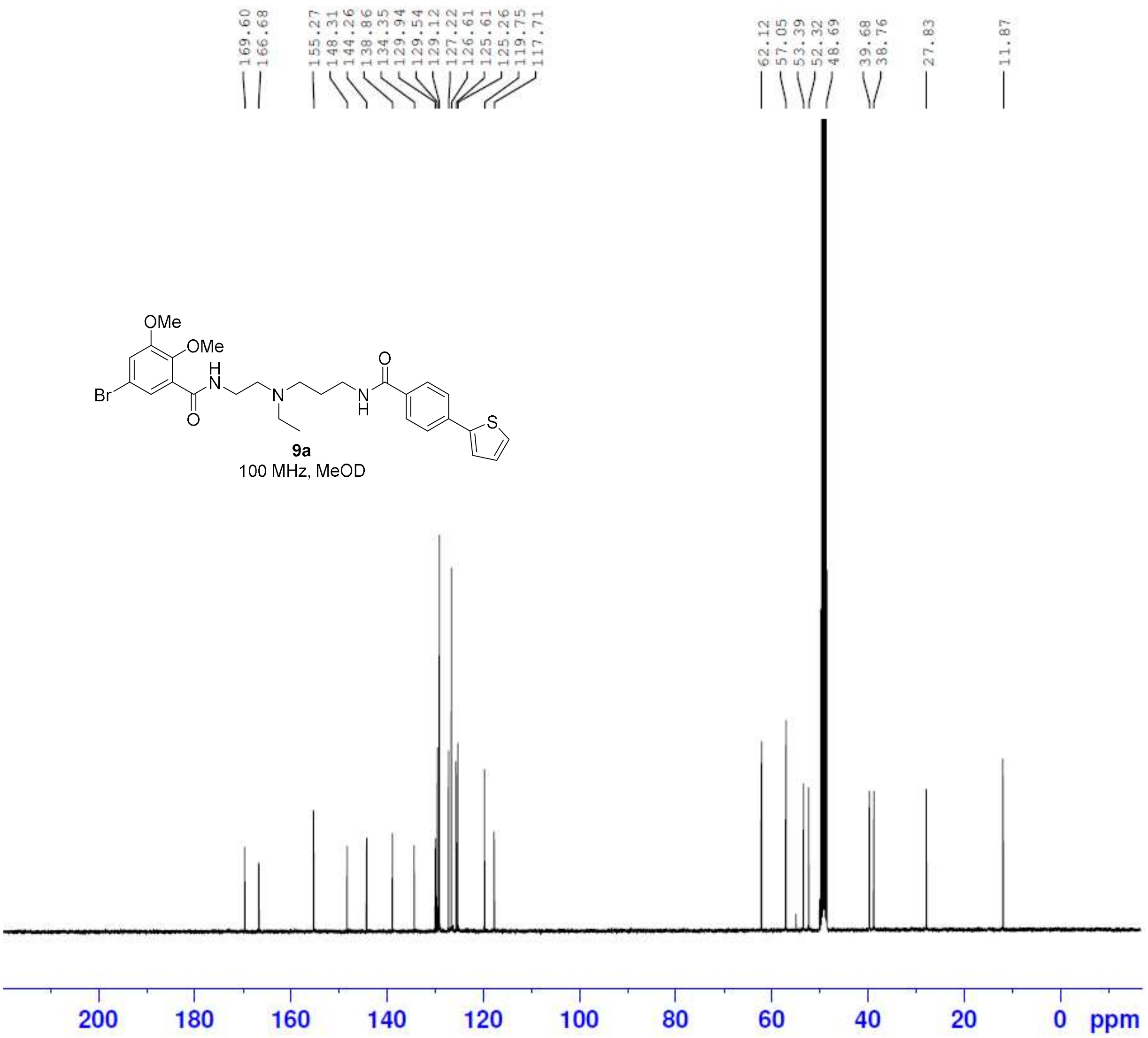
169.60
166.68
155.27
148.31
144.26
138.86
134.35
129.94
129.54
129.12
127.22
126.61
125.61
125.26
119.75
117.71
62.12
57.05
53.39
52.32
48.69
39.68
38.76
27.83
11.87
OMe
OMe
Br
H
N
O
N
N
H
O
S
9a
100 MHz, MeOD
200
180
160
140
120
100
80
60
40
20
0
ppm

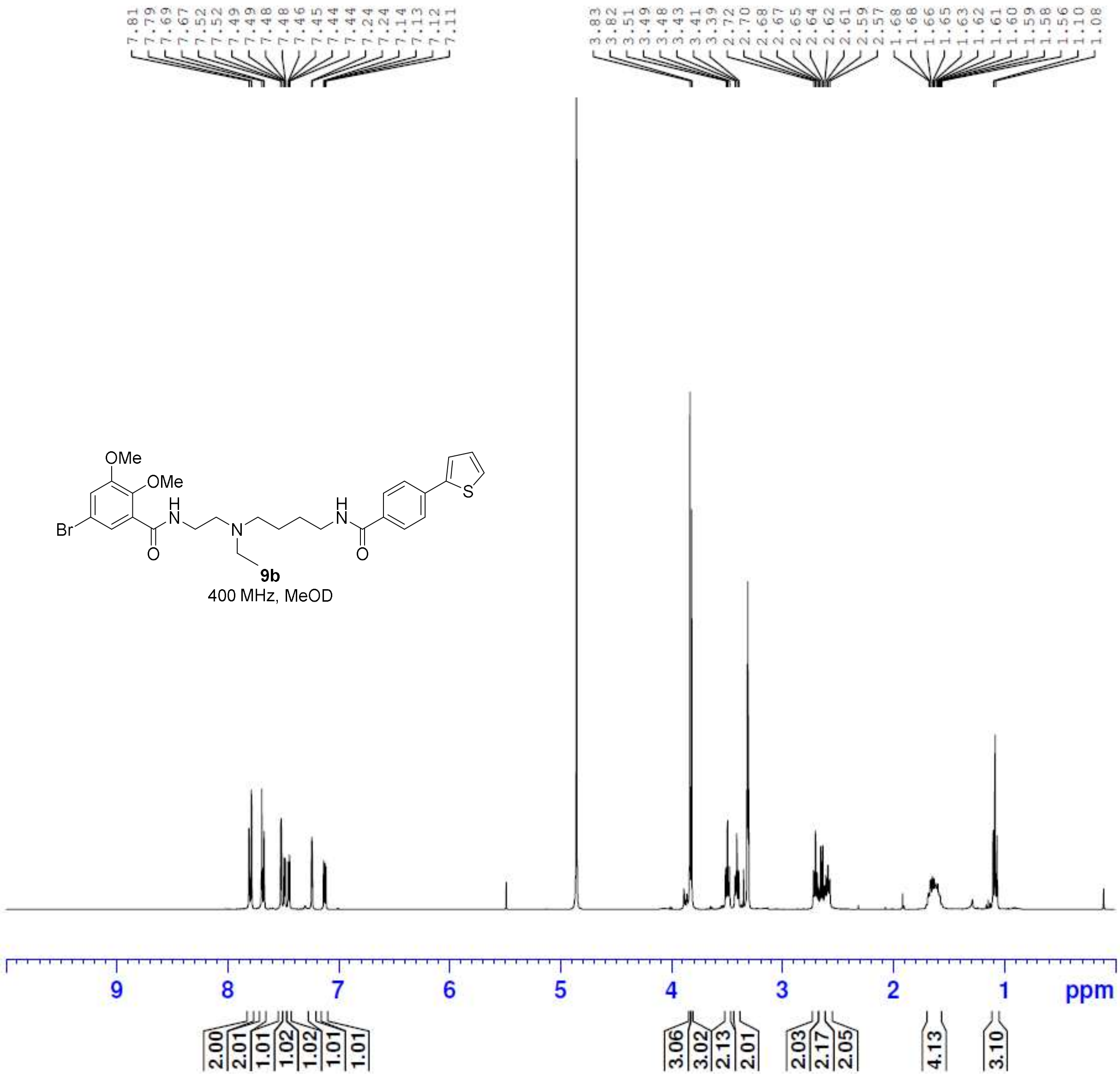
OMe
OMe
Br
H
N
N
H
N
O
O
S
9b
400 MHz, MeOD
ppm

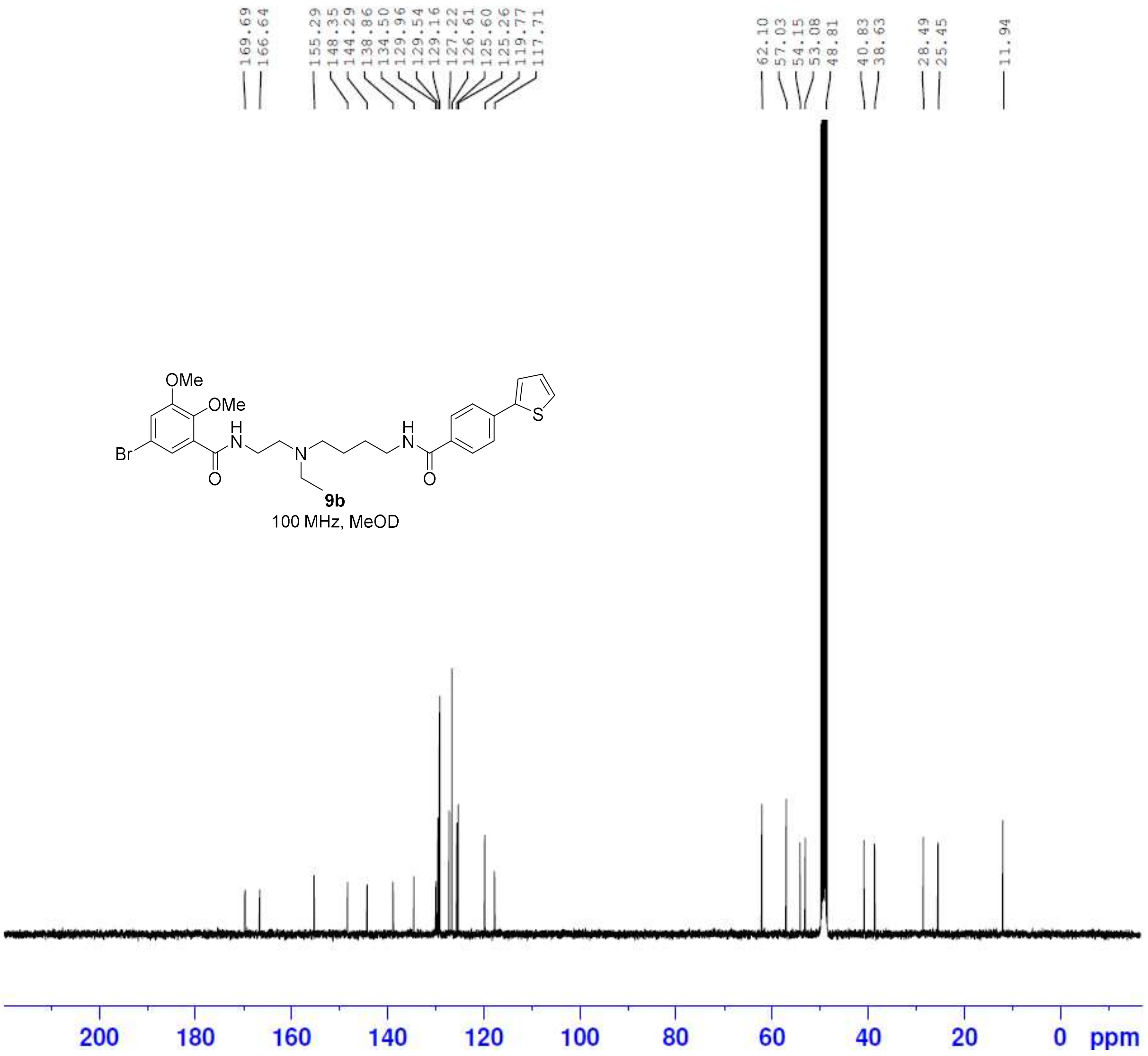
169.69
166.64
155.29
148.35
144.29
138.86
134.50
129.96
129.54
129.16
127.22
126.61
125.60
125.26
119.77
117.71
62.10
57.03
54.15
53.08
48.81
40.83
38.63
28.49
25.45
11.94
OMe
OMe
Br
H
N
N
H
N
O
O
S
9b
100 MHz, MeOD
200
180
160
140
120
100
80
60
40
20
0
ppm

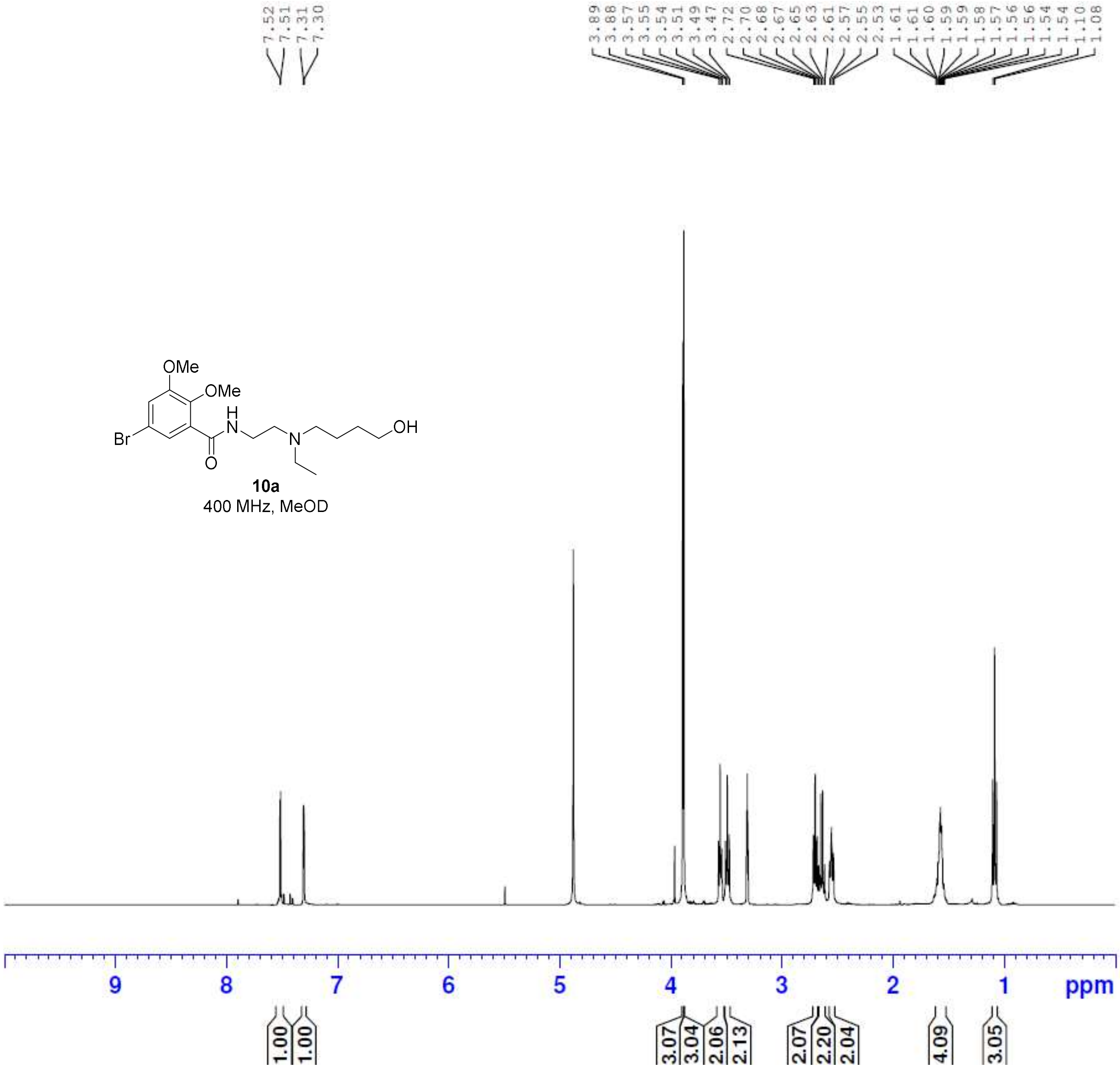

7.52
7.51
7.31
7.30
3.89
3.88
3.57
3.55
3.54
3.51
3.49
3.47
2.72
2.70
2.68
2.67
2.65
2.63
2.61
2.57
2.55
2.53
1.61
1.61
1.60
1.59
1.59
1.58
1.57
1.56
1.54
1.54
1.10
1.08
OMe
OMe
H
N
Br
O
N
OH
10a
400 MHz, MeOD
9
8
7
6
5
4
3
2
1
ppm
1.00
1.00
3.07
3.04
2.06
2.13
2.07
2.20
2.04
4.09
3.05

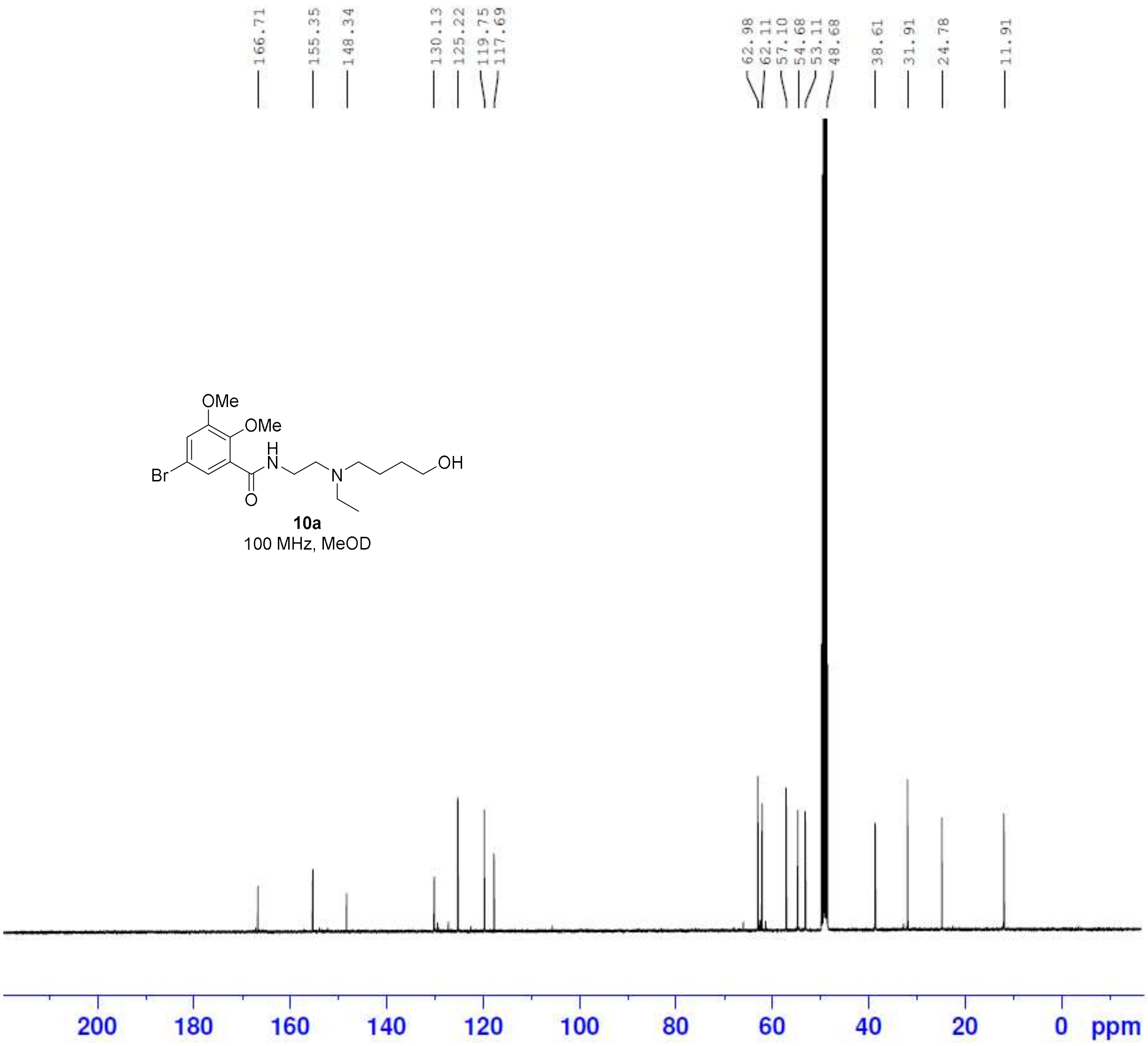
166.71
155.35
148.34
130.13
125.22
119.75
117.69
62.98
62.11
57.10
54.68
53.11
48.68
38.61
31.91
24.78
11.91
OMe
OMe
H
N
N
OH
Br
O
10a
100 MHz, MeOD
200
180
160
140
120
100
80
60
40
20
0
ppm

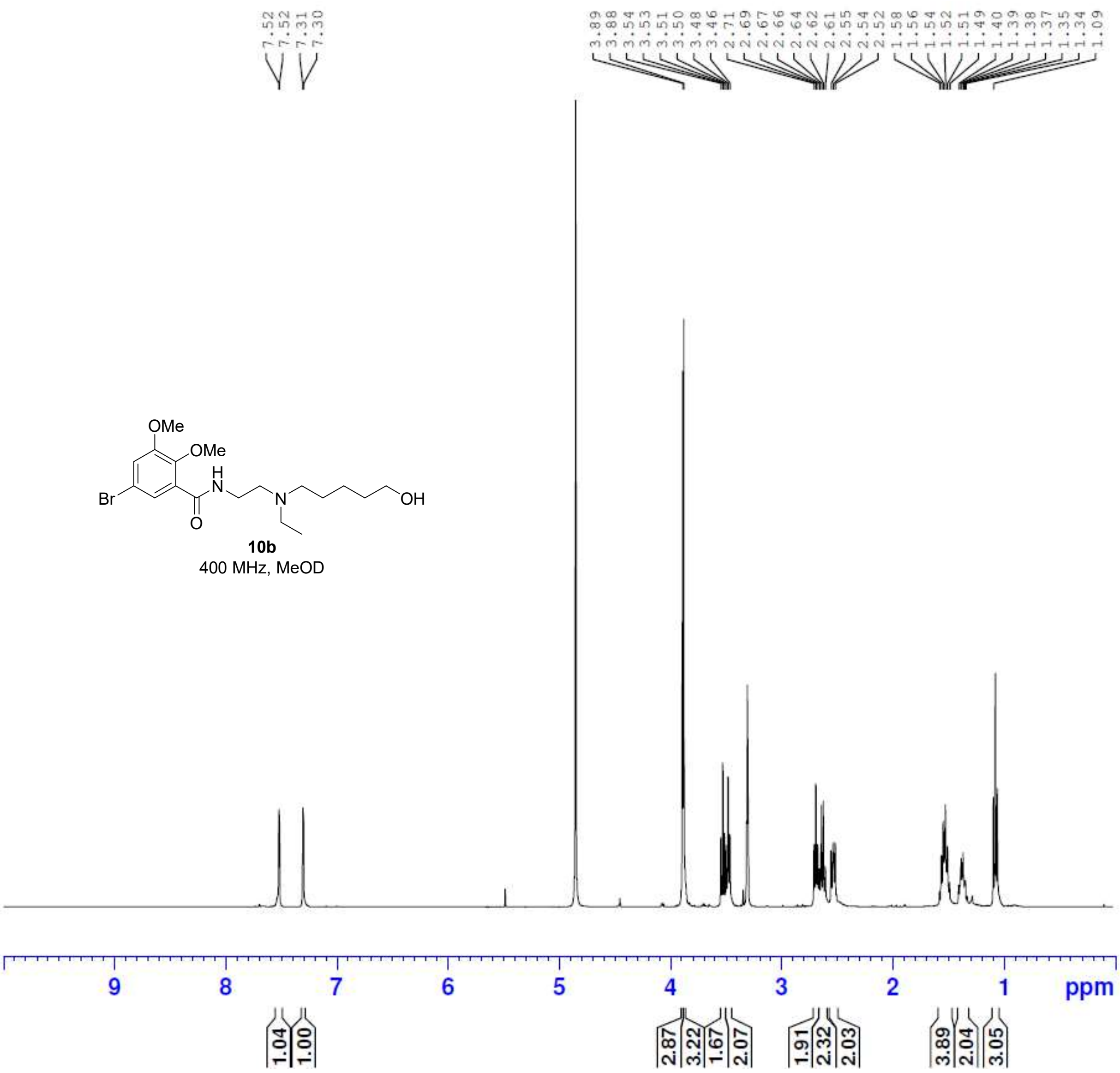
OMe
OMe
H
N
Br
O
N
OH
10b
400 MHz, MeOD
7.52
7.52
7.31
7.30
3.89
3.88
3.54
3.53
3.51
3.50
3.48
3.46
2.71
2.69
2.67
2.66
2.64
2.62
2.61
2.55
2.54
2.52
1.58
1.56
1.54
1.52
1.51
1.49
1.40
1.39
1.38
1.37
1.35
1.34
1.09
9
8
7
6
5
4
3
2
1
ppm
1.04
1.00
2.87
3.22
1.67
2.07
1.91
2.32
2.03
3.89
2.04
3.05

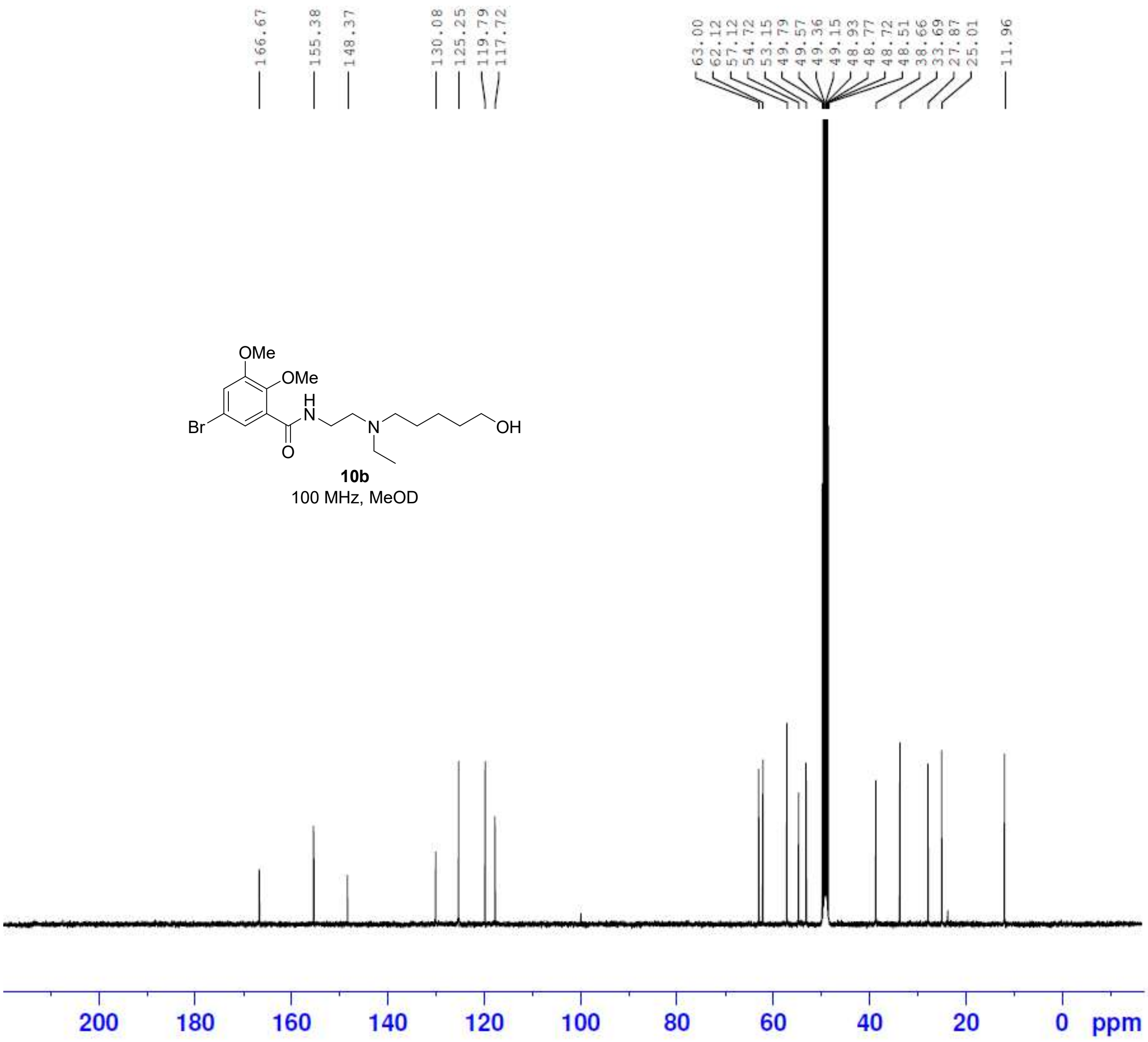

10b
100 MHz, MeOD

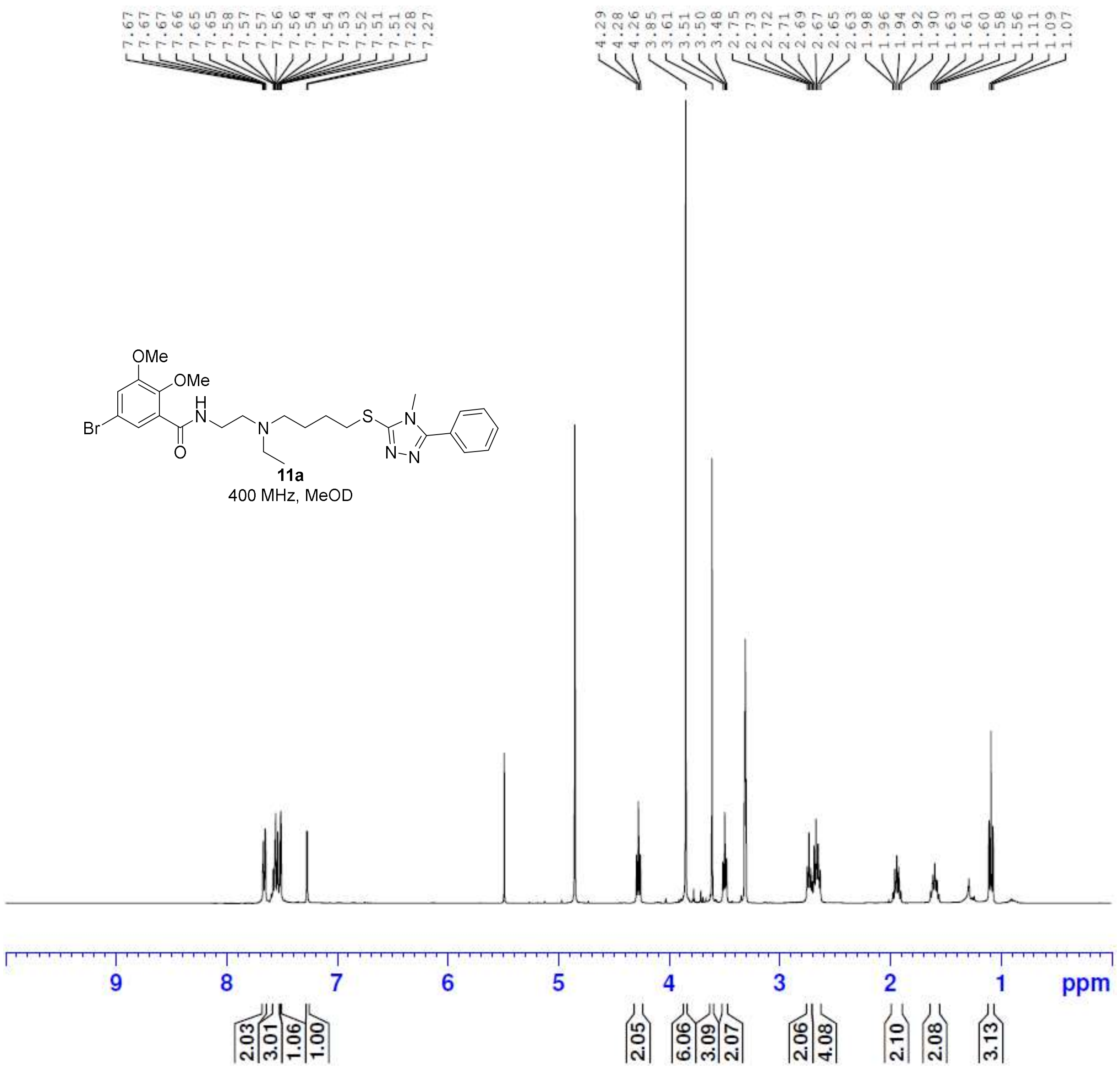

7.67 7.67 7.66 7.65 7.65 7.58 7.57 7.57 7.56 7.56 7.54 7.54 7.53 7.52 7.51 7.51 7.28 7.27
4.29 4.28 4.26 3.85 3.61 3.51 3.50 3.48 2.75 2.73 2.72 2.71 2.69 2.67 2.65 2.63 1.98 1.96 1.94 1.92 1.90 1.63 1.61 1.60 1.58 1.56 1.11 1.09 1.07
OMe
OMe
H
N
Br
O
N
S
N
N
N
11a
400 MHz, MeOD
9
8
7
6
5
4
3
2
1
ppm
2.03 3.01 1.06 1.00
2.05 6.06 3.09 2.07 2.06 4.08 2.10 2.08 3.13

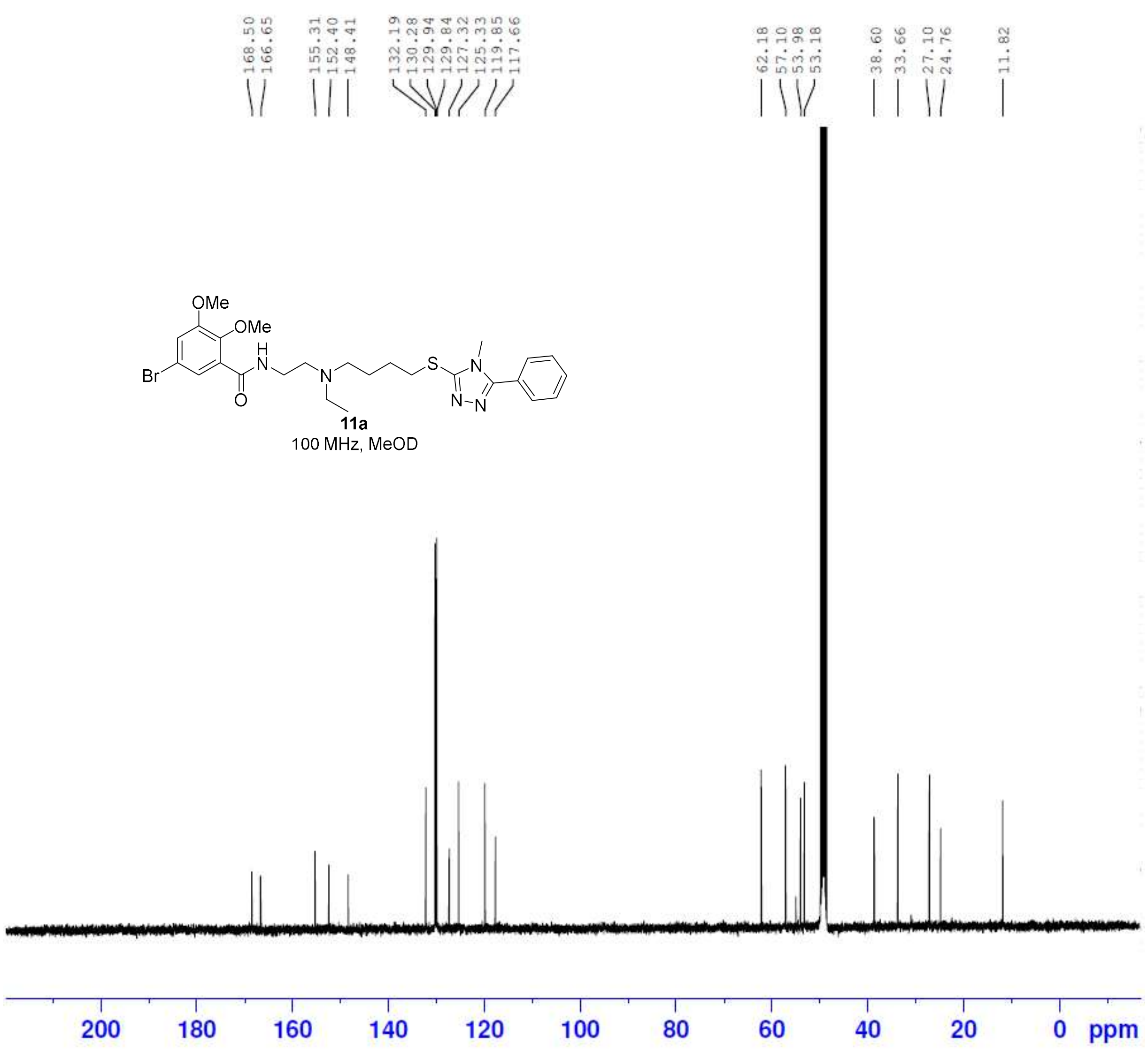
168.50
166.65
155.31
152.40
148.41
132.19
130.28
129.94
129.84
127.32
125.33
119.85
117.66
62.18
57.10
53.98
53.18
38.60
33.66
27.10
24.76
11.82
OMe
OMe
H
N
Br
O
N
S
N
N
N
11a
100 MHz, MeOD
200
180
160
140
120
100
80
60
40
20
0
ppm

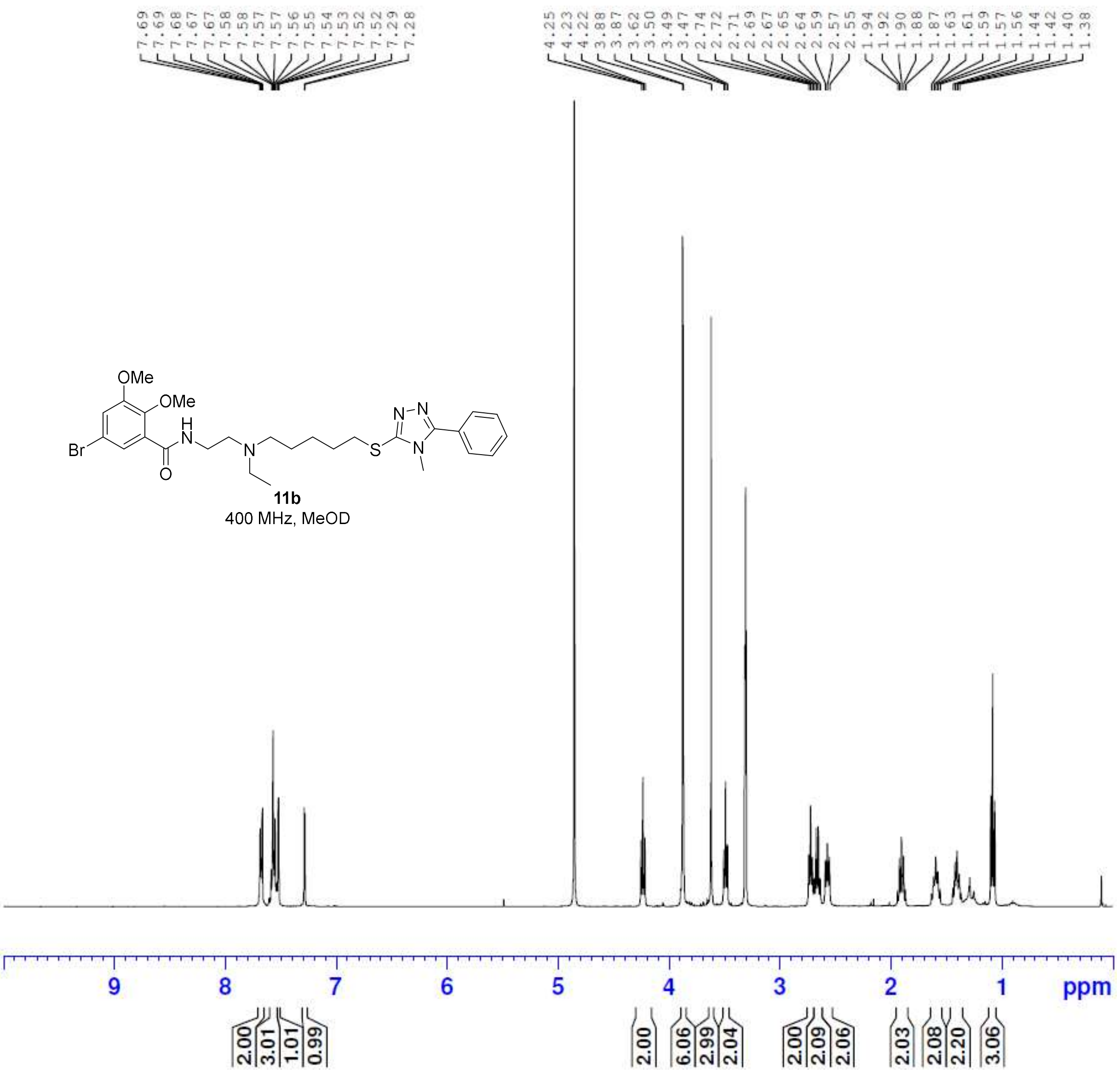
7.69 7.69 7.68 7.67 7.67 7.58 7.57 7.57 7.56 7.55 7.54 7.53 7.52 7.52 7.29 7.28
4.25 4.23 4.22 3.88 3.87 3.62 3.50 3.49 3.47 2.74 2.72 2.71 2.69 2.67 2.65 2.64 2.59 2.57 2.55 1.94 1.92 1.90 1.88 1.87 1.63 1.61 1.59 1.57 1.56 1.44 1.42 1.40 1.38
OMe
OMe
H
N
Br
O
N
S
N–N
N
11b
400 MHz, MeOD
9 8 7 6 5 4 3 2 1 ppm
2.00 3.01 1.01 0.99
2.00 6.06 2.99 2.04
2.00 2.09 2.06
2.03 2.08 2.20 3.06

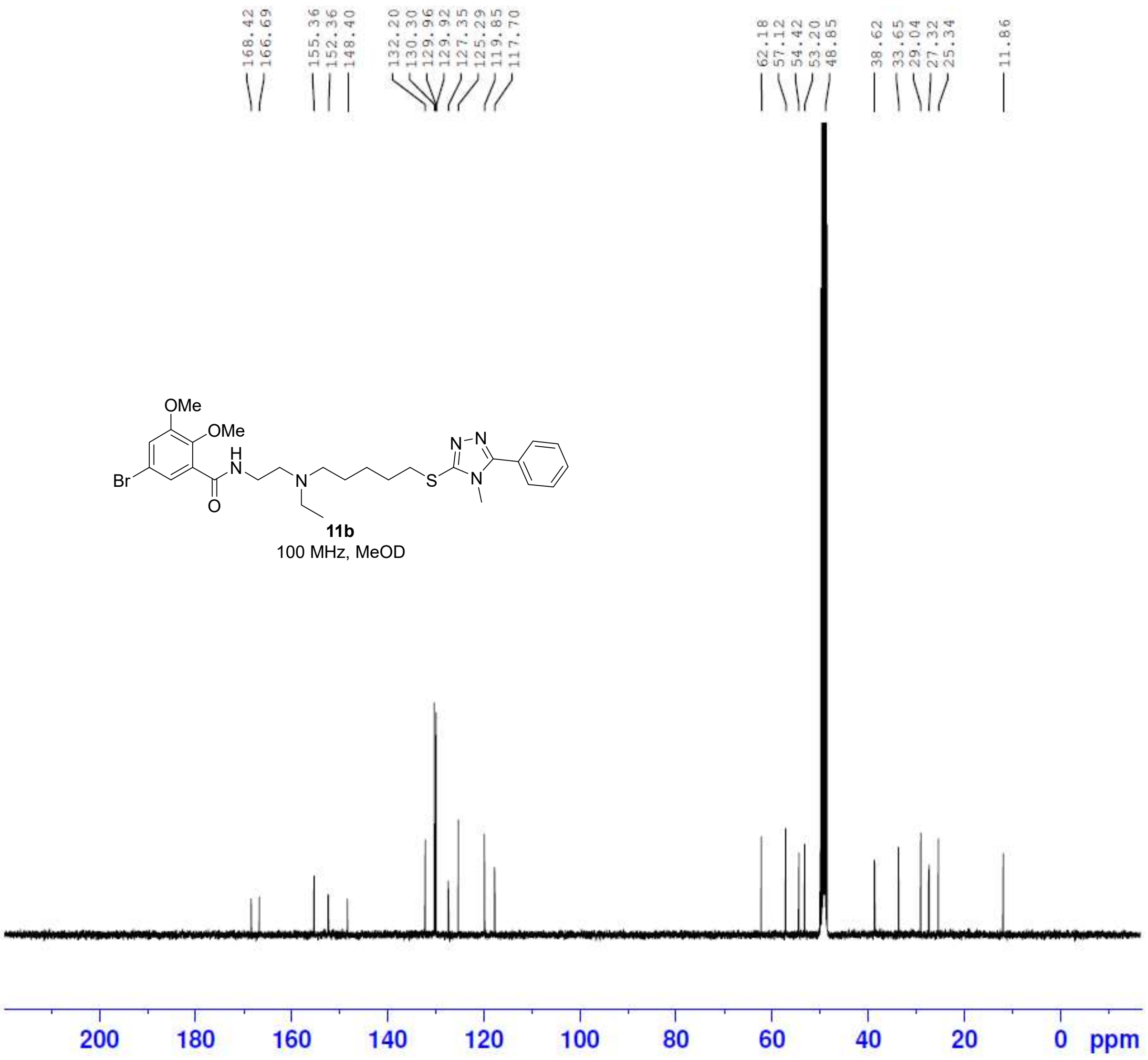
168.42
166.69
155.36
152.36
148.40
132.20
130.30
129.96
129.92
127.35
125.29
119.85
117.70
62.18
57.12
54.42
53.20
48.85
38.62
33.65
29.04
27.32
25.34
11.86
OMe
OMe
H
N
Br
O
N
S
N–N
N
11b
100 MHz, MeOD
200
180
160
140
120
100
80
60
40
20
0
ppm

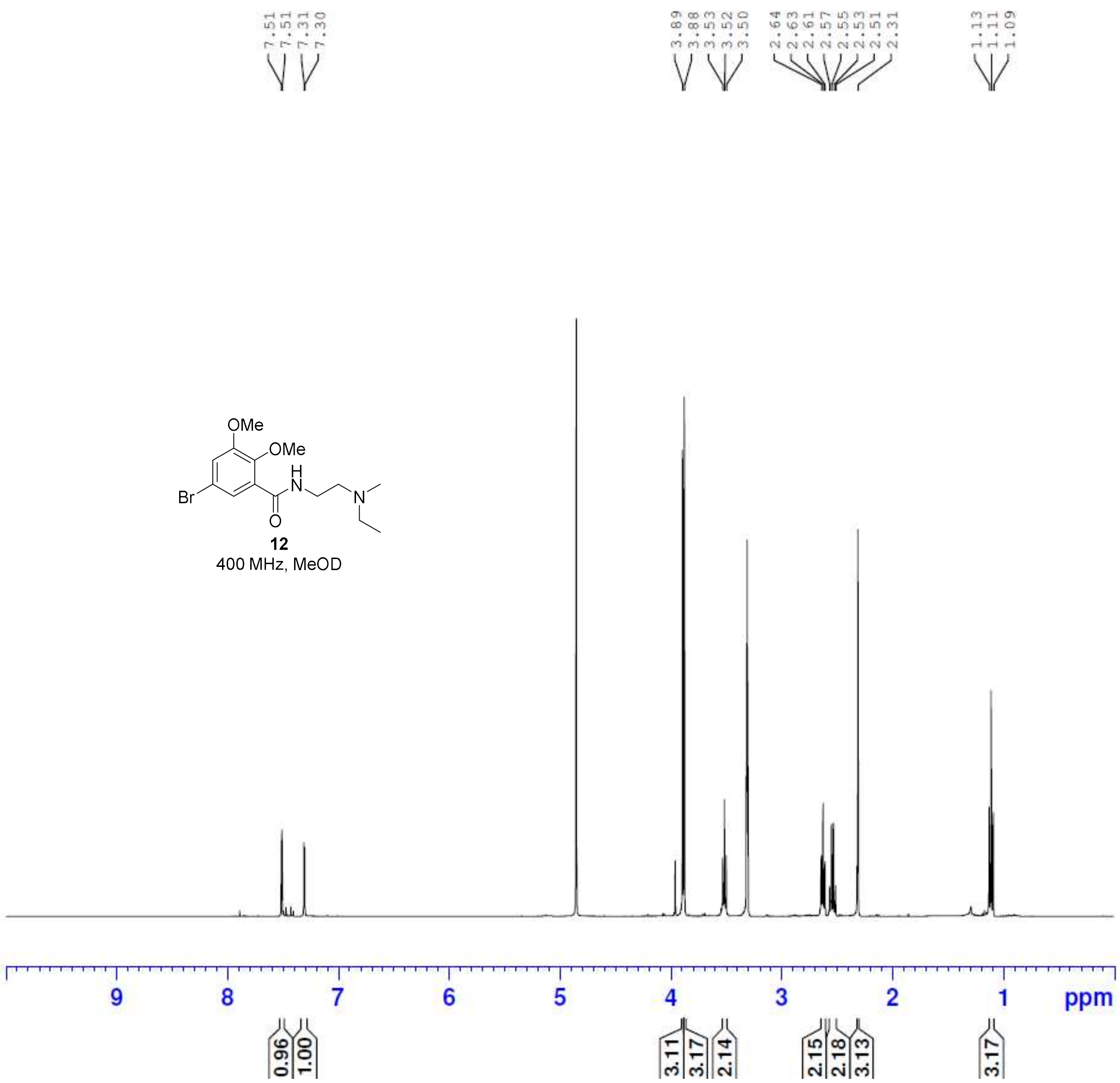

7.51
7.51
7.31
7.30
3.89
3.88
3.53
3.52
3.50
2.64
2.63
2.61
2.57
2.55
2.53
2.51
2.31
1.13
1.11
1.09
OMe
OMe
H
N
Br
O
N
12
400 MHz, MeOD
9
8
7
6
5
4
3
2
1
ppm
0.96
1.00
3.11
3.17
2.14
2.15
2.18
3.13
3.17

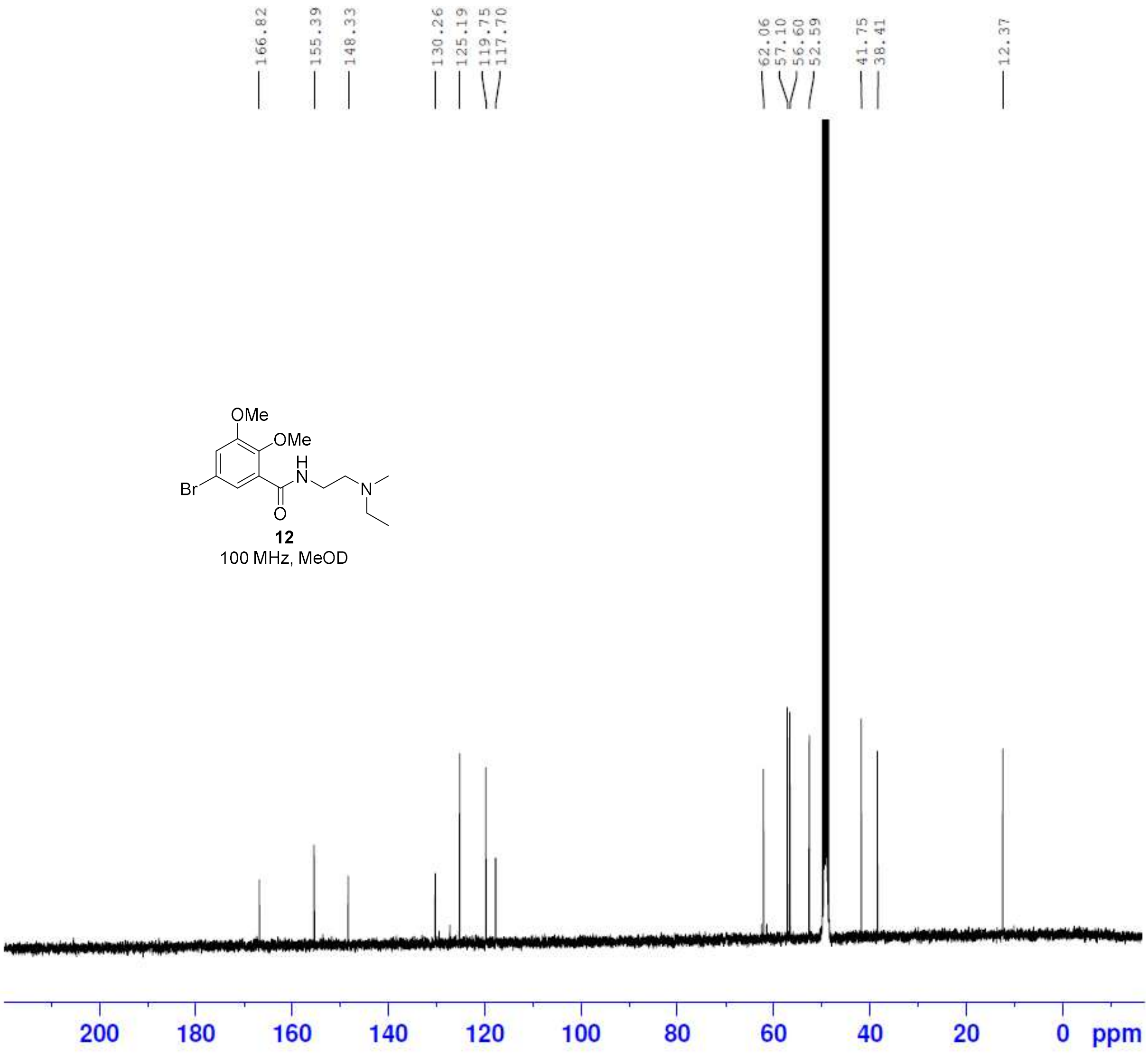

12
100 MHz, MeOD

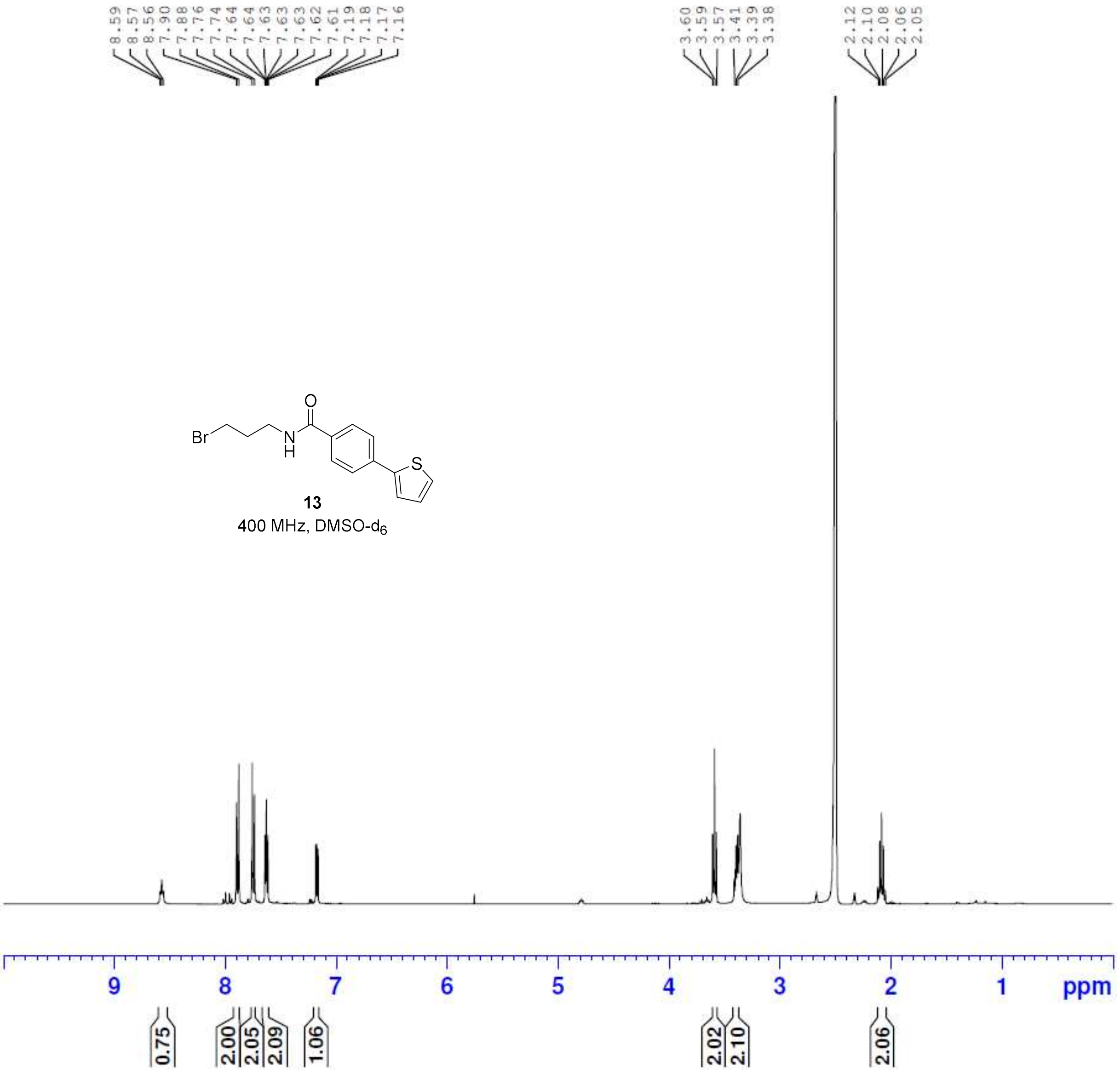
8.59
8.57
8.56
7.90
7.88
7.76
7.74
7.64
7.64
7.63
7.63
7.63
7.62
7.61
7.19
7.18
7.17
7.16
3.60
3.59
3.57
3.41
3.39
3.38
2.12
2.10
2.08
2.06
2.05
Br
N
H
O
S
13
400 MHz, DMSO-d6
9
8
7
6
5
4
3
2
1
ppm
0.75
2.00
2.05
2.09
1.06
2.02
2.10
2.06

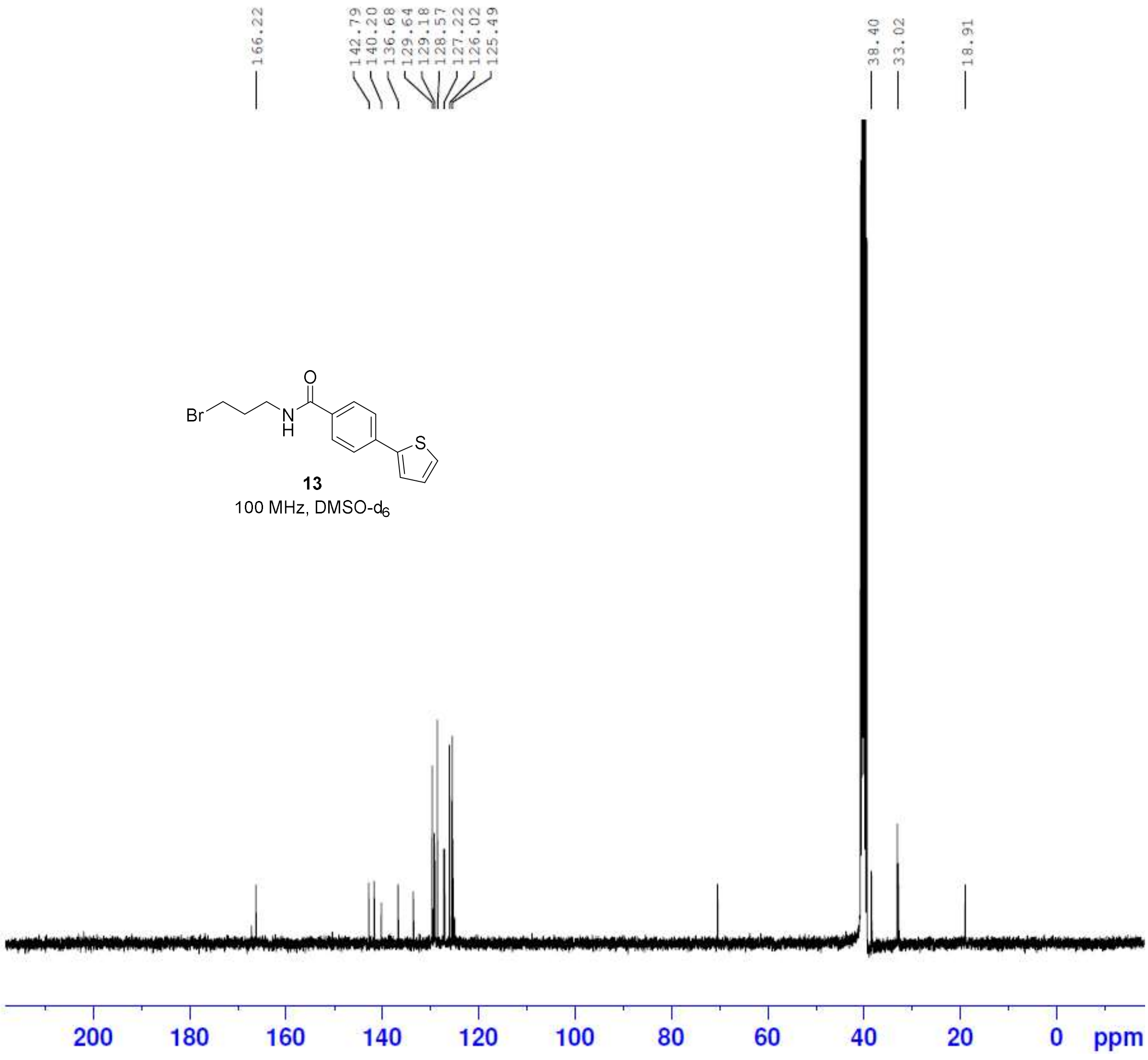

166.22
142.79
140.20
136.68
129.64
129.18
128.57
127.22
126.02
125.49
38.40
33.02
18.91
Br
N
H
O
S
13
100 MHz, DMSO-d_6
200
180
160
140
120
100
80
60
40
20
0
ppm

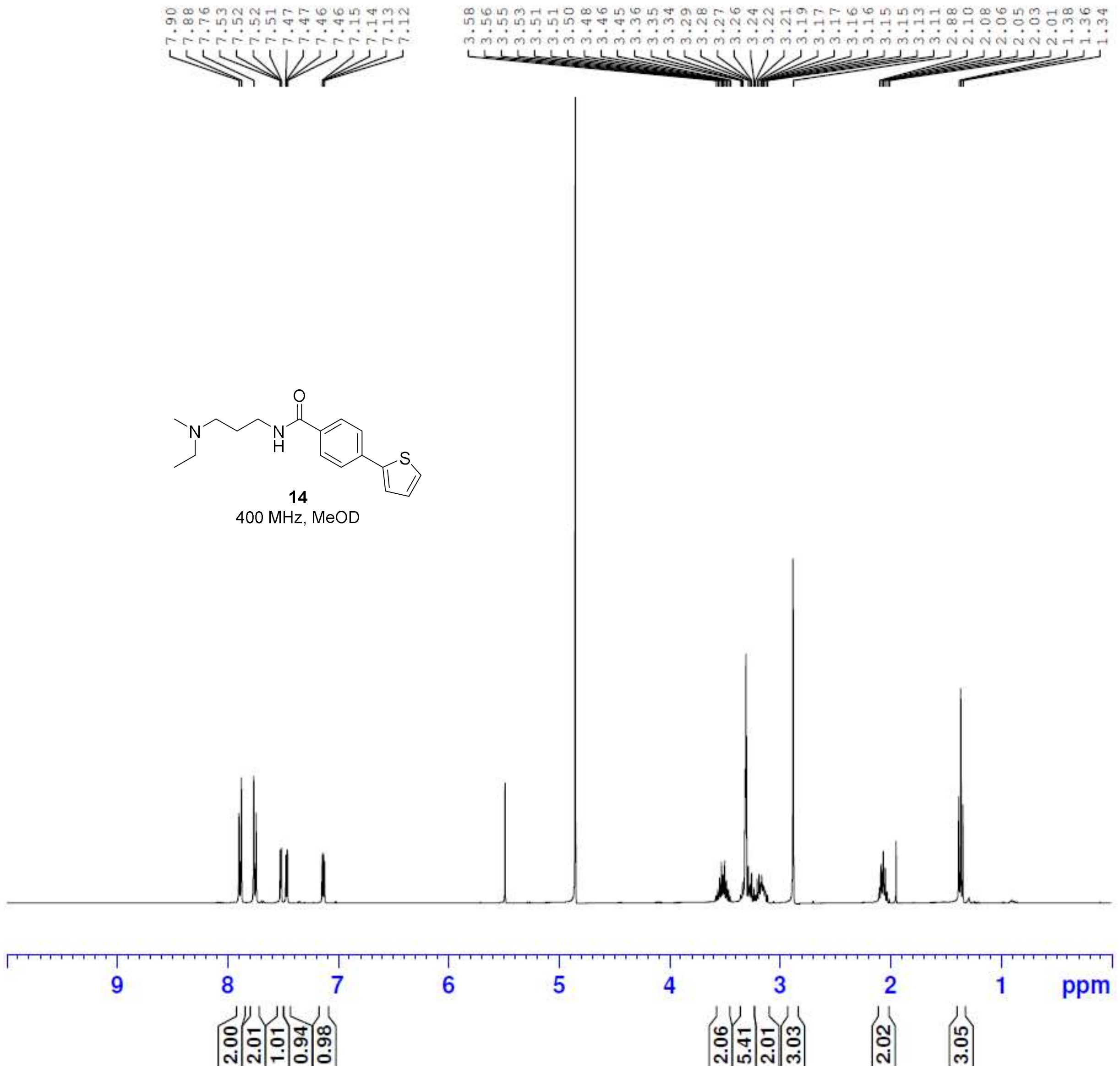

7.90 7.88 7.76 7.53 7.52 7.52 7.51 7.47 7.47 7.46 7.46 7.15 7.14 7.13 7.12
3.58 3.56 3.55 3.53 3.51 3.51 3.50 3.48 3.46 3.45 3.36 3.35 3.34 3.29 3.28 3.27 3.26 3.24 3.22 3.21 3.19 3.17 3.17 3.16 3.16 3.15 3.15 3.13 3.11 2.88 2.10 2.08 2.06 2.05 2.03 2.01 1.38 1.36 1.34
14
400 MHz, MeOD
9 8 7 6 5 4 3 2 1 ppm
2.00 2.01 1.01 0.94 0.98
2.06 5.41 2.01 3.03
2.02
3.05

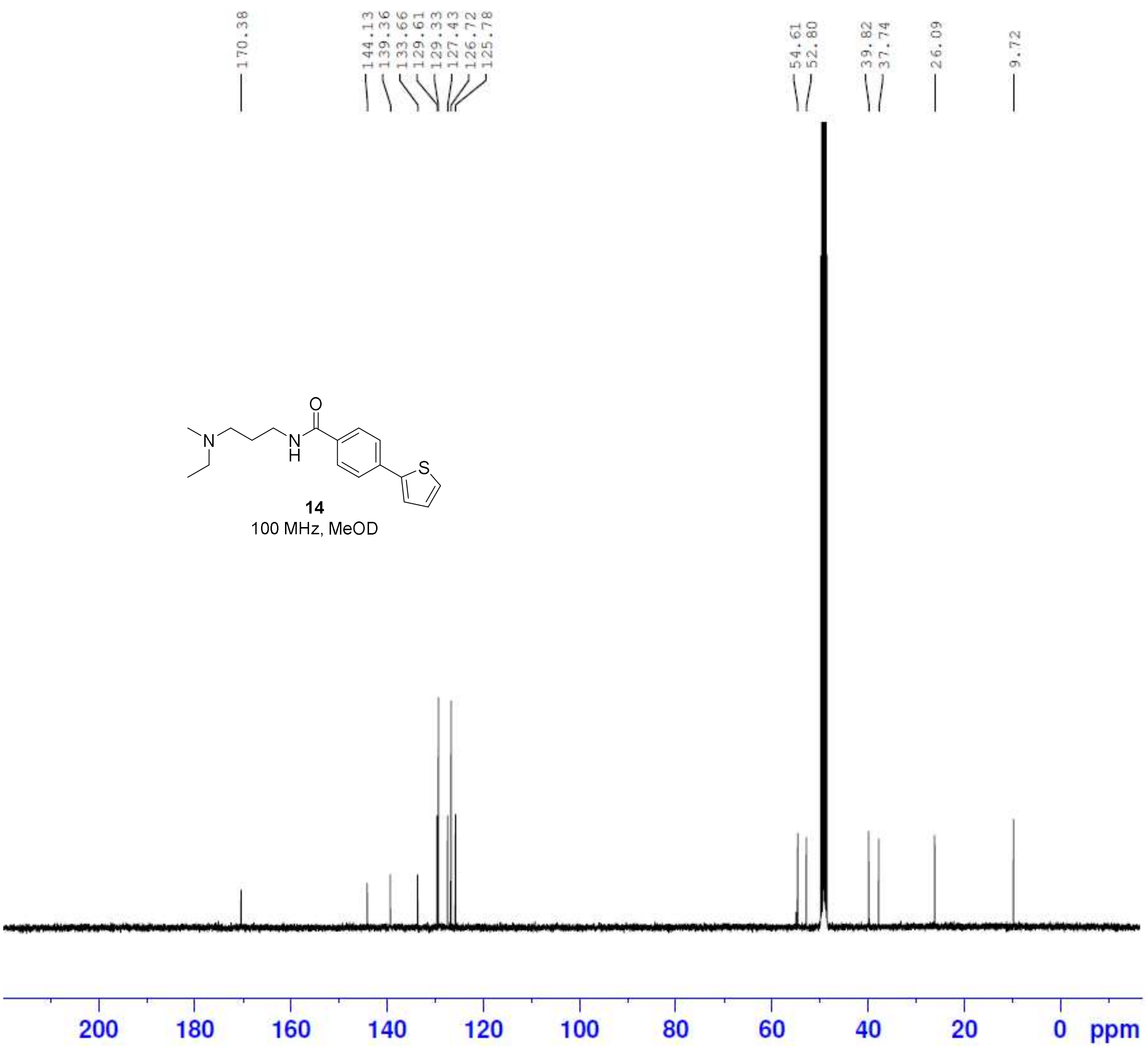

14
100 MHz, MeOD

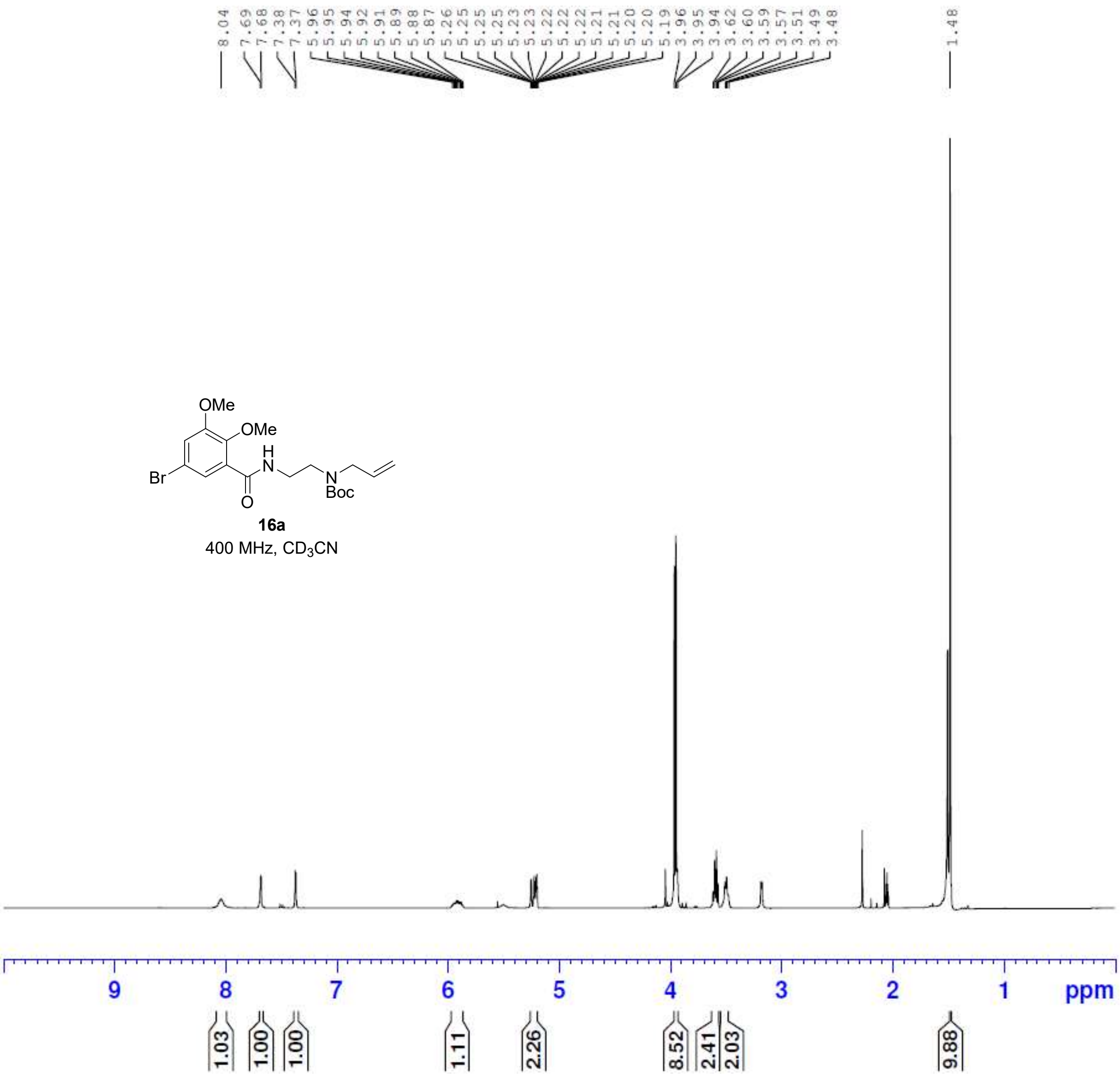

16a
400 MHz, CD_3CN

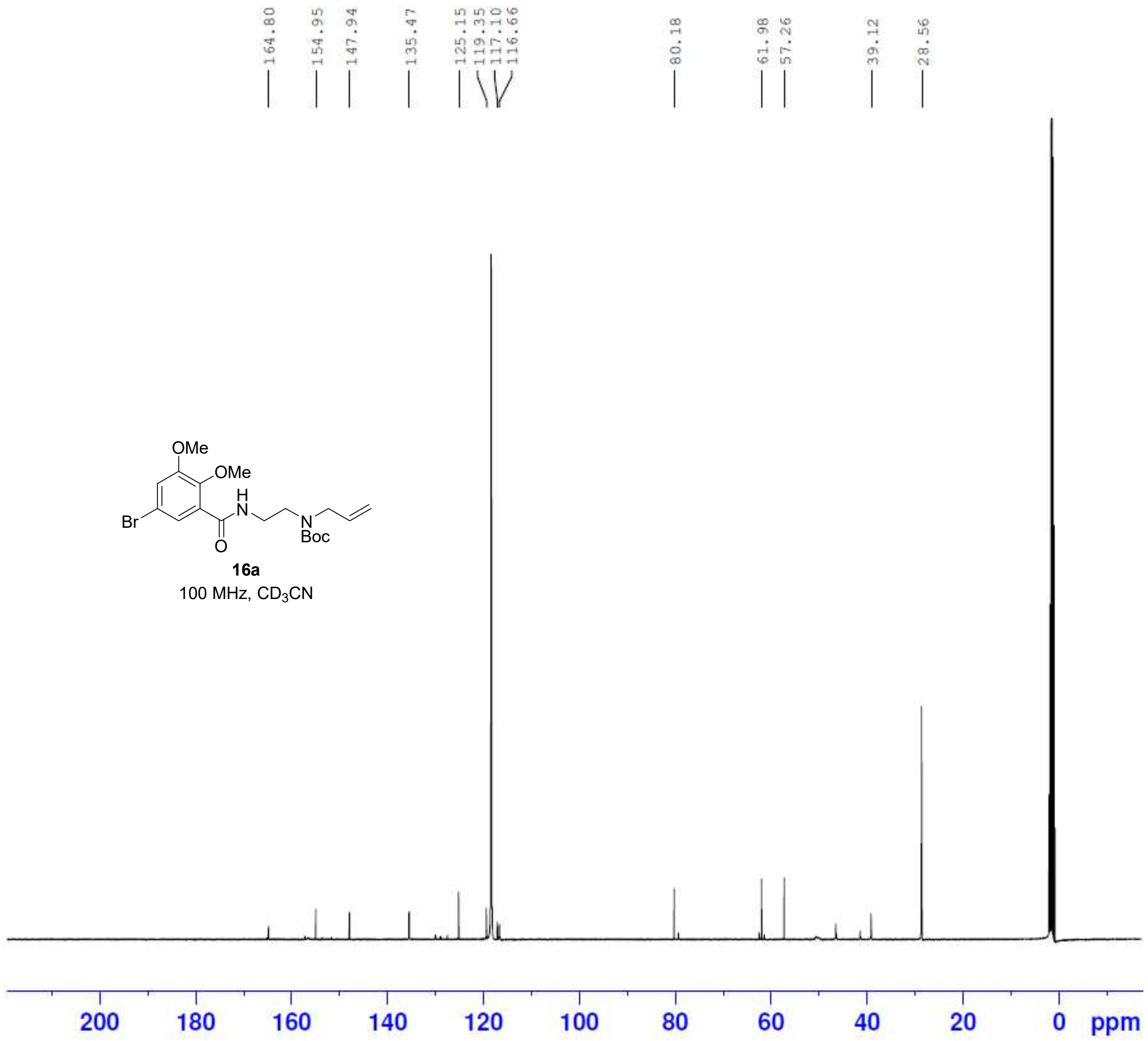
164.80
154.95
147.94
135.47
125.15
119.35
117.10
116.66
80.18
61.98
57.26
39.12
28.56
OMe
OMe
H
N
Br
O
N
Boc
16a
100 MHz, CD_3CN
200
180
160
140
120
100
80
60
40
20
0
ppm

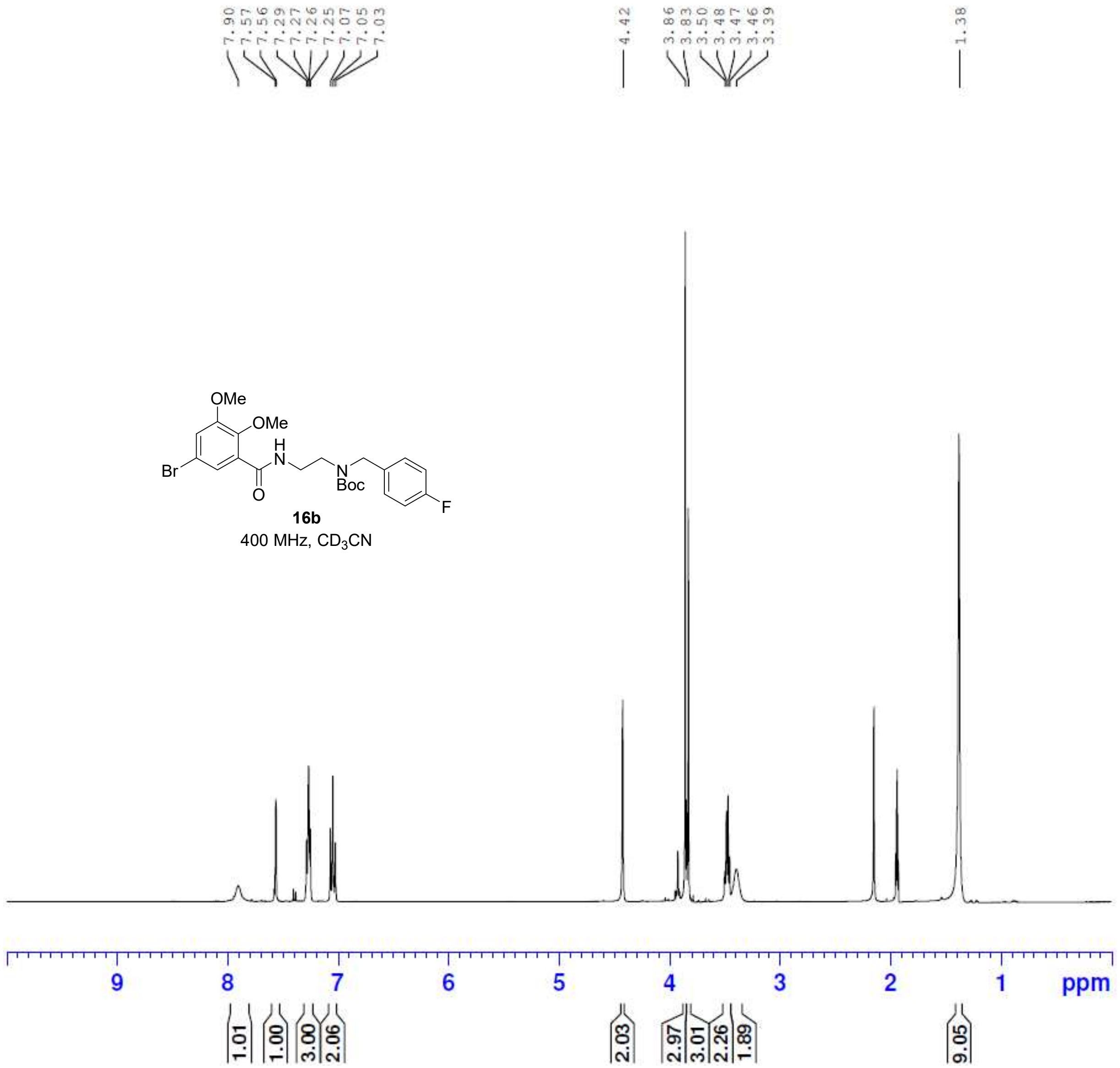
7.90
7.57
7.56
7.29
7.27
7.26
7.25
7.07
7.05
7.03
4.42
3.86
3.83
3.50
3.48
3.47
3.46
3.39
1.38
OMe
OMe
H
N
Br
O
N
Boc
F
16b
400 MHz, CD_3CN
9
8
7
6
5
4
3
2
1
ppm
1.01
1.00
3.00
2.06
2.03
2.97
3.01
2.26
1.89
9.05

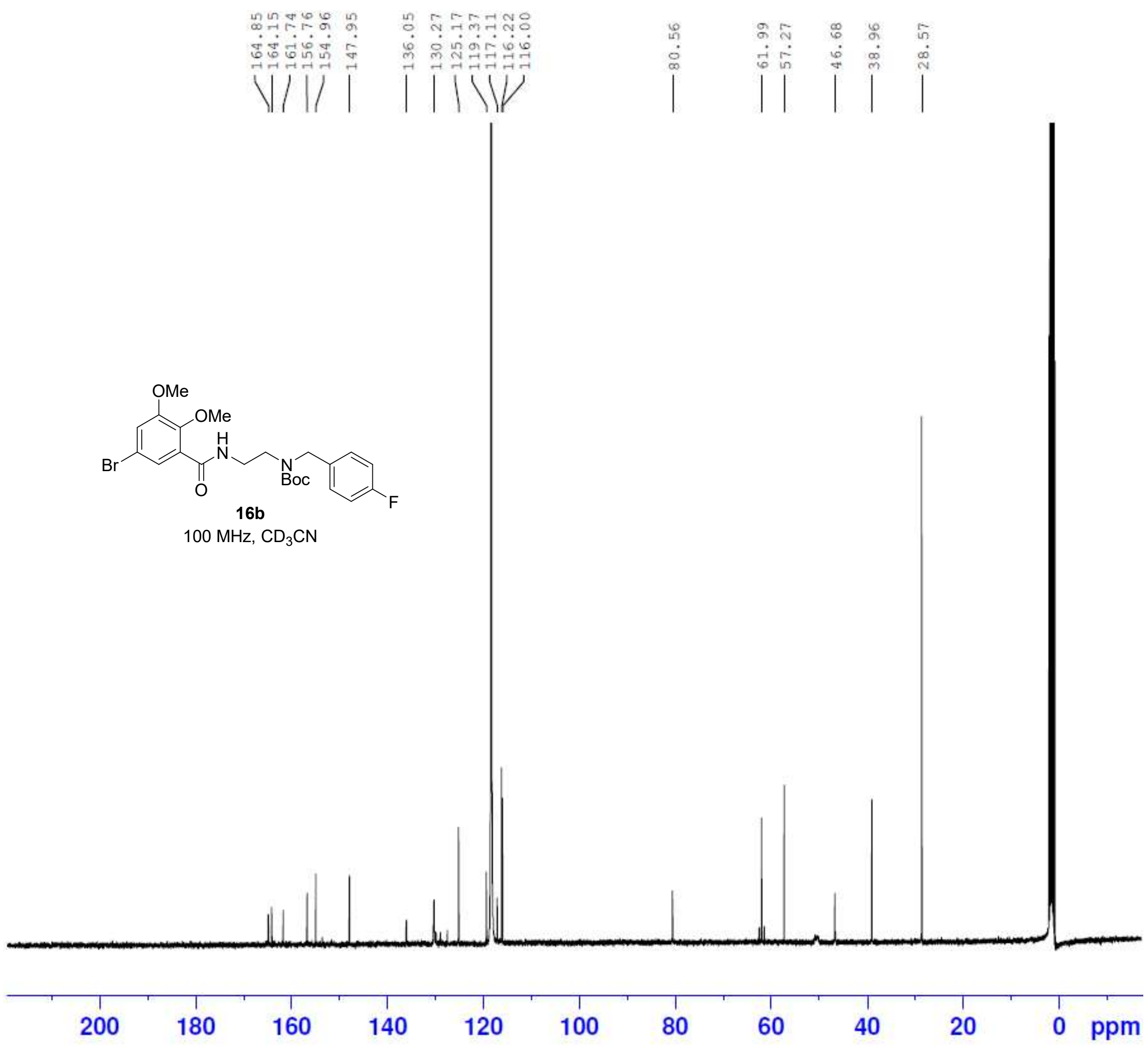

16b
100 MHz, CD_3CN

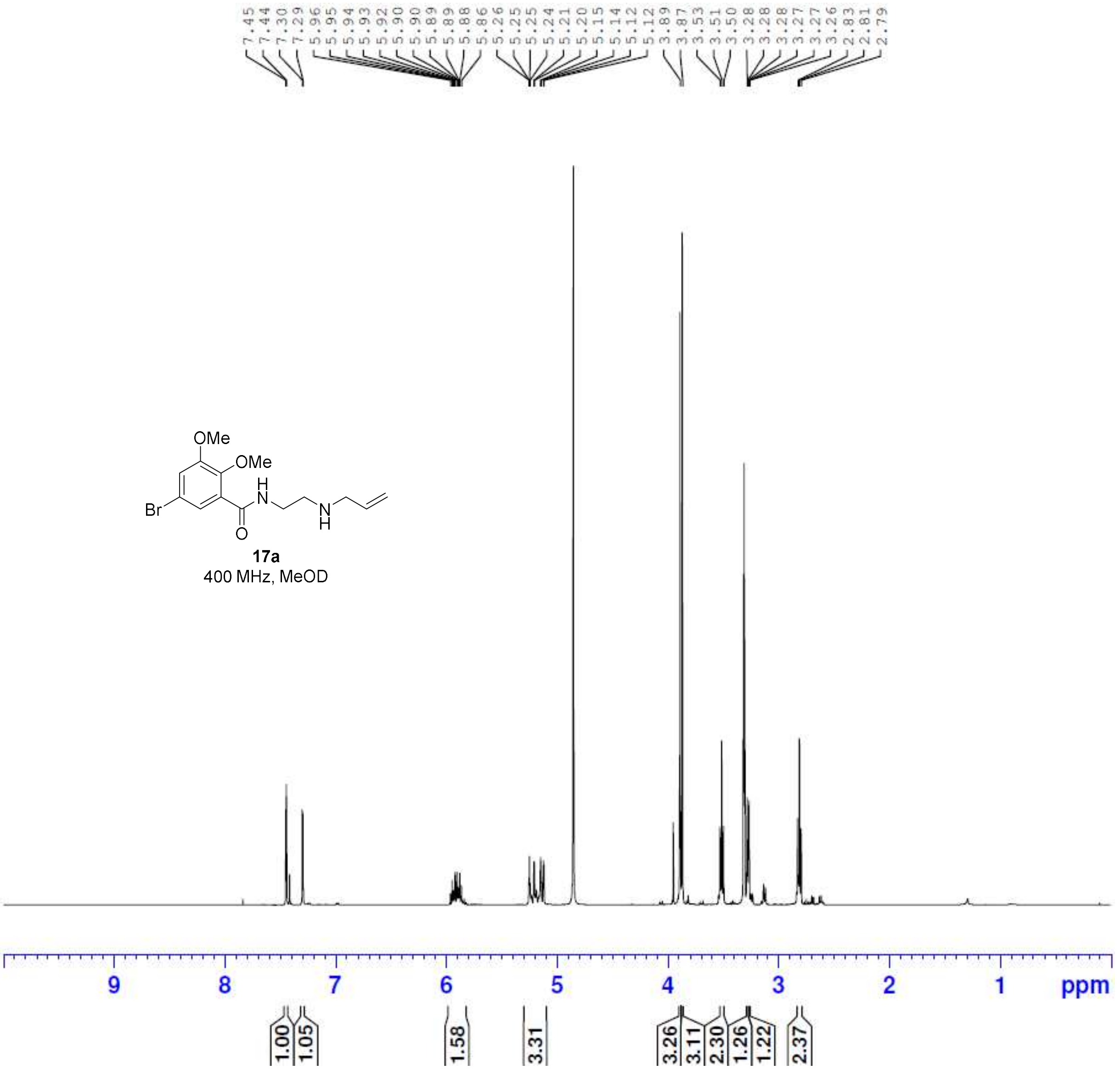
7.45
7.44
7.30
7.29
5.96
5.95
5.94
5.93
5.92
5.90
5.90
5.89
5.89
5.88
5.88
5.86
5.26
5.25
5.25
5.24
5.21
5.20
5.15
5.14
5.12
5.12
3.89
3.87
3.53
3.51
3.50
3.28
3.28
3.28
3.27
3.27
3.26
2.83
2.81
2.79
OMe
OMe
H
N
Br
N
H
O
17a
400 MHz, MeOD
9
8
7
6
5
4
3
2
1
ppm
1.00
1.05
1.58
3.31
3.26
3.11
2.30
1.26
1.22
2.37

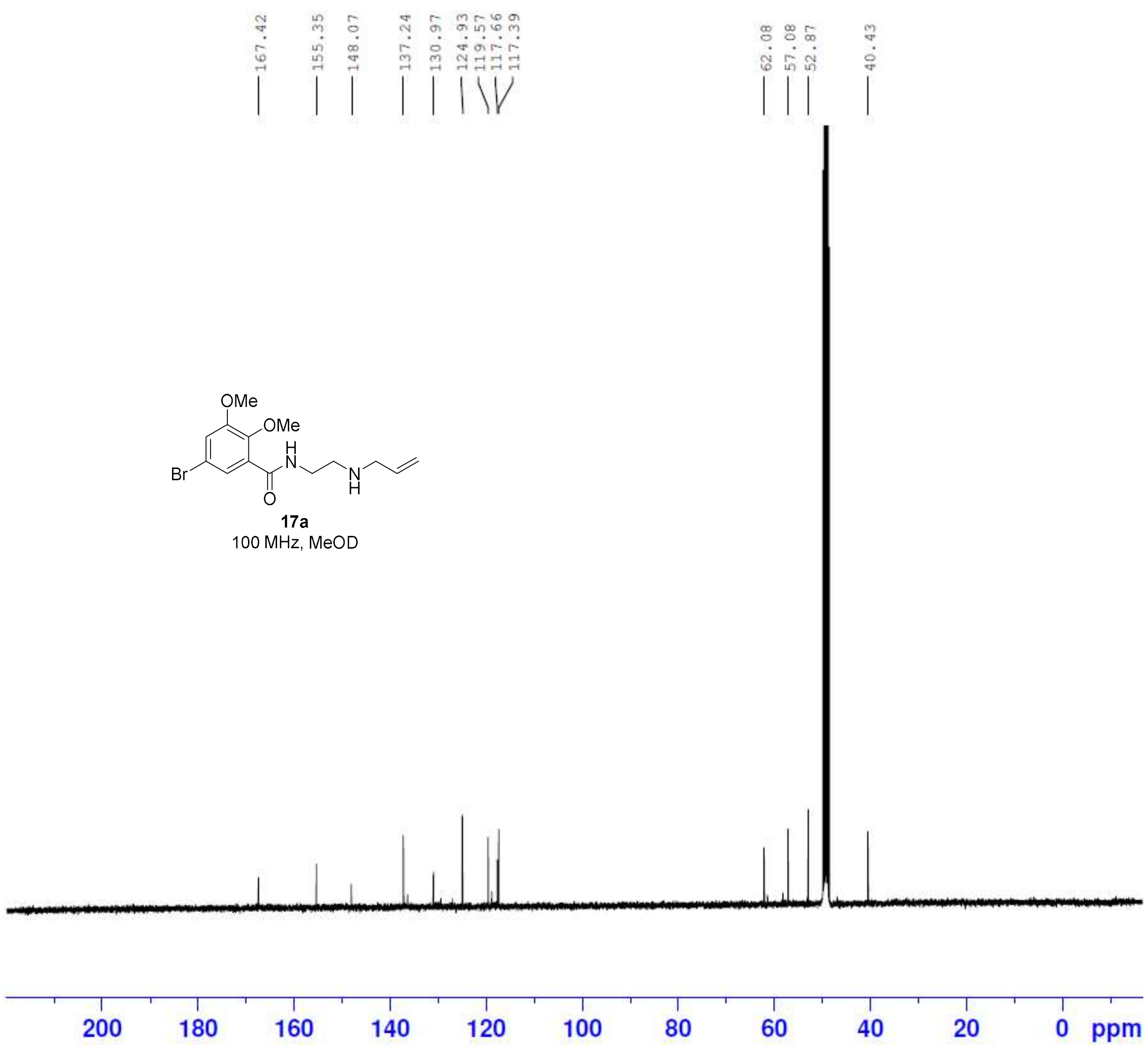
167.42
155.35
148.07
137.24
130.97
124.93
119.57
117.66
117.39
62.08
57.08
52.87
40.43
OMe
OMe
Br
H
N
N
H
O
17a
100 MHz, MeOD
200
180
160
140
120
100
80
60
40
20
0
ppm

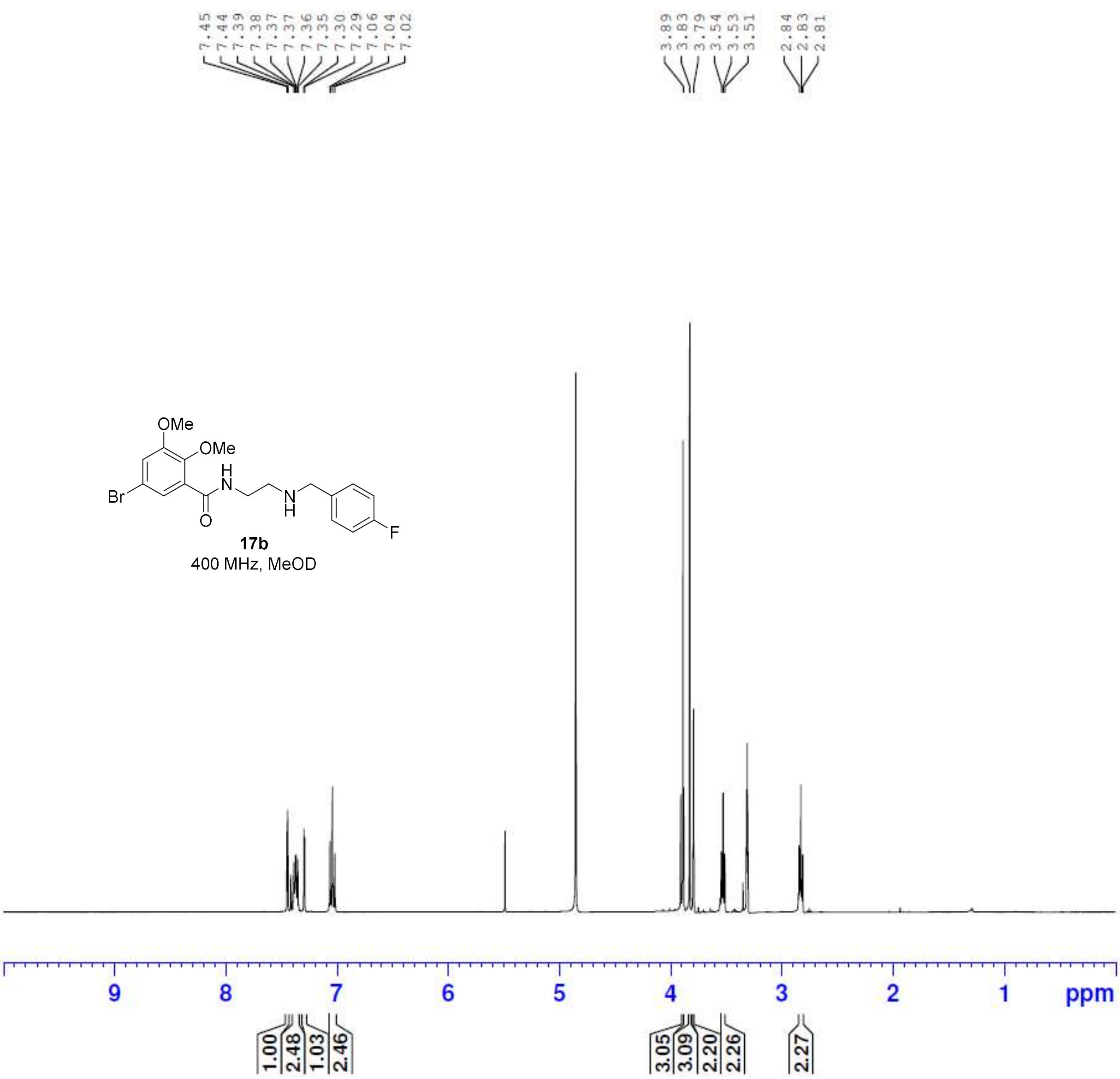

17b
400 MHz, MeOD

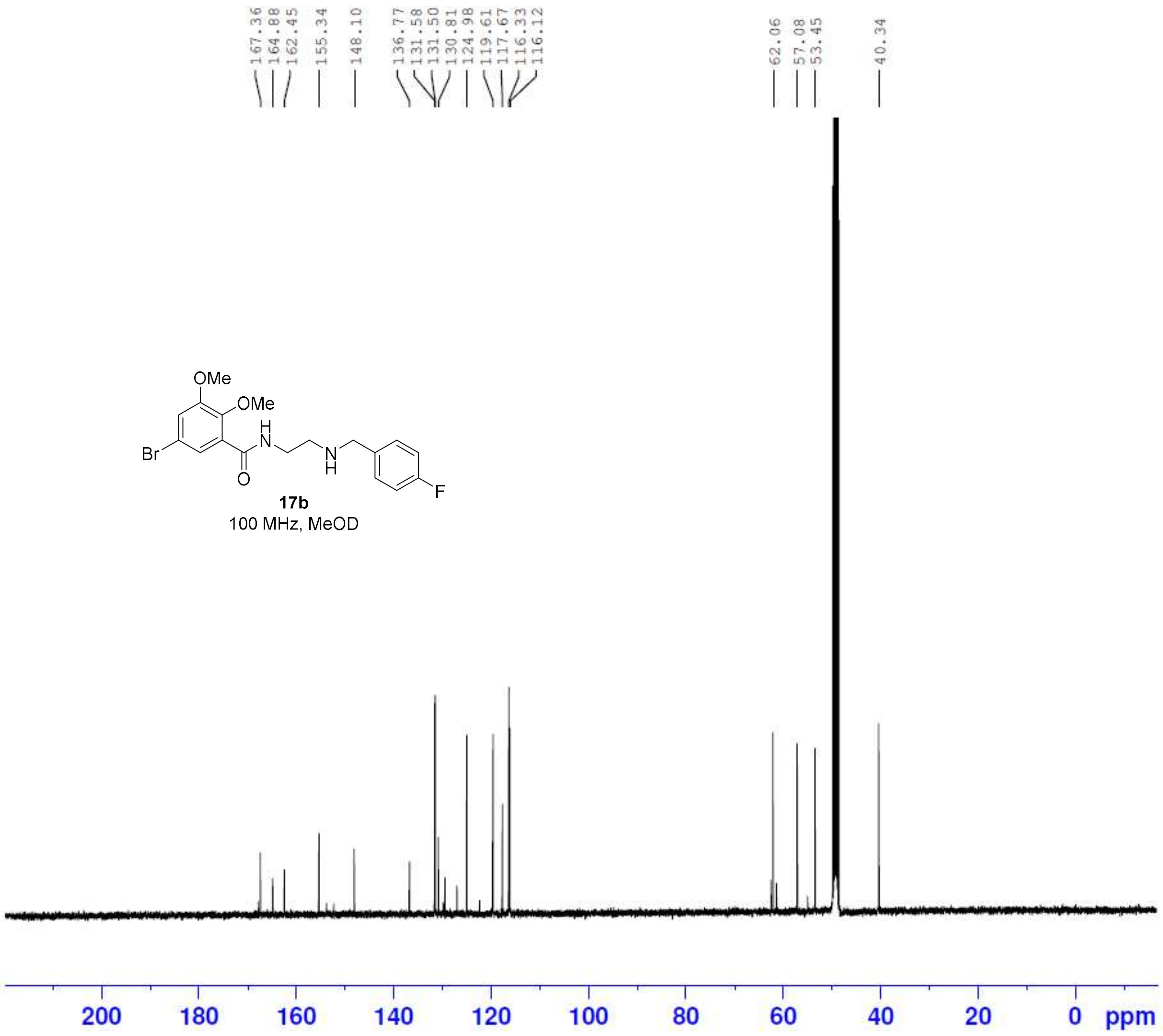
167.36
164.88
162.45
155.34
148.10
136.77
131.58
131.50
130.81
124.98
119.61
117.67
116.33
116.12
62.06
57.08
53.45
40.34
OMe
OMe
H
N
Br
O
N
H
F
17b
100 MHz, MeOD
200
180
160
140
120
100
80
60
40
20
0
ppm

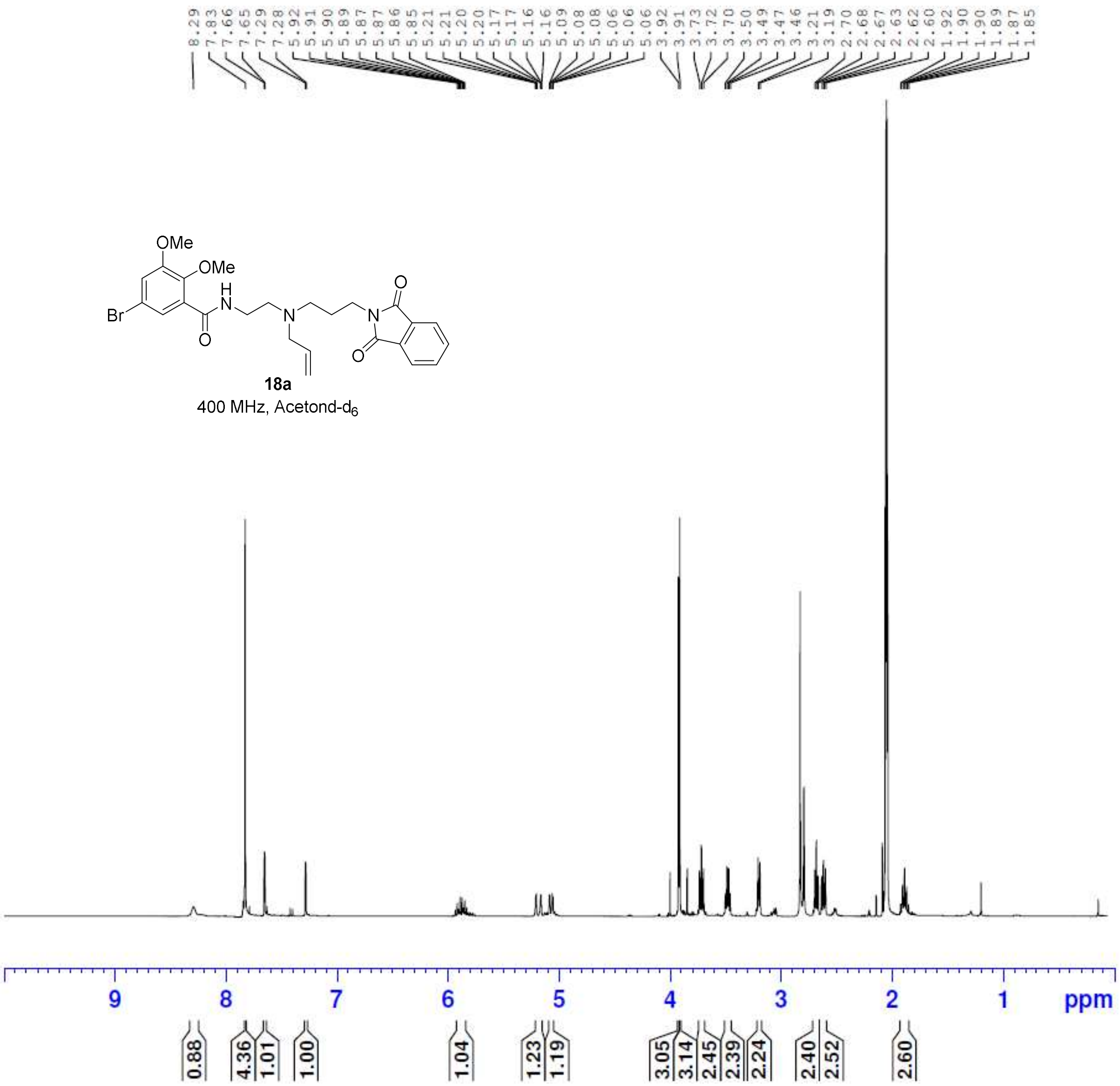
OMe
OMe
H
N
Br
O
N
N
O
O
18a
400 MHz, Acetond-d$_6$
ppm

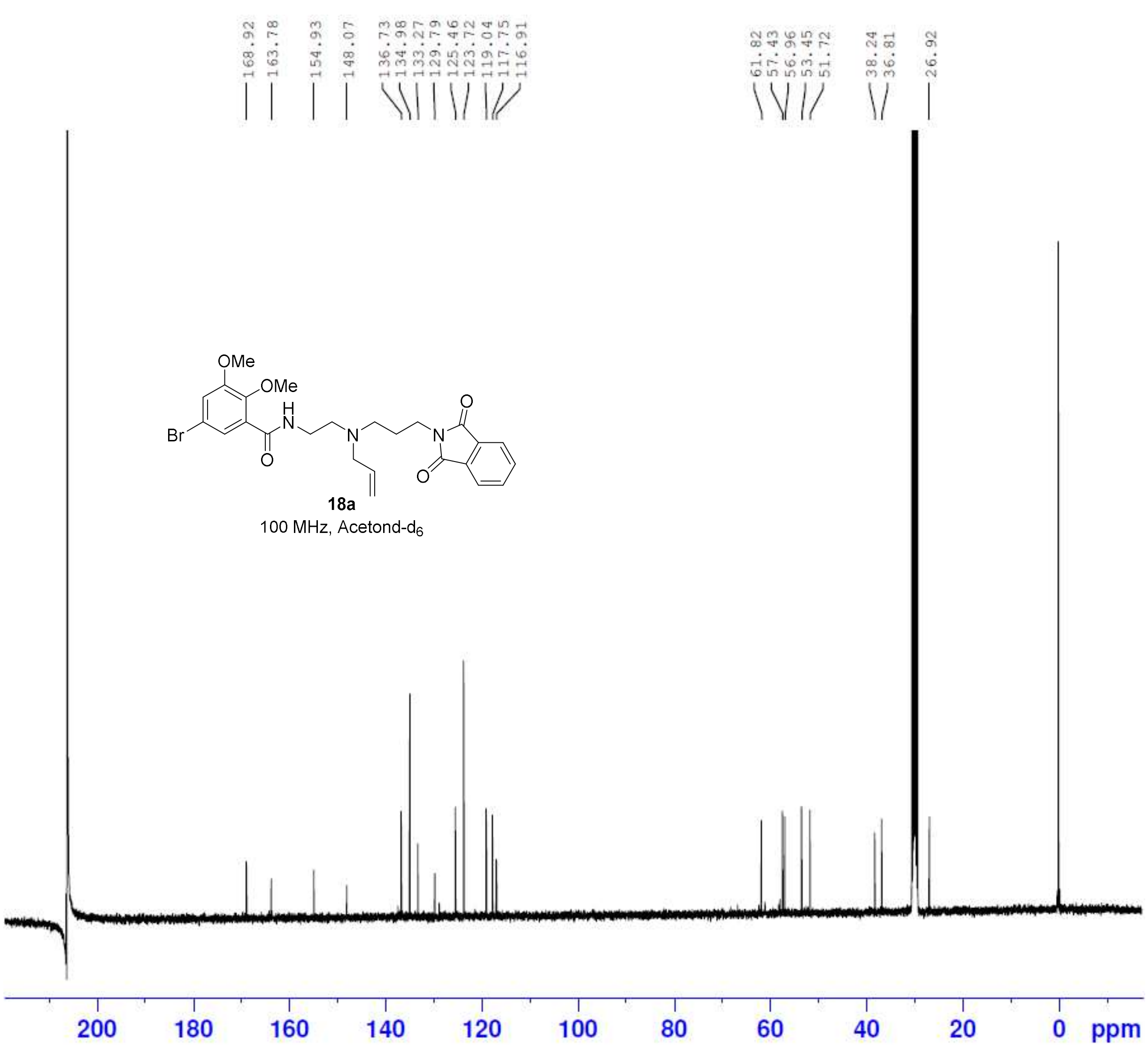
168.92
163.78
154.93
148.07
136.73
134.98
133.27
129.79
125.46
123.72
119.04
117.75
116.91
61.82
57.43
56.96
53.45
51.72
38.24
36.81
26.92
OMe
OMe
H
N
Br
O
N
N
O
O
18a
100 MHz, Acetond-d6
200
180
160
140
120
100
80
60
40
20
0
ppm

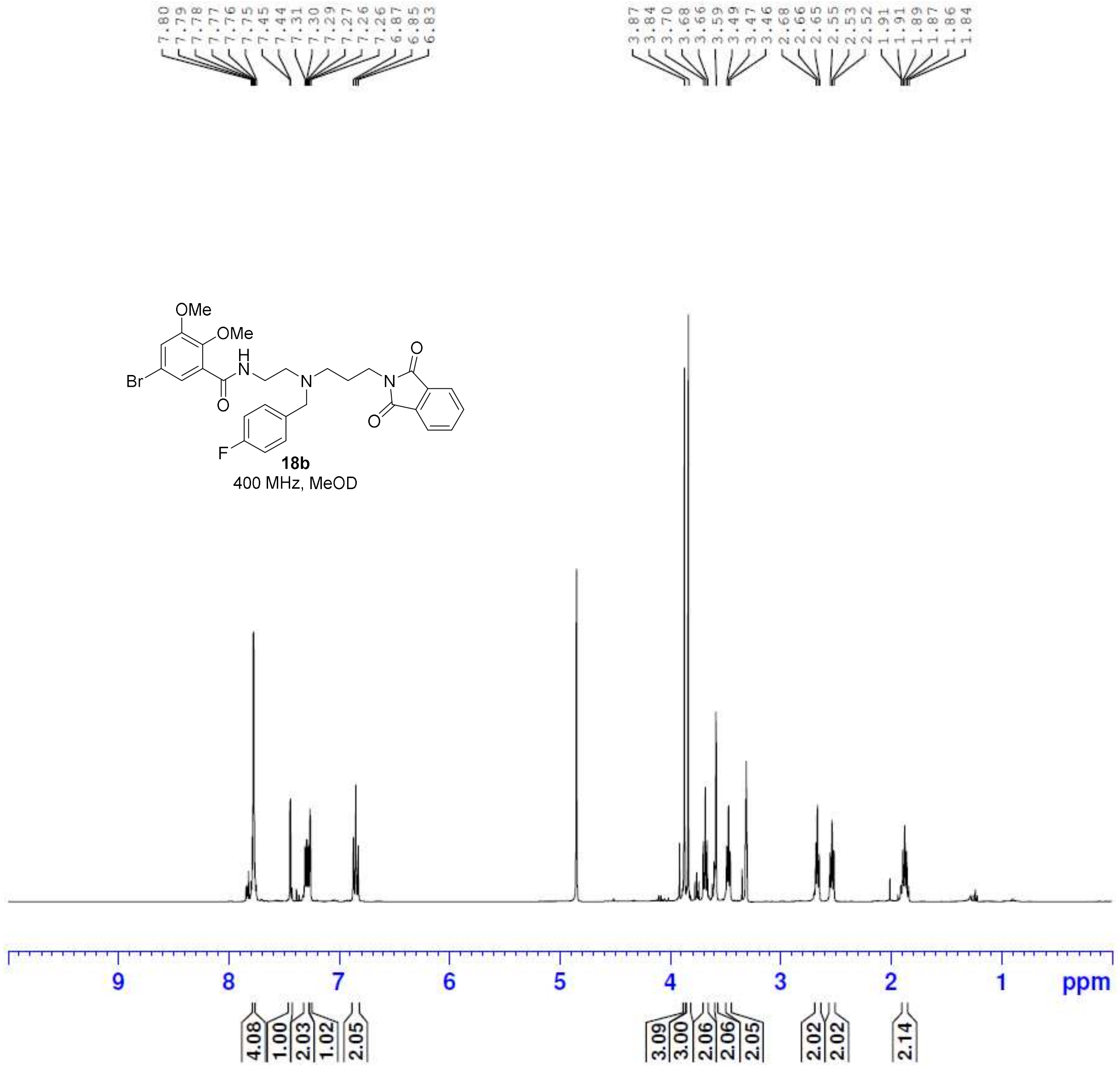
7.80 7.79 7.78 7.77 7.76 7.75 7.45 7.44 7.31 7.30 7.29 7.27 7.26 7.26 6.87 6.85 6.83
3.87 3.84 3.70 3.68 3.66 3.59 3.49 3.47 3.46 2.68 2.66 2.65 2.55 2.53 2.52 1.91 1.91 1.89 1.87 1.86 1.84
OMe
OMe
H
N
Br
O
N
N
O
O
F
18b
400 MHz, MeOD
9 8 7 6 5 4 3 2 1 ppm
4.08 1.00 2.03 1.02 2.05
3.09 3.00 2.06 2.06 2.05
2.02 2.02
2.14

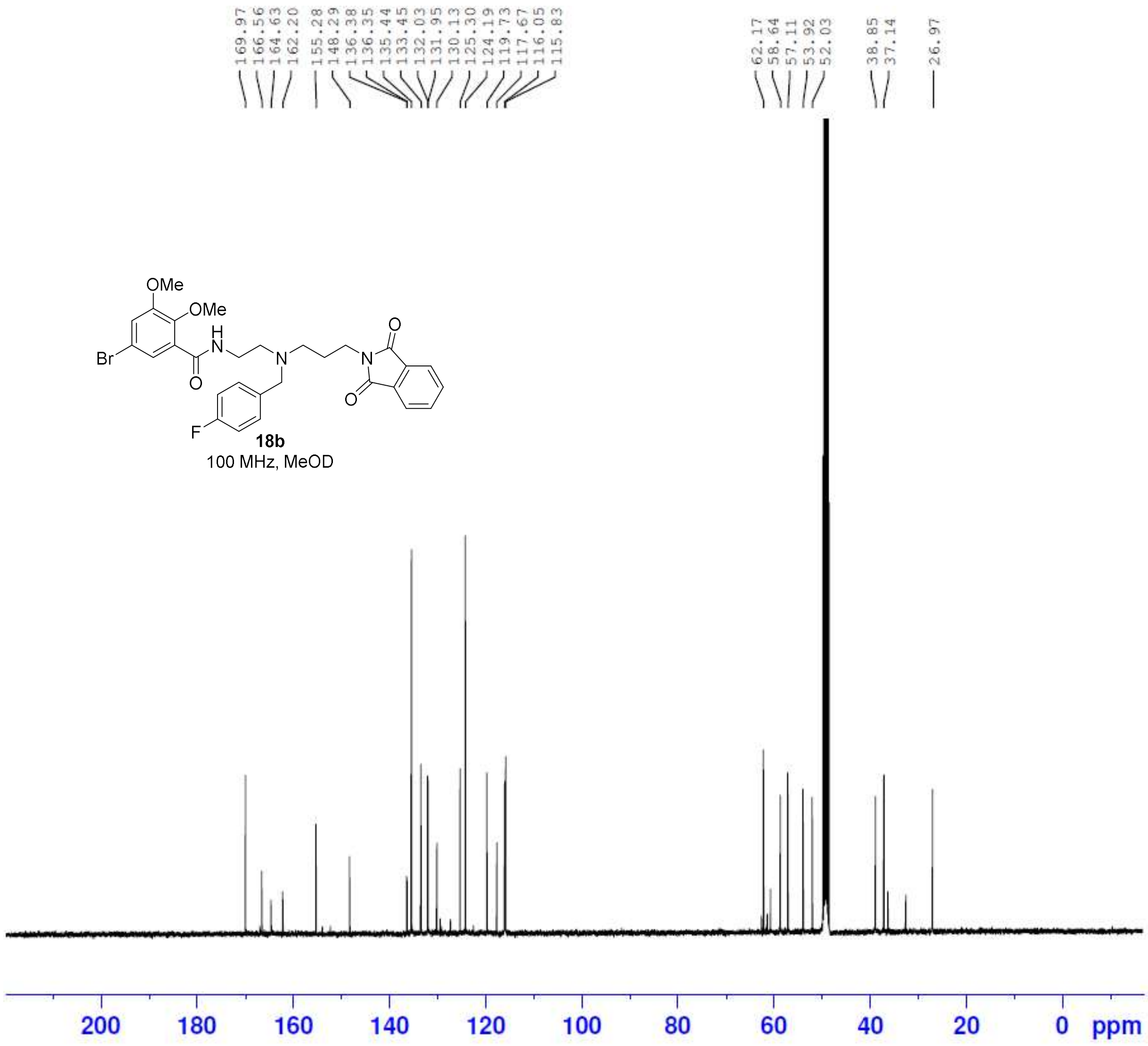
169.97
166.56
164.63
162.20
155.28
148.29
136.38
136.35
135.44
133.45
132.03
131.95
130.13
125.30
124.19
119.73
117.67
116.05
115.83
62.17
58.64
57.11
53.92
52.03
38.85
37.14
26.97
OMe
OMe
Br
H
N
N
N
O
O
O
F
18b
100 MHz, MeOD
200
180
160
140
120
100
80
60
40
20
0
ppm

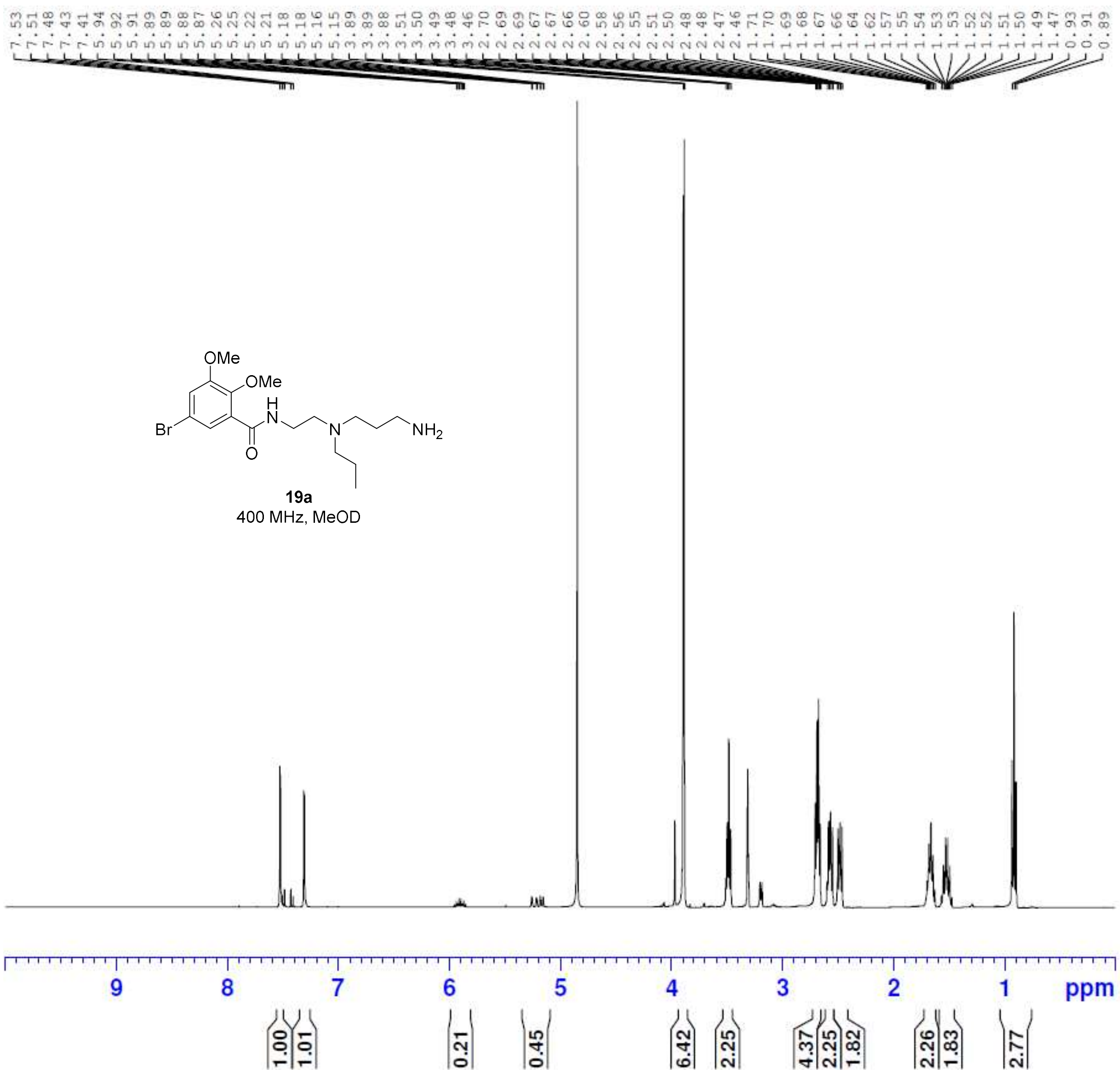
OMe
OMe
H
N
N
Br
O
NH2
19a
400 MHz, MeOD
ppm

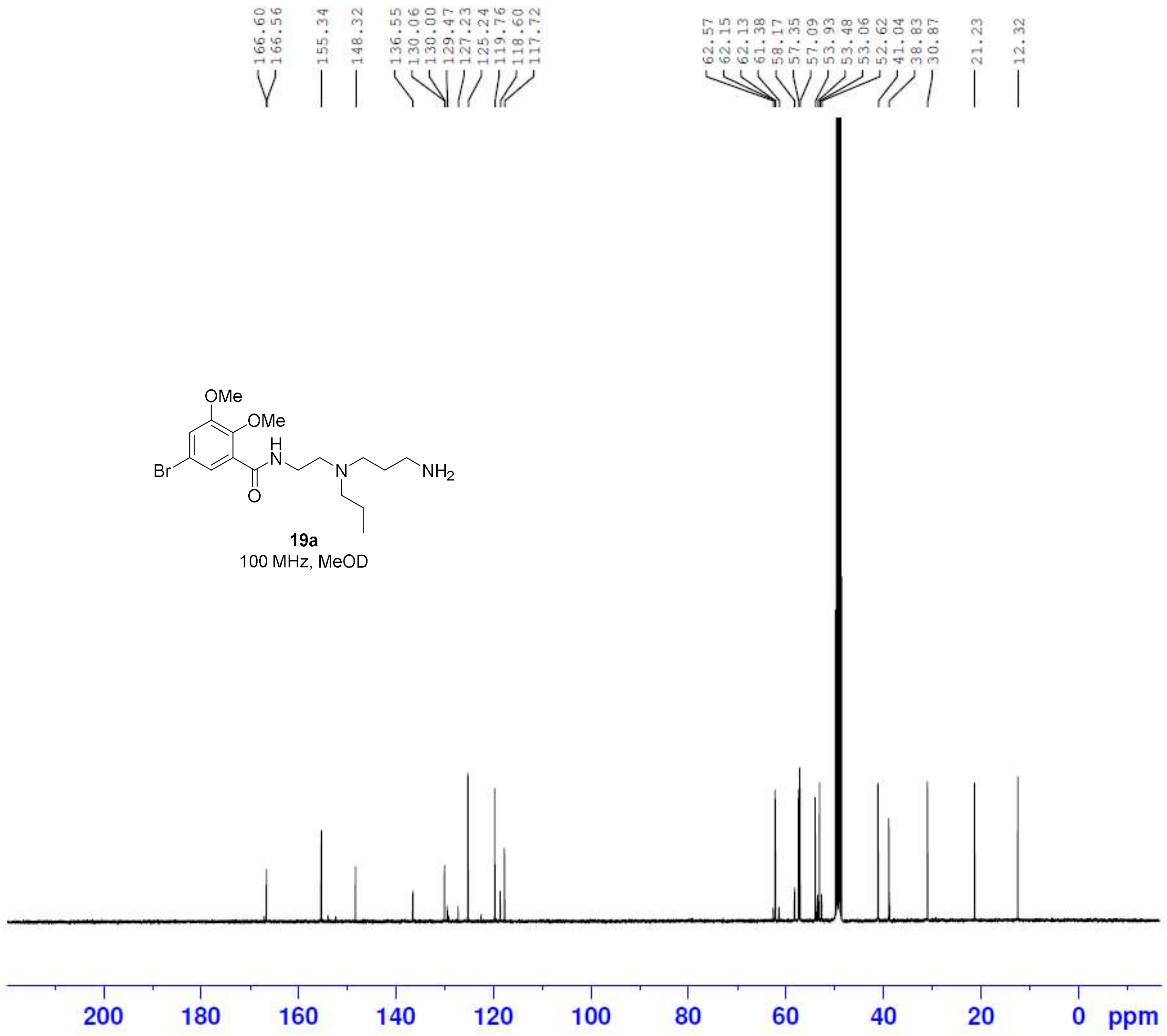

19a

100 MHz, MeOD

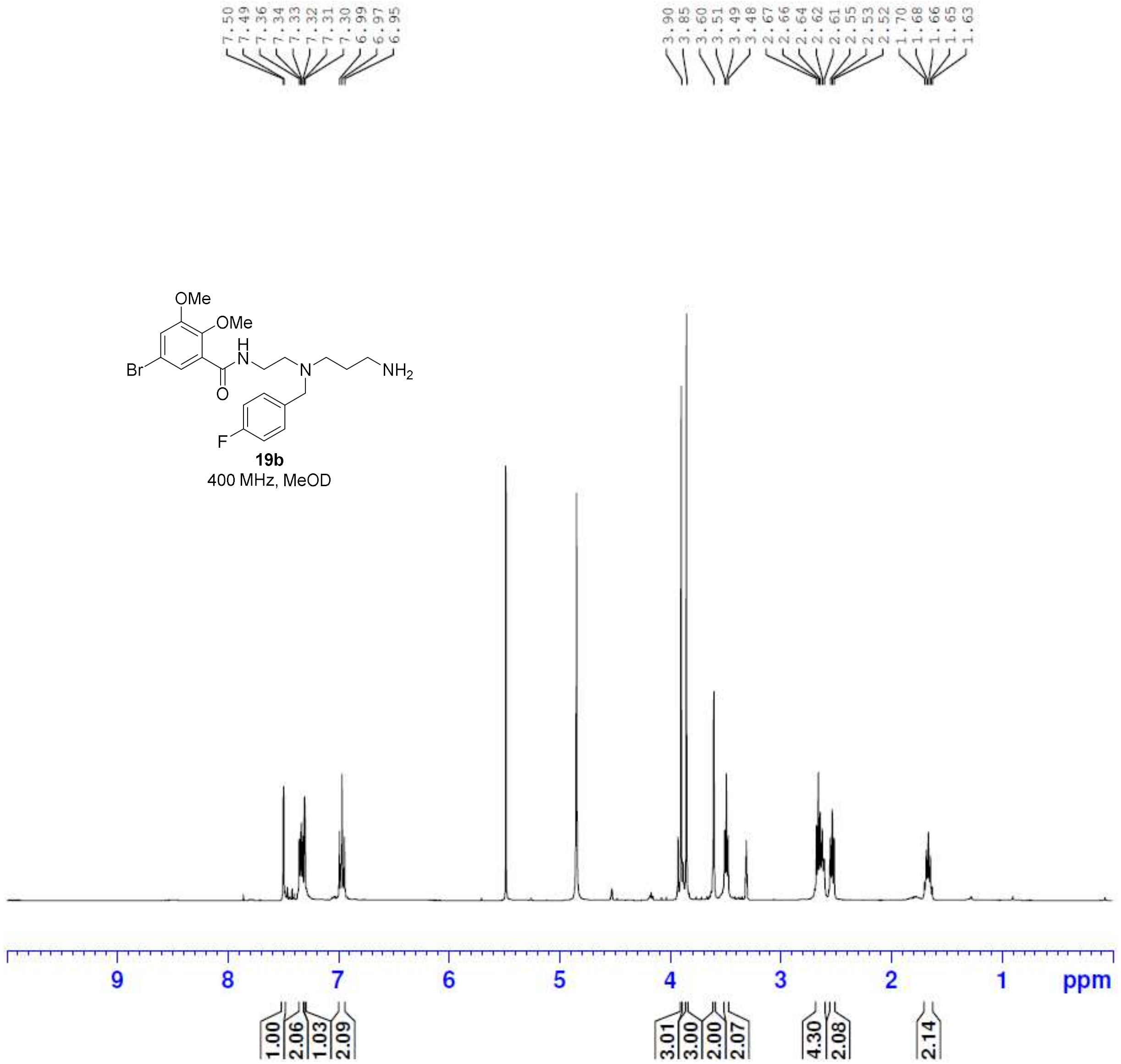
OMe
OMe
H
N
Br
O
N
NH2
F
19b
400 MHz, MeOD
ppm

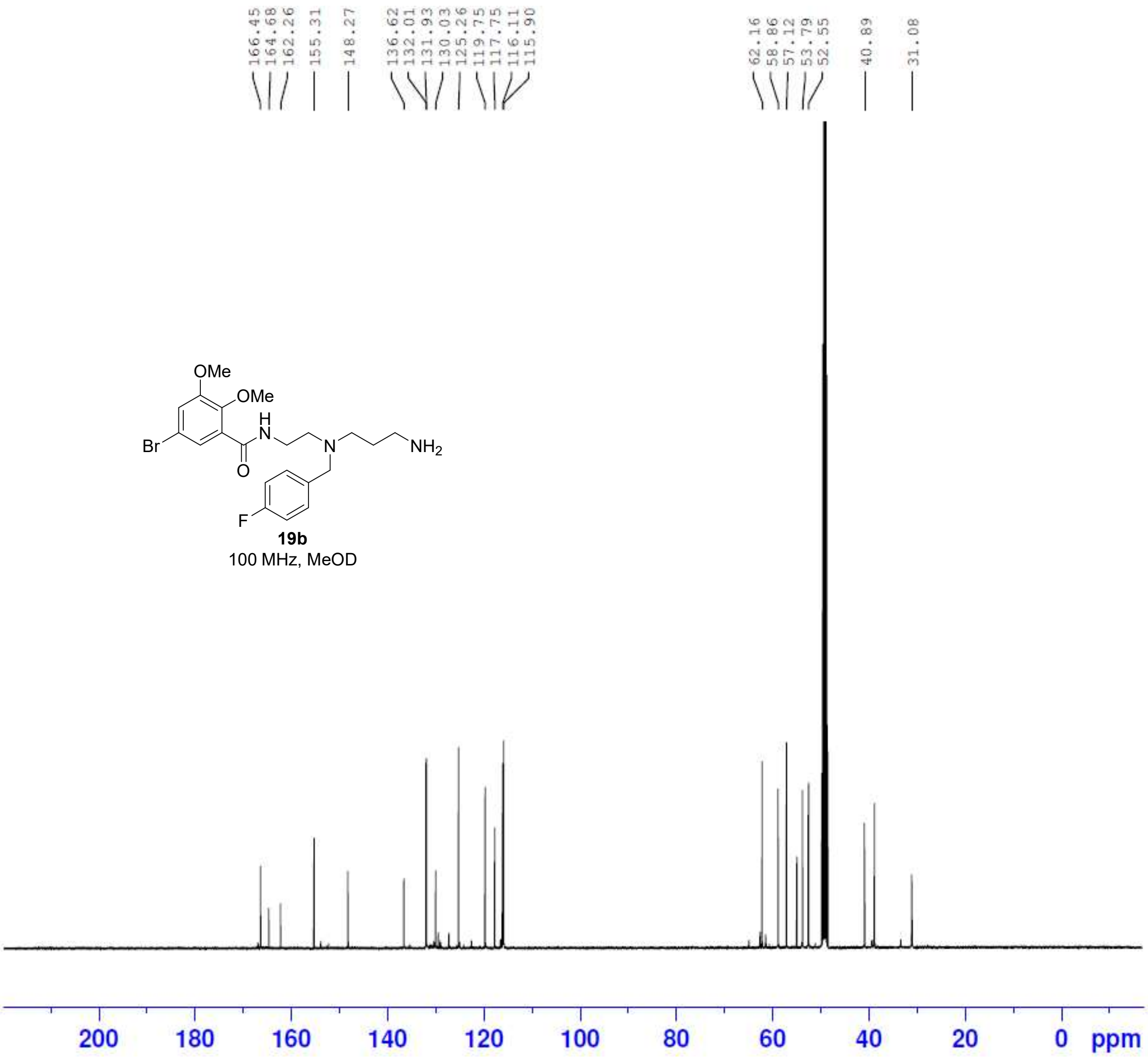
166.45
164.68
162.26
155.31
148.27
136.62
132.01
131.93
130.03
125.26
119.75
117.75
116.11
115.90
62.16
58.86
57.12
53.79
52.55
40.89
31.08
OMe
OMe
H
N
N
NH2
Br
O
F
19b
100 MHz, MeOD
200
180
160
140
120
100
80
60
40
20
0
ppm

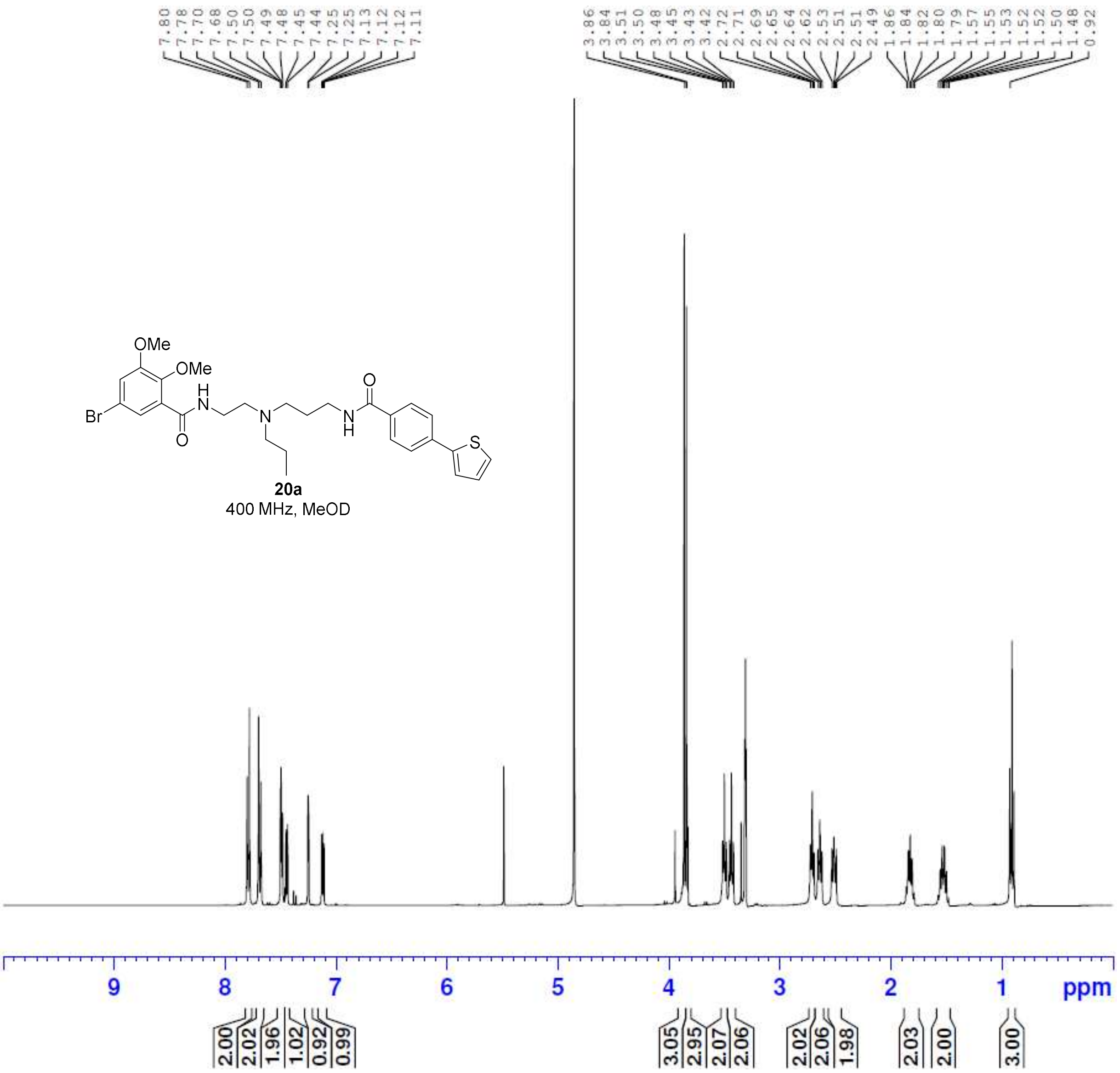

20a
400 MHz, MeOD

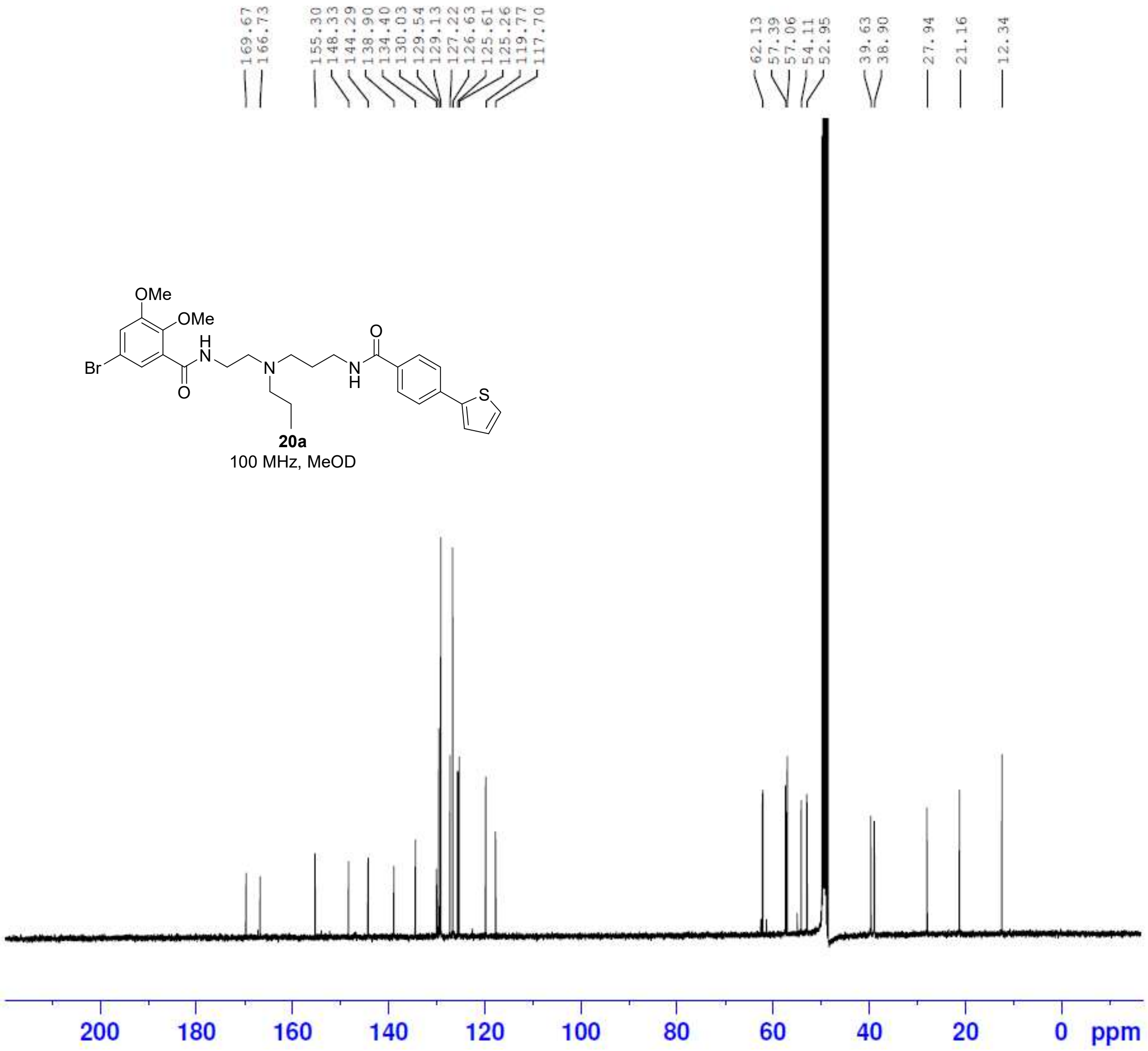
169.67
166.73
155.30
148.33
144.29
138.90
134.40
130.03
129.54
129.13
127.22
126.63
125.61
125.26
119.77
117.70
62.13
57.39
57.06
54.11
52.95
39.63
38.90
27.94
21.16
12.34
OMe
OMe
H
N
N
N
H
O
O
Br
S
20a
100 MHz, MeOD
200
180
160
140
120
100
80
60
40
20
0
ppm

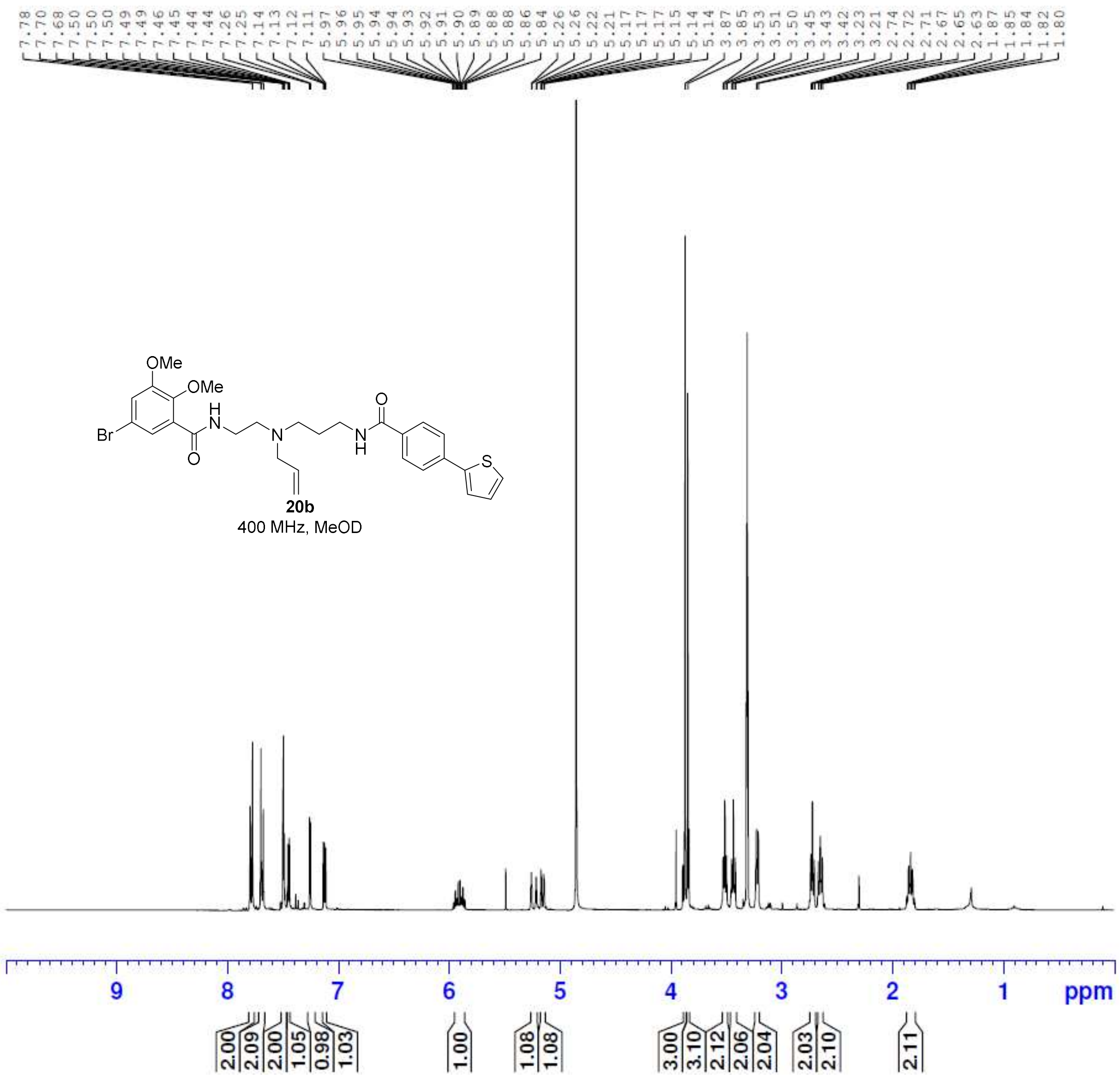

OMe
OMe
Br
H
N
O
S
20b
400 MHz, MeOD
9
8
7
6
5
4
3
2
1
ppm

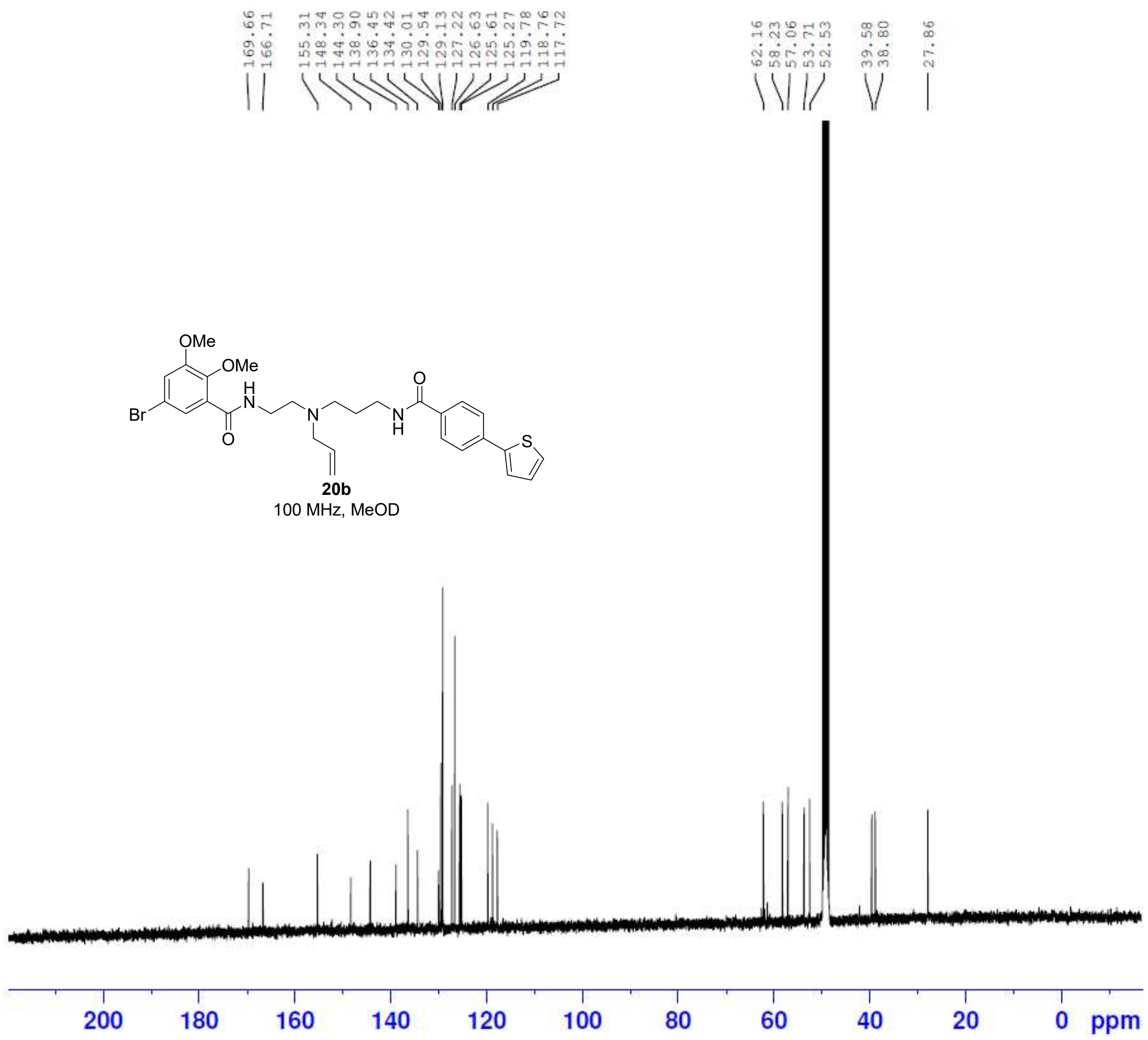
169.66
166.71
155.31
148.34
144.30
138.90
136.45
134.42
130.01
129.54
129.13
127.22
126.63
125.61
125.27
119.78
118.76
117.72
62.16
58.23
57.06
53.71
52.53
39.58
38.80
27.86
OMe
OMe
H
N
Br
O
N
O
N
H
S
20b
100 MHz, MeOD
200
180
160
140
120
100
80
60
40
20
0
ppm

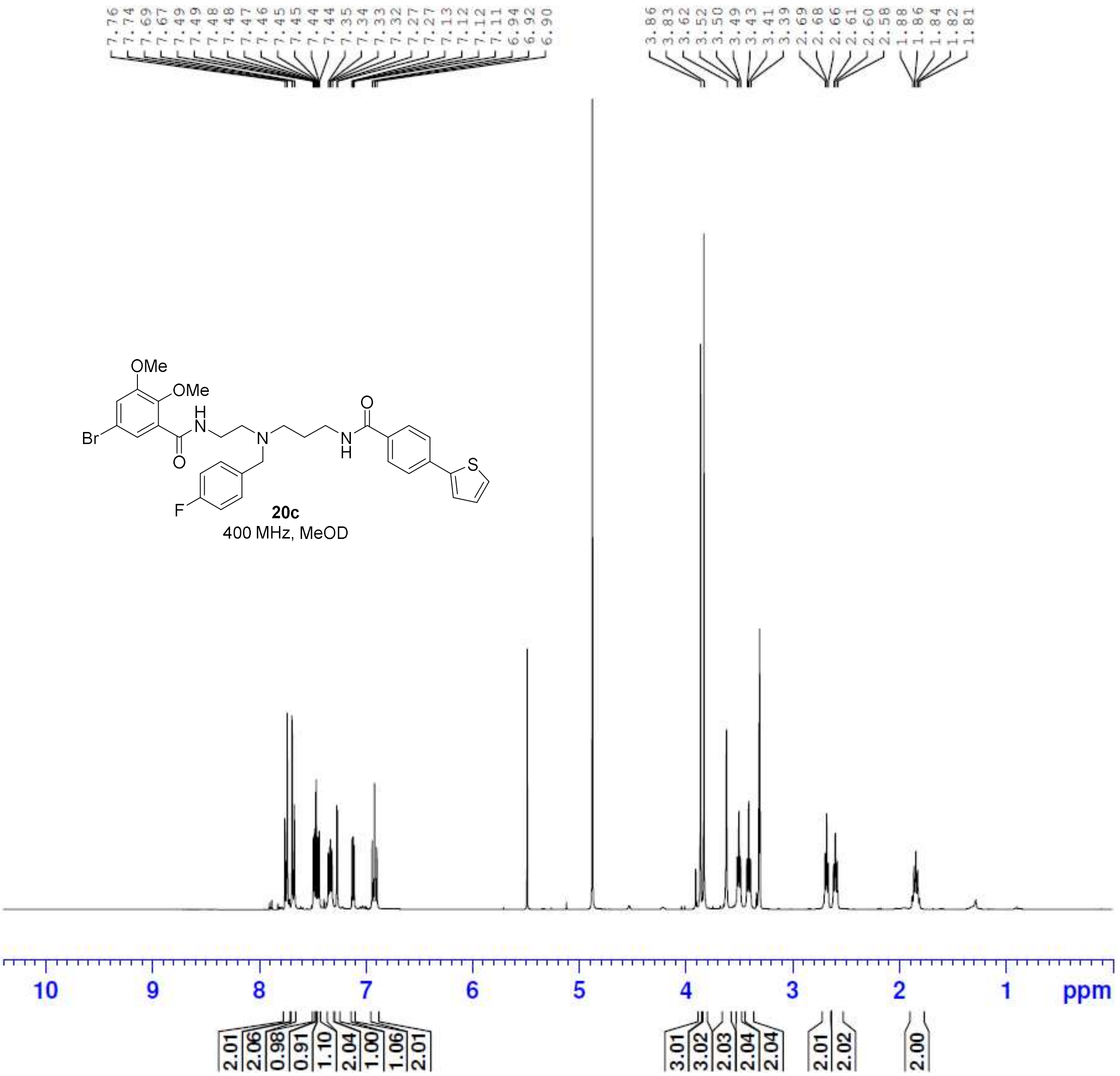

7.76
7.74
7.69
7.67
7.49
7.49
7.48
7.48
7.47
7.46
7.45
7.45
7.44
7.44
7.35
7.34
7.33
7.32
7.27
7.27
7.13
7.12
7.12
7.11
6.94
6.92
6.90
3.86
3.83
3.62
3.52
3.50
3.49
3.43
3.41
3.39
2.69
2.68
2.66
2.61
2.60
2.58
1.88
1.86
1.84
1.82
1.81
OMe
OMe
Br
H
N
N
O
O
N
H
S
F
20c
400 MHz, MeOD
10
9
8
7
6
5
4
3
2
1
ppm
2.01
2.06
0.98
0.91
1.10
2.04
1.00
1.06
2.01
3.01
3.02
2.03
2.04
2.04
2.01
2.02
2.00

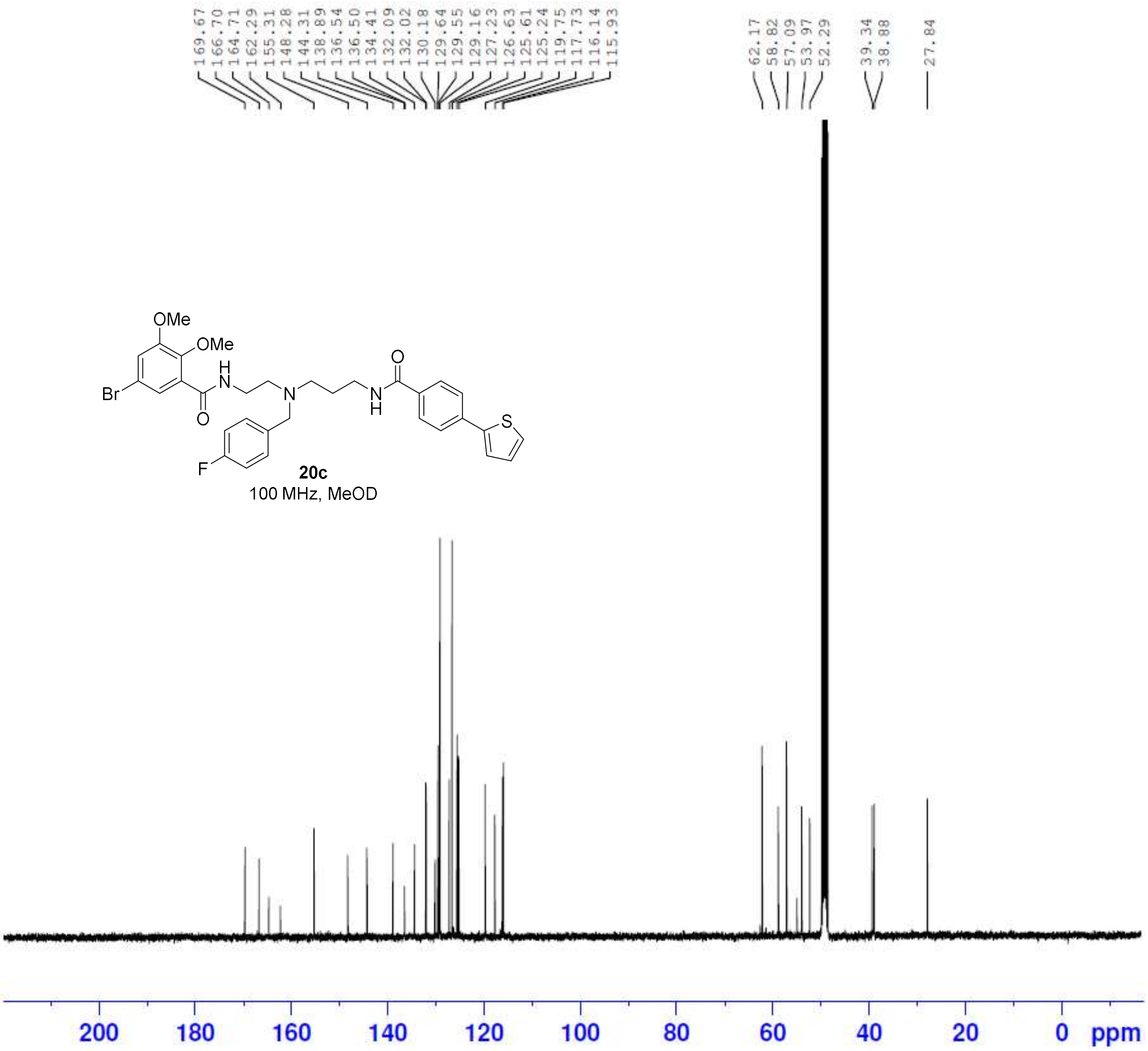

169.67
166.70
164.71
162.29
155.31
148.28
144.31
138.89
136.54
136.50
134.41
132.09
132.02
130.18
129.64
129.55
129.16
127.23
126.63
125.61
125.24
119.75
117.73
116.14
115.93
62.17
58.82
57.09
53.97
52.29
39.34
38.88
27.84
OMe
OMe
Br
N
H
O
N
H
N
O
S
F
20c
100 MHz, MeOD
200
180
160
140
120
100
80
60
40
20
0
ppm

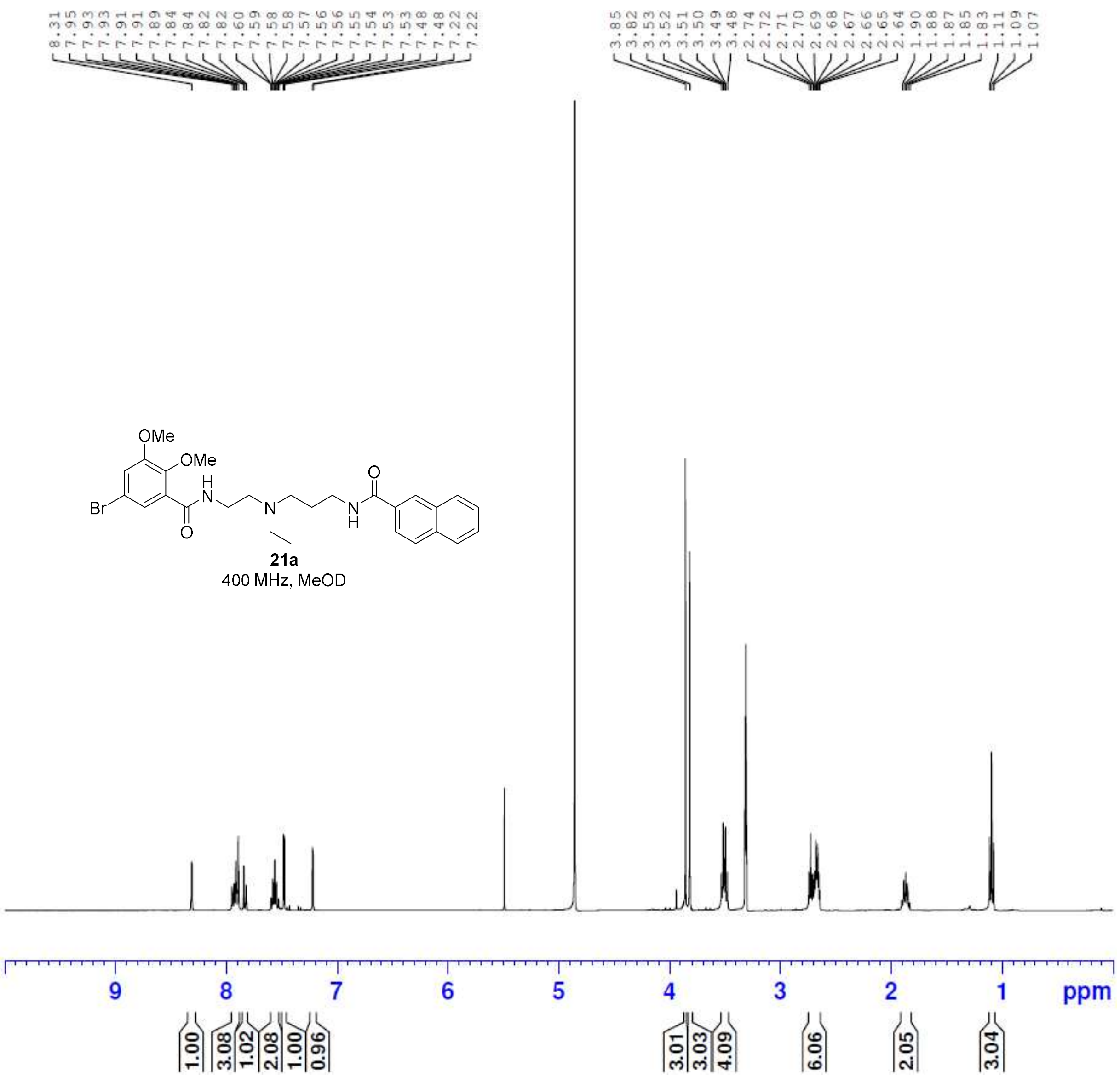
8.31 7.95 7.93 7.93 7.91 7.91 7.89 7.84 7.82 7.82 7.60 7.59 7.58 7.58 7.57 7.56 7.55 7.54 7.53 7.53 7.48 7.48 7.22 7.22
3.85 3.82 3.53 3.52 3.51 3.50 3.49 3.48 2.74 2.72 2.71 2.70 2.69 2.68 2.67 2.66 2.65 2.64 1.90 1.88 1.87 1.85 1.83 1.11 1.09 1.07
OMe
OMe
Br
H
N
N
O
O
N
H
21a
400 MHz, MeOD
9 8 7 6 5 4 3 2 1 ppm
1.00 3.08 1.02 2.08 1.00 0.96
3.01 3.03 4.09
6.06
2.05
3.04

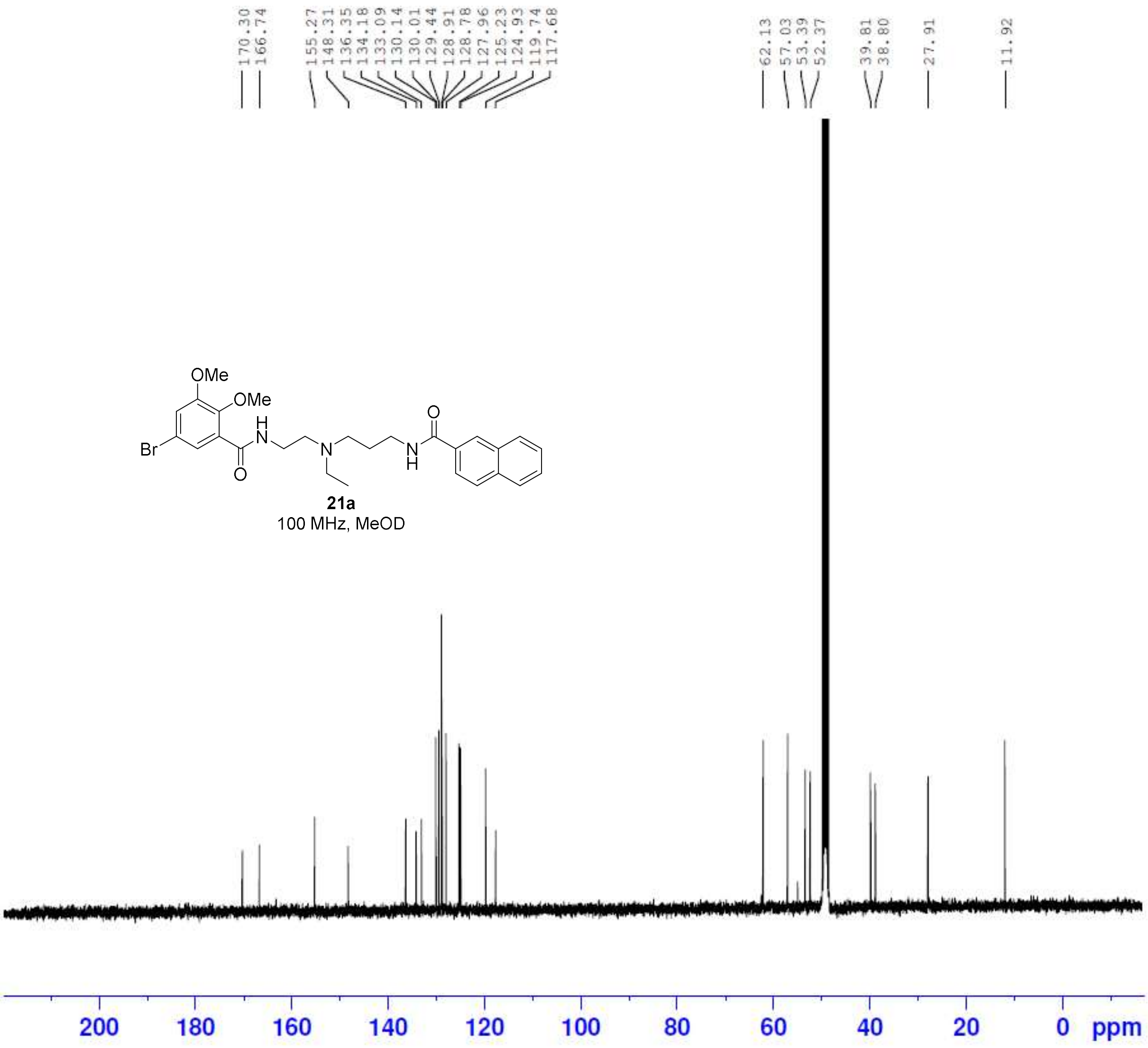

21a

100 MHz, MeOD

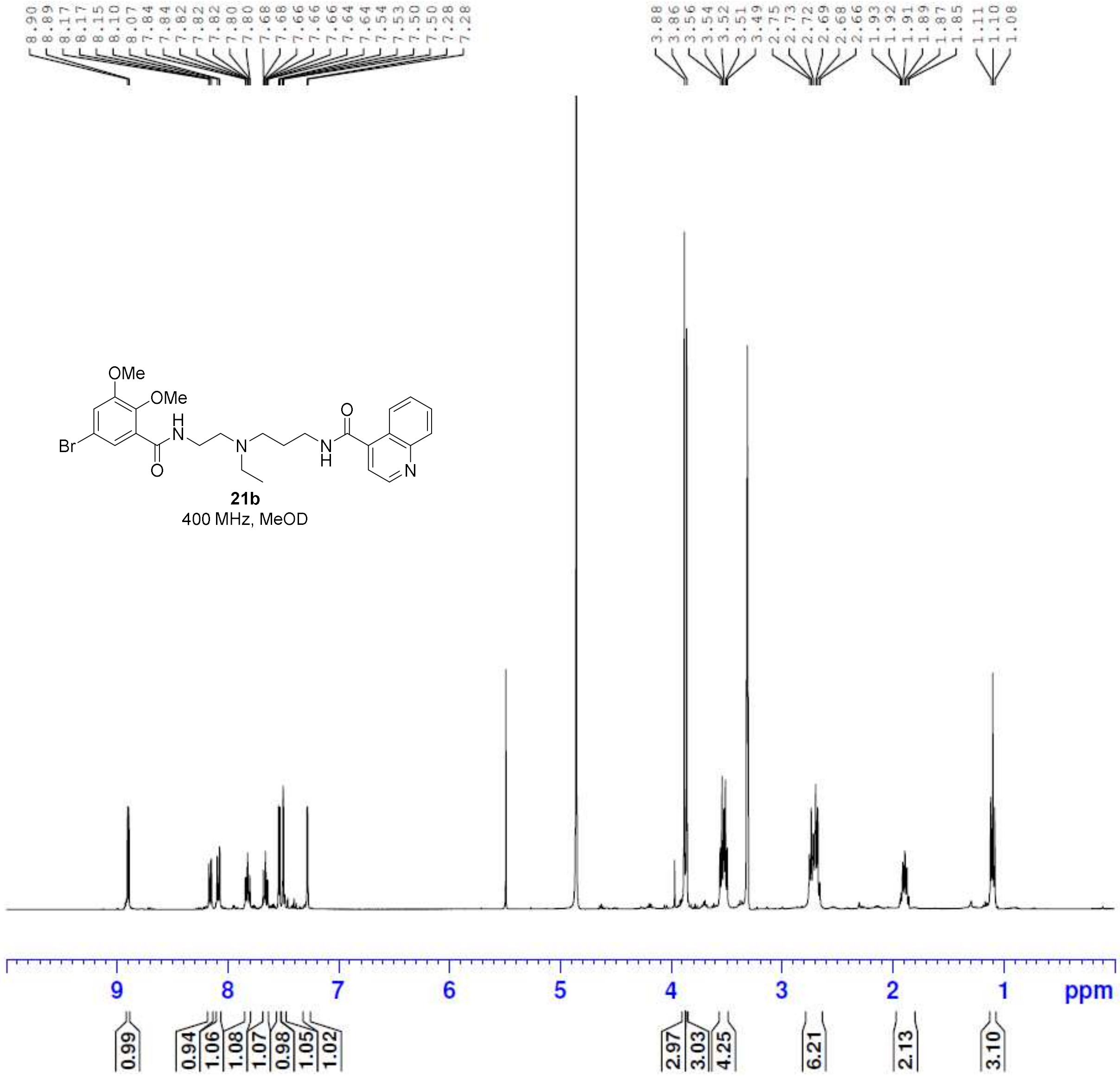

21b
400 MHz, MeOD

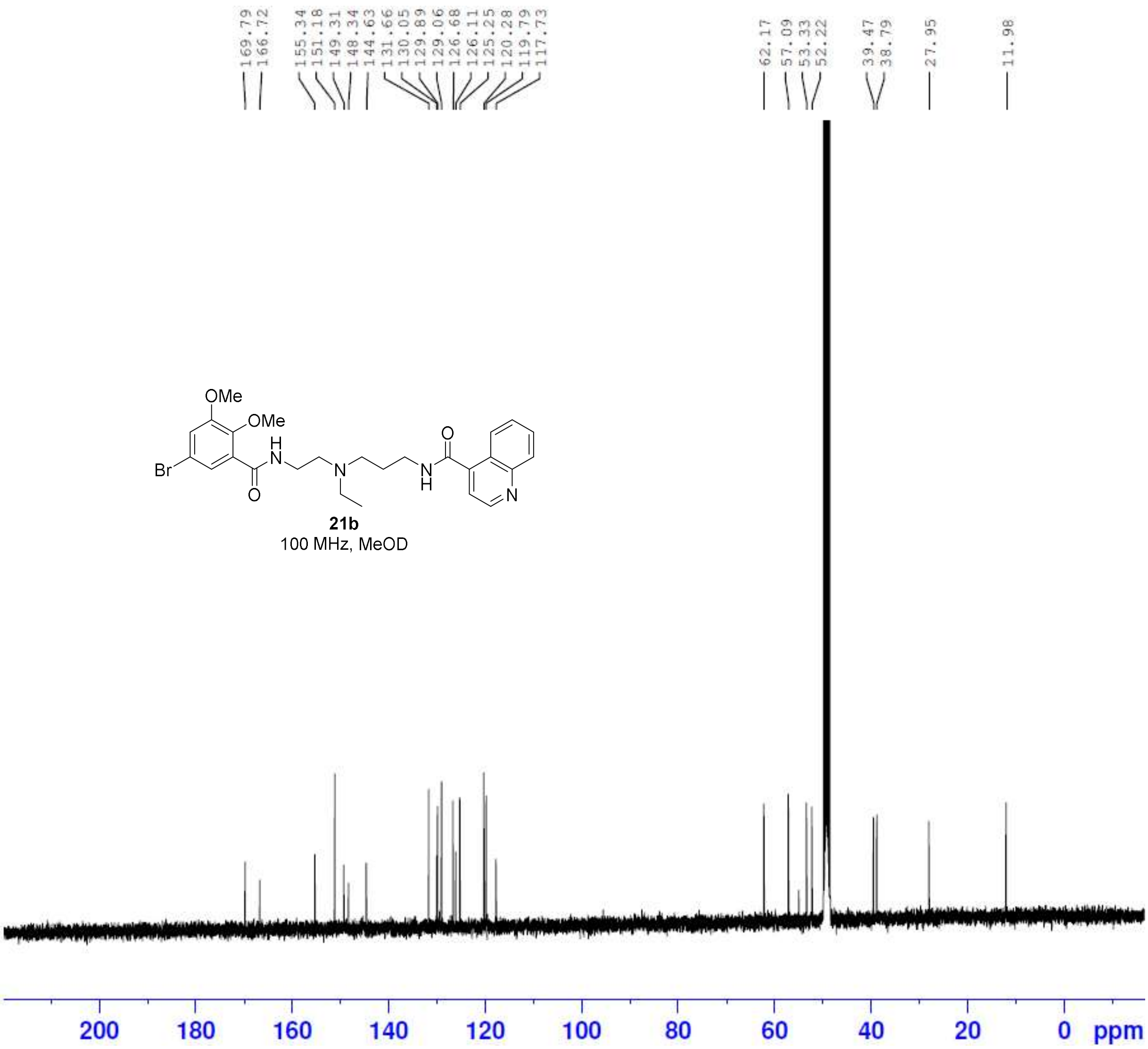

21b
100 MHz, MeOD

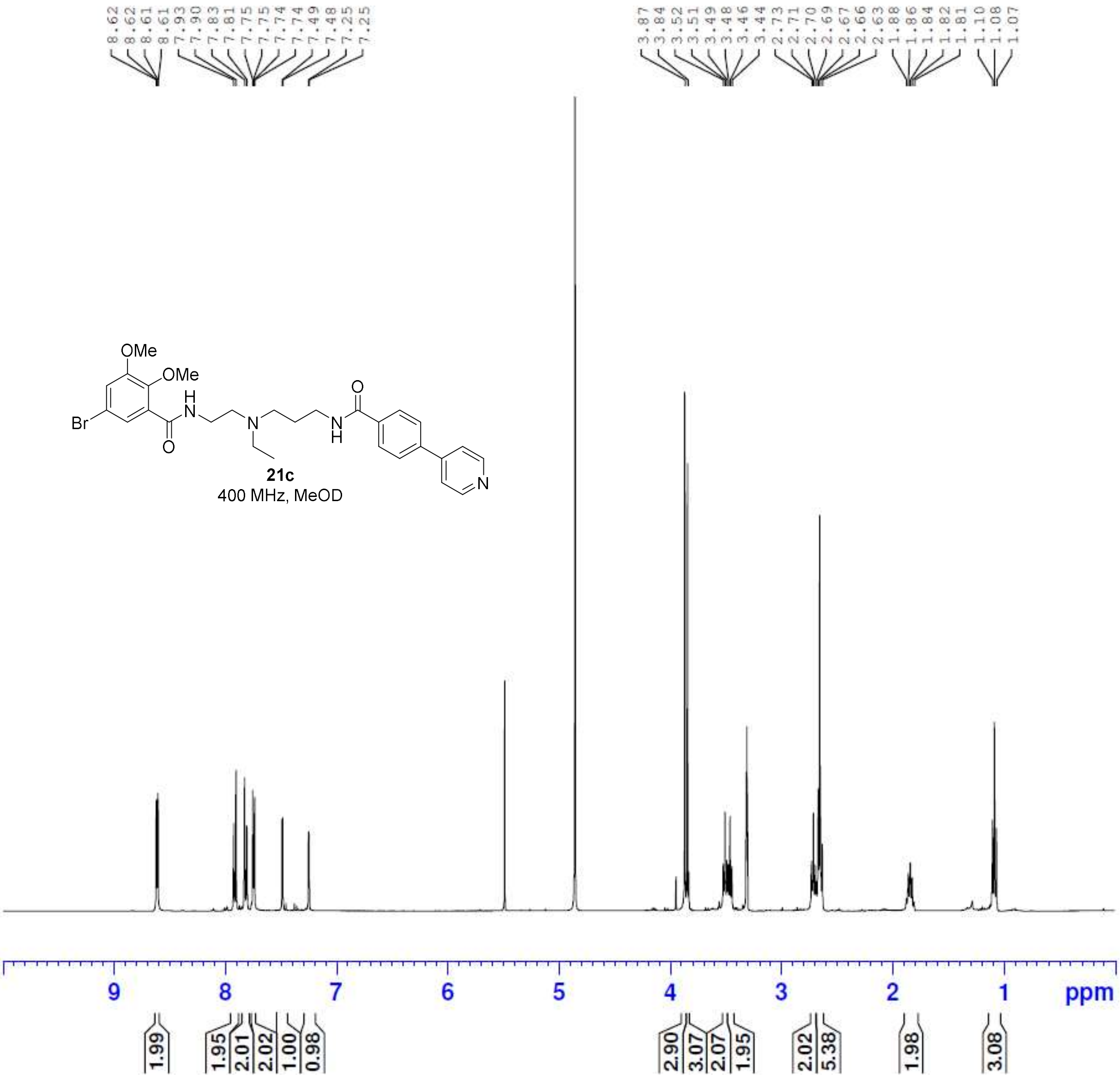
8.62
8.62
8.61
8.61
7.93
7.90
7.83
7.81
7.75
7.75
7.74
7.74
7.49
7.48
7.25
7.25
3.87
3.84
3.52
3.51
3.49
3.48
3.46
3.44
2.73
2.71
2.70
2.69
2.67
2.66
2.63
1.88
1.86
1.84
1.82
1.81
1.10
1.08
1.07
OMe
OMe
Br
H
N
N
H
N
O
O
N
21c
400 MHz, MeOD
9
8
7
6
5
4
3
2
1
ppm
1.99
1.95
2.01
2.02
1.00
0.98
2.90
3.07
2.07
1.95
2.02
5.38
1.98
3.08

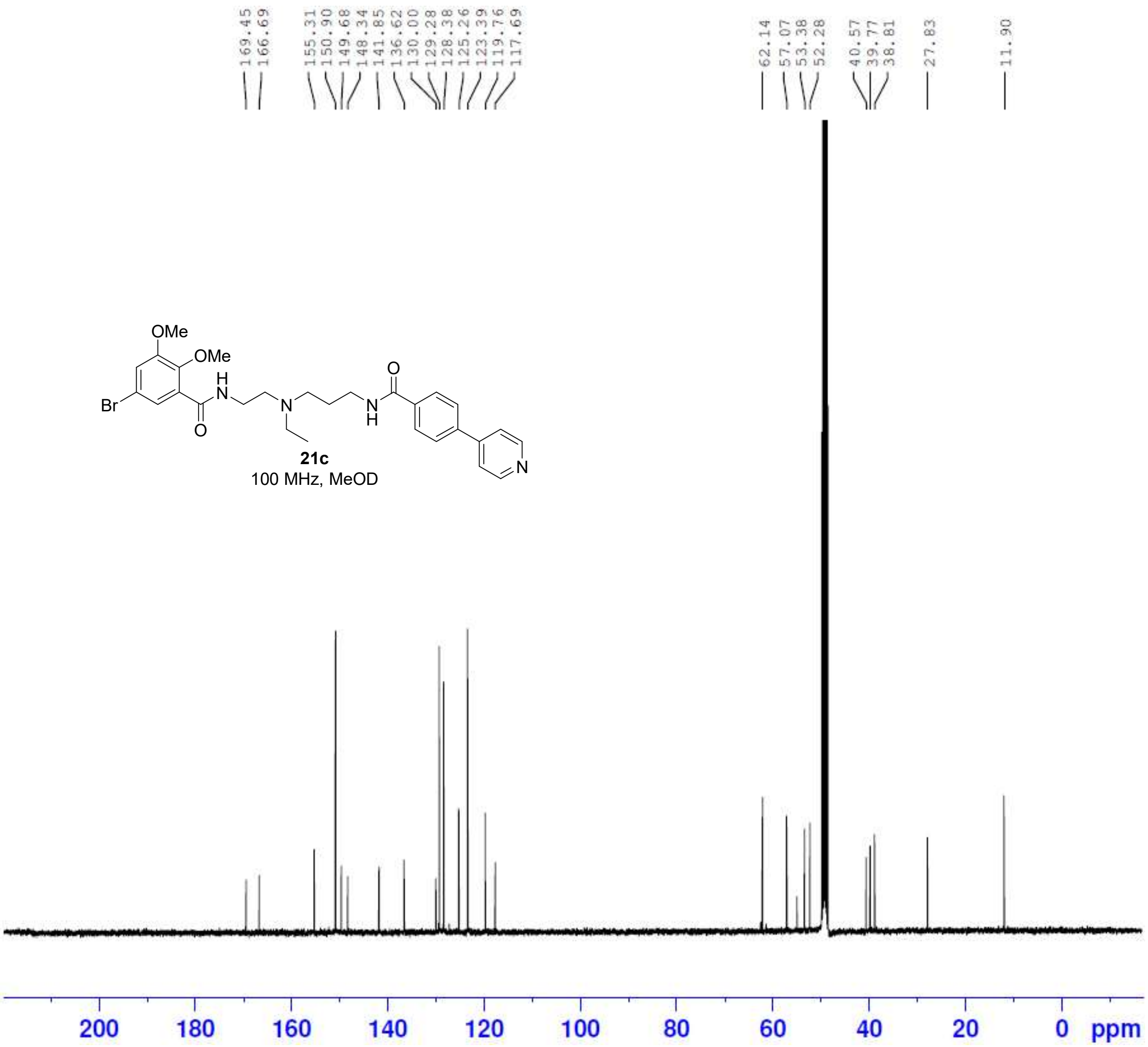
169.45
166.69
155.31
150.90
149.68
148.34
141.85
136.62
130.00
129.28
128.38
125.26
123.39
119.76
117.69
62.14
57.07
53.38
52.28
40.57
39.77
38.81
27.83
11.90
OMe
OMe
Br
H
N
N
O
N
H
O
N
21c
100 MHz, MeOD
200
180
160
140
120
100
80
60
40
20
0
ppm

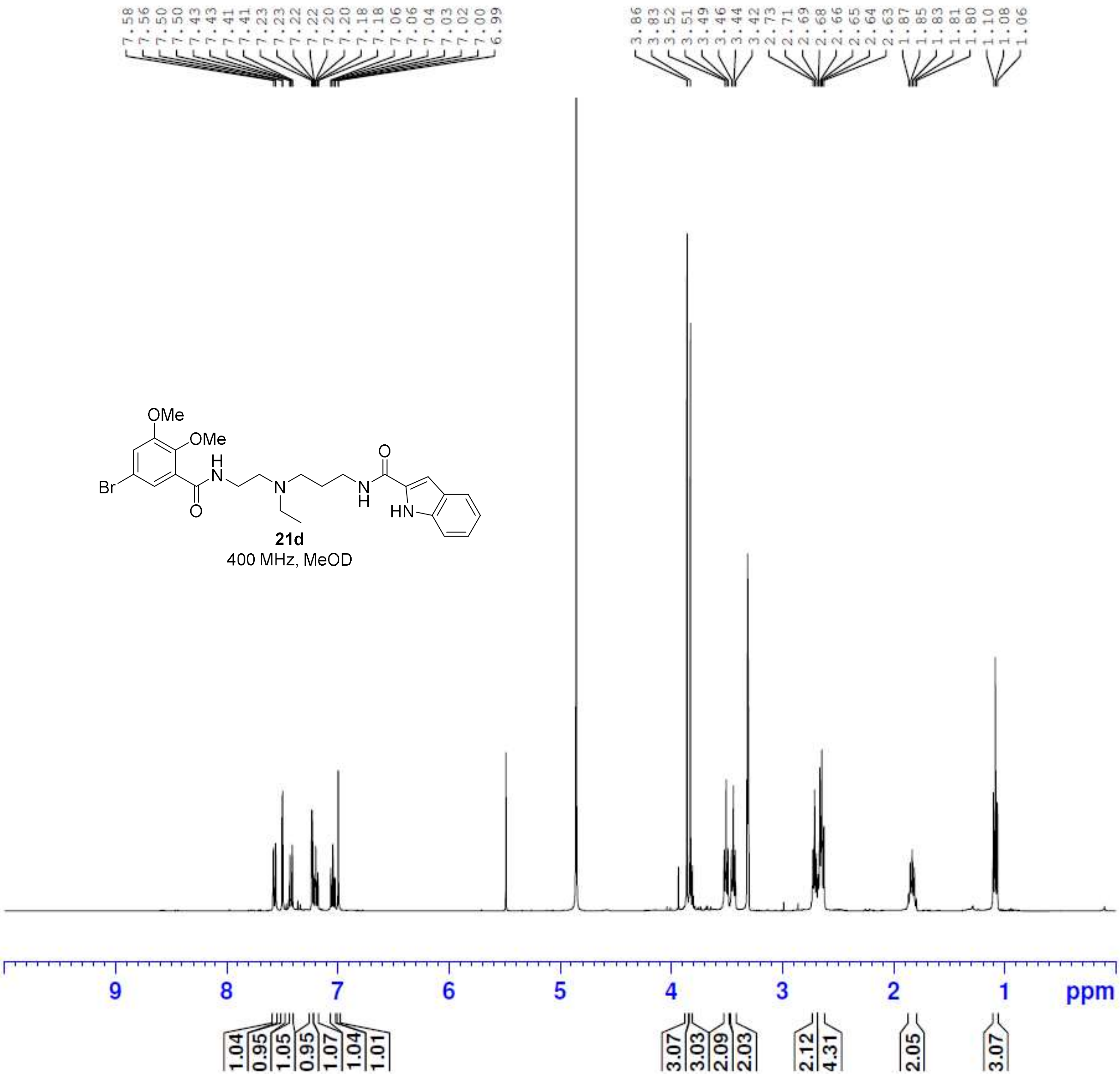
7.58 7.56 7.50 7.50 7.43 7.43 7.41 7.41 7.23 7.23 7.22 7.22 7.20 7.20 7.18 7.18 7.06 7.06 7.04 7.03 7.02 7.00 6.99
3.86 3.83 3.52 3.51 3.49 3.46 3.44 3.42 2.73 2.71 2.69 2.68 2.66 2.65 2.64 2.63 1.87 1.85 1.83 1.81 1.80 1.10 1.08 1.06
OMe
OMe
Br
H
N
N
O
H
N
O
HN
21d
400 MHz, MeOD
9 8 7 6 5 4 3 2 1 ppm
1.04 0.95 1.05 0.95 1.07 1.04 1.01
3.07 3.03 2.09 2.03
2.12 4.31
2.05
3.07

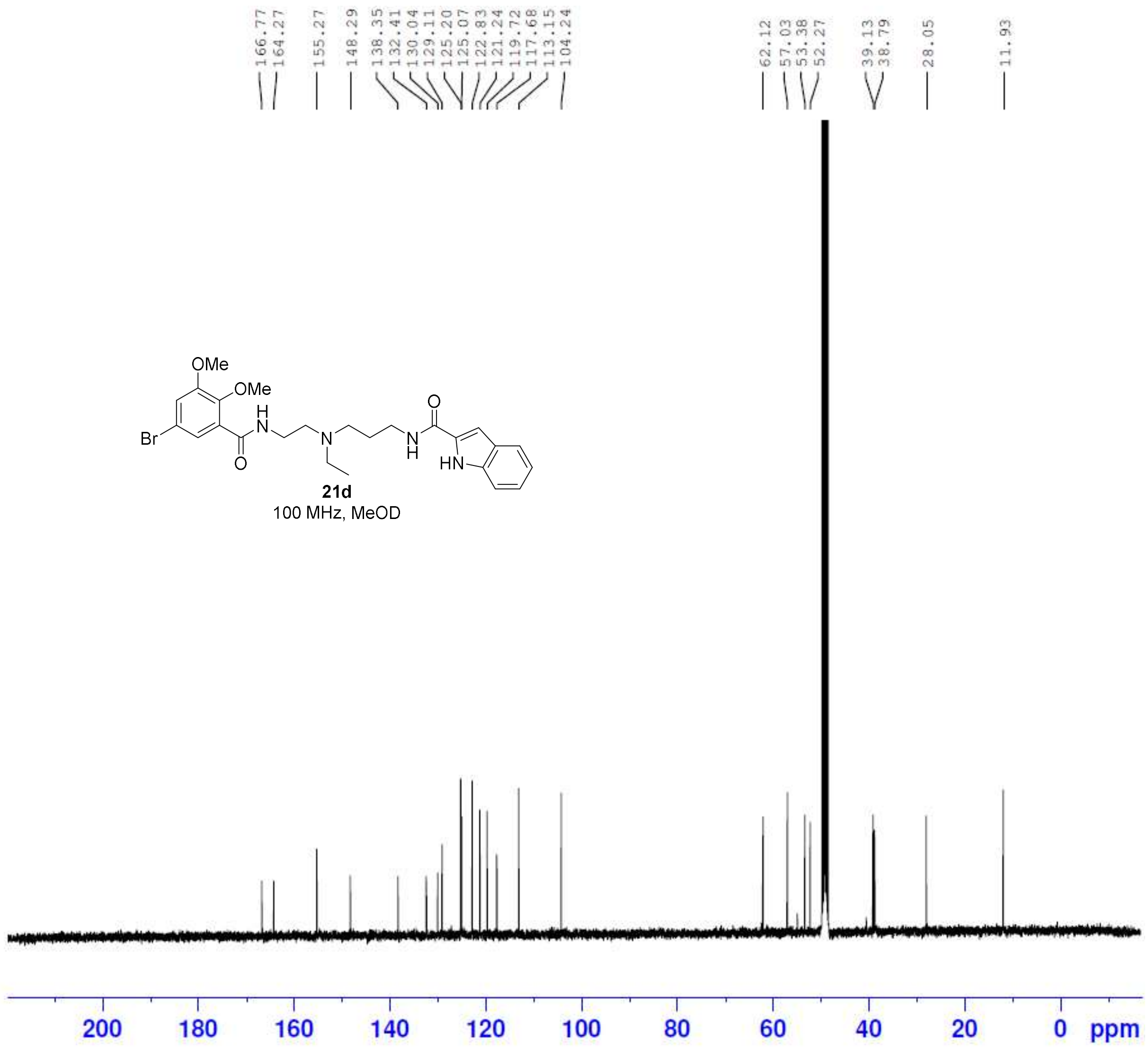

166.77
164.27
155.27
148.29
138.35
132.41
130.04
129.11
125.20
125.07
122.83
121.24
119.72
117.68
113.15
104.24
62.12
57.03
53.38
52.27
39.13
38.79
28.05
11.93
OMe
OMe
Br
H
N
N
O
N
H
O
HN
21d
100 MHz, MeOD
200
180
160
140
120
100
80
60
40
20
0
ppm

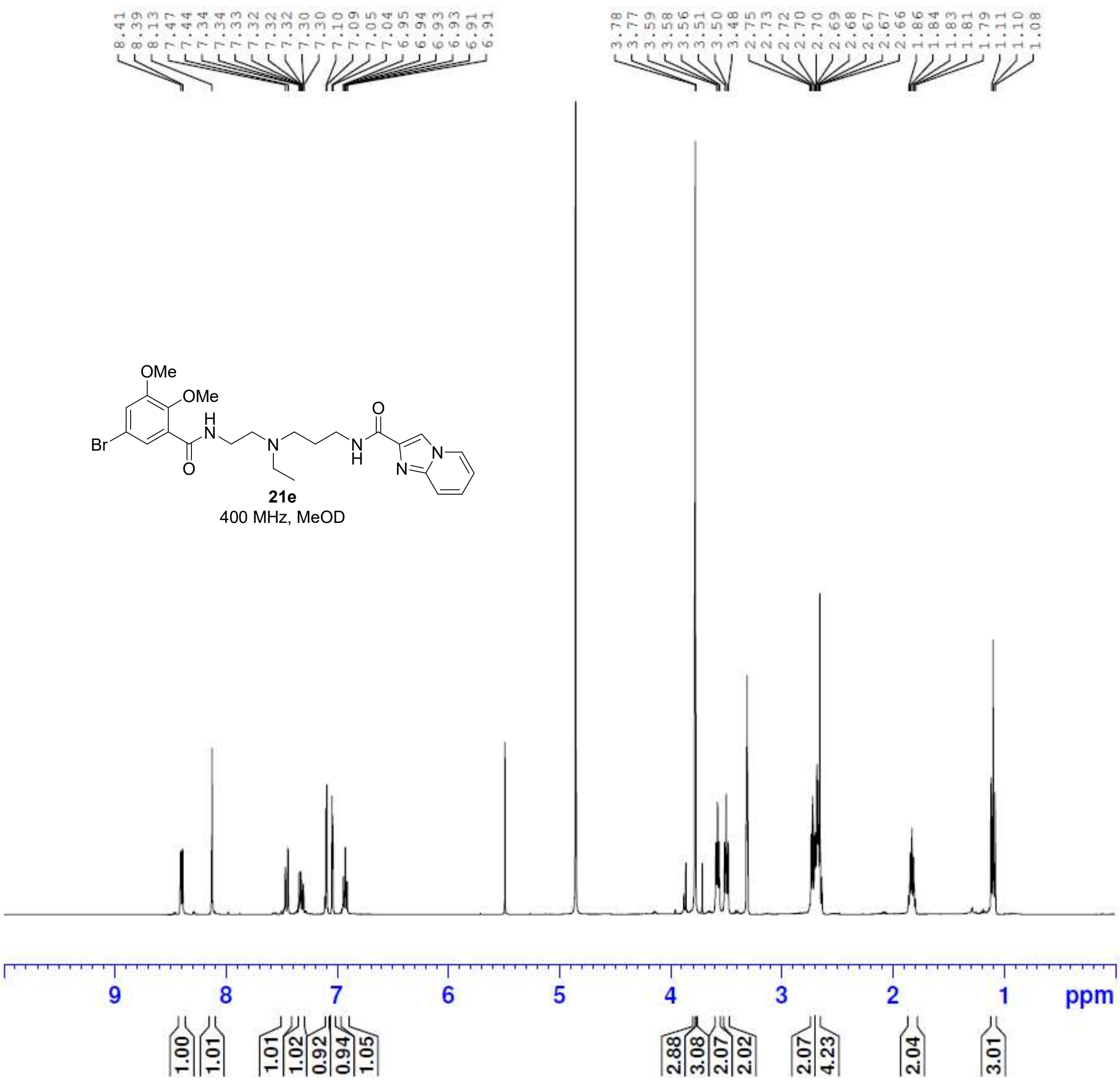
8.41 8.39 8.13 7.47 7.44 7.34 7.34 7.33 7.32 7.32 7.30 7.30 7.10 7.09 7.05 7.04 6.95 6.94 6.93 6.93 6.91 6.91
3.78 3.77 3.59 3.58 3.56 3.51 3.50 3.48 2.75 2.73 2.72 2.70 2.70 2.69 2.68 2.67 2.67 2.66 1.86 1.84 1.83 1.81 1.79 1.11 1.10 1.08
OMe
OMe
Br
H
N
N
O
O
N
H
N
N
21e
400 MHz, MeOD
9 8 7 6 5 4 3 2 1 ppm
1.00 1.01 1.01 1.02 0.92 0.94 1.05 2.88 3.08 2.07 2.02 2.07 4.23 2.04 3.01

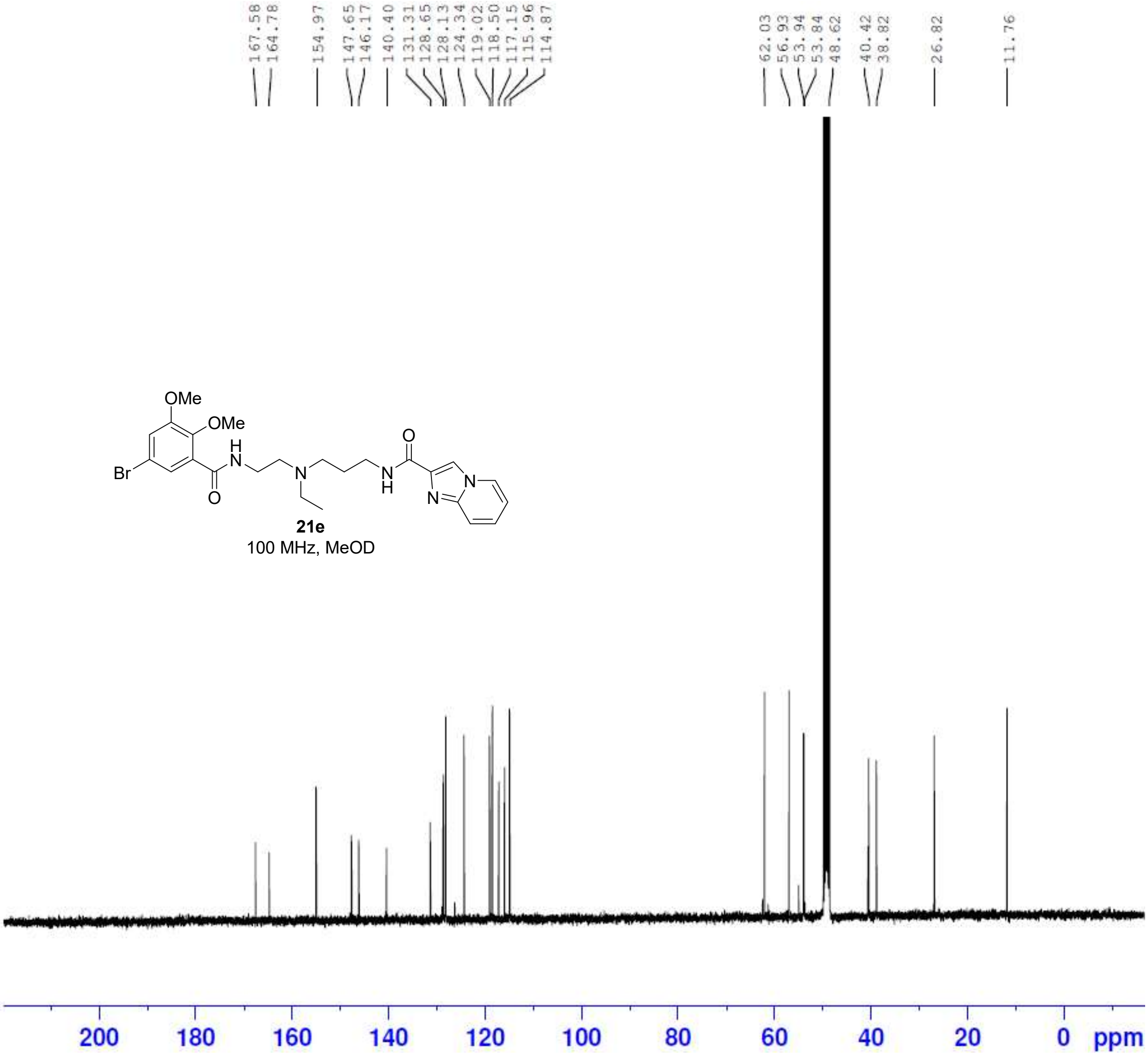
167.58
164.78
154.97
147.65
146.17
140.40
131.31
128.65
128.13
124.34
119.02
118.50
117.15
115.96
114.87
62.03
56.93
53.94
53.84
48.62
40.42
38.82
26.82
11.76
OMe
OMe
Br
H
N
N
O
N
H
O
N
N
21e
100 MHz, MeOD
200
180
160
140
120
100
80
60
40
20
0
ppm

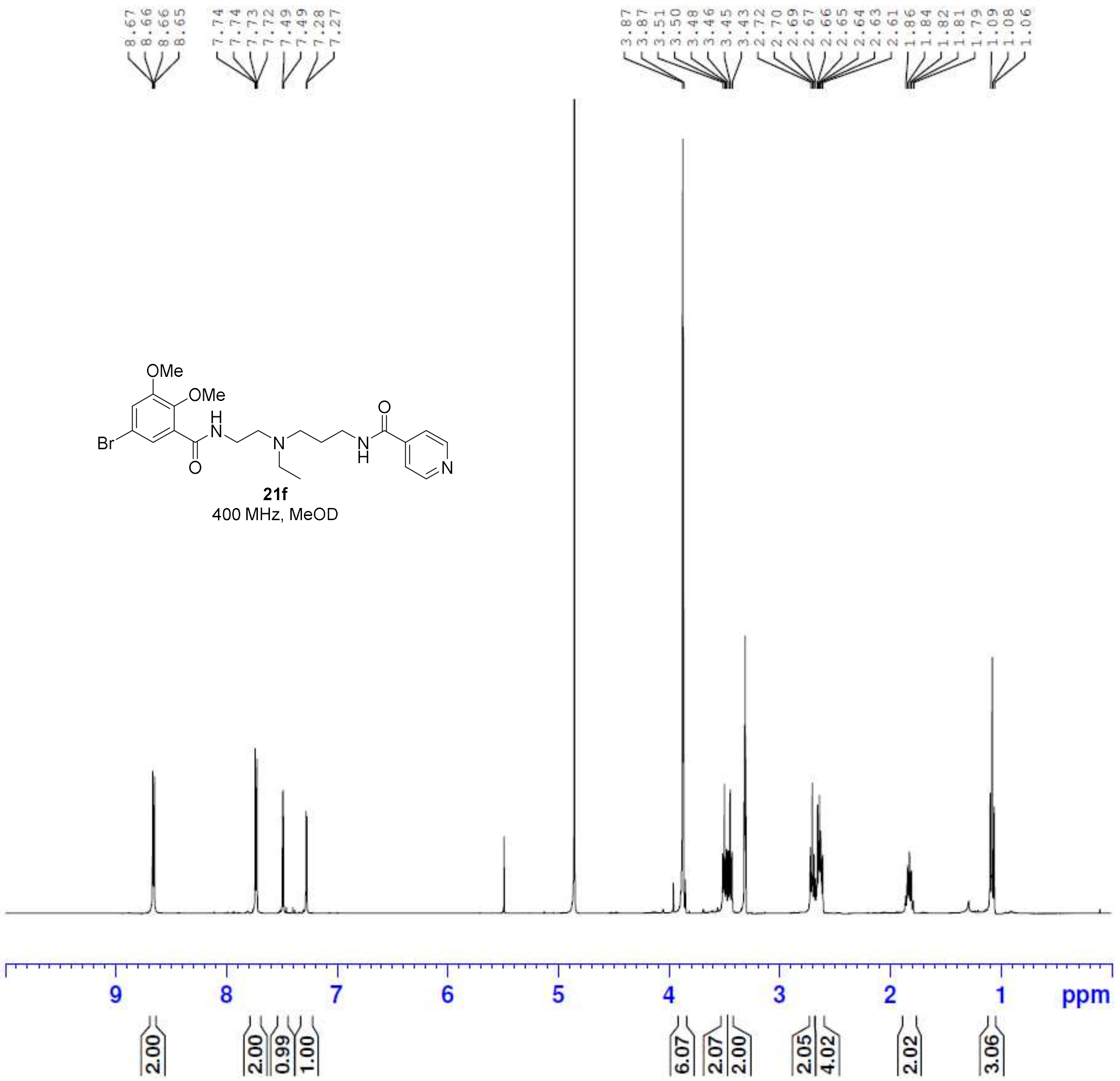

21f
400 MHz, MeOD

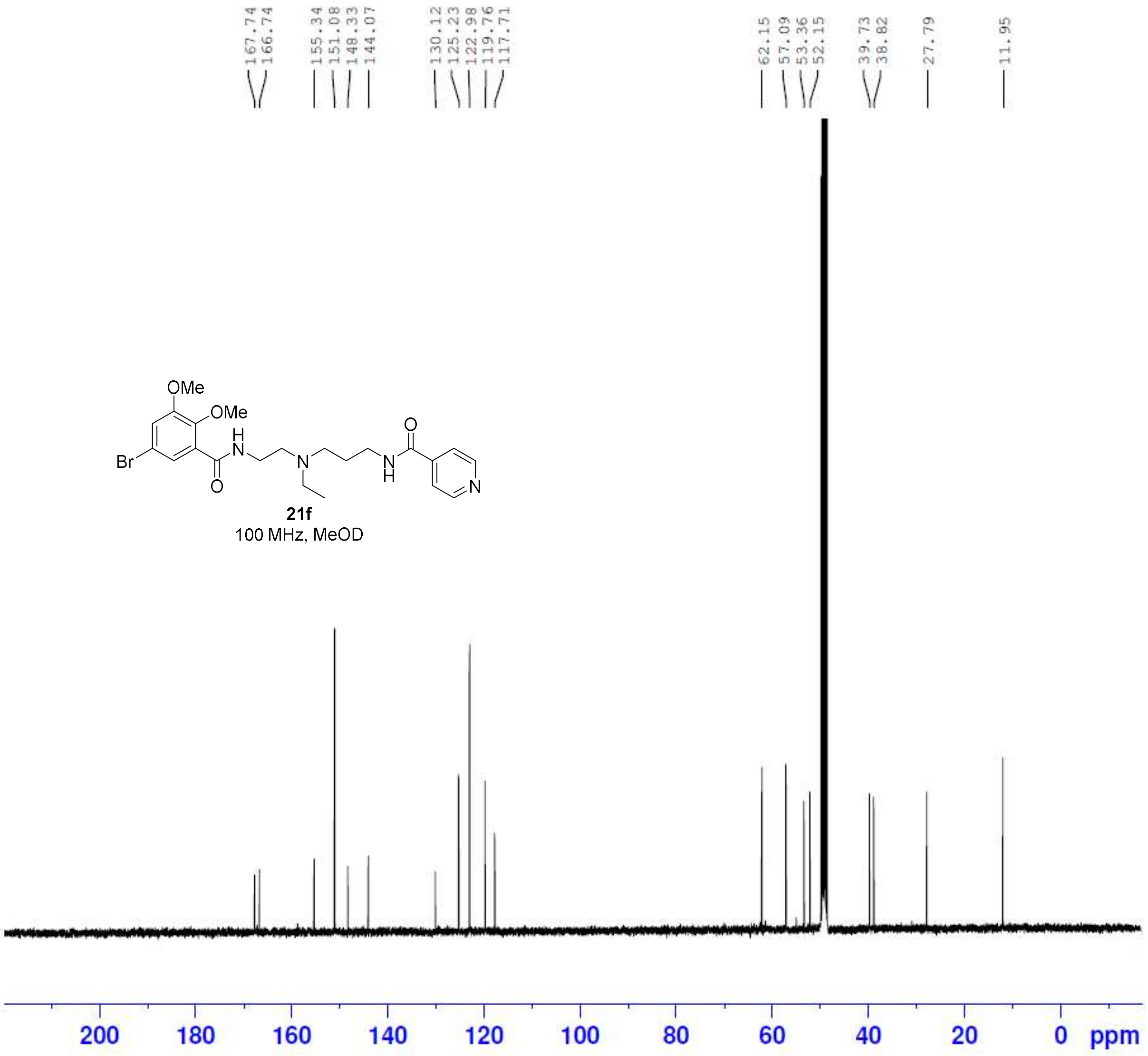
167.74
166.74
155.34
151.08
148.33
144.07
130.12
125.23
122.98
119.76
117.71
62.15
57.09
53.36
52.15
39.73
38.82
27.79
11.95
OMe
OMe
Br
H
N
N
O
O
N
H
N
21f
100 MHz, MeOD
200
180
160
140
120
100
80
60
40
20
0
ppm

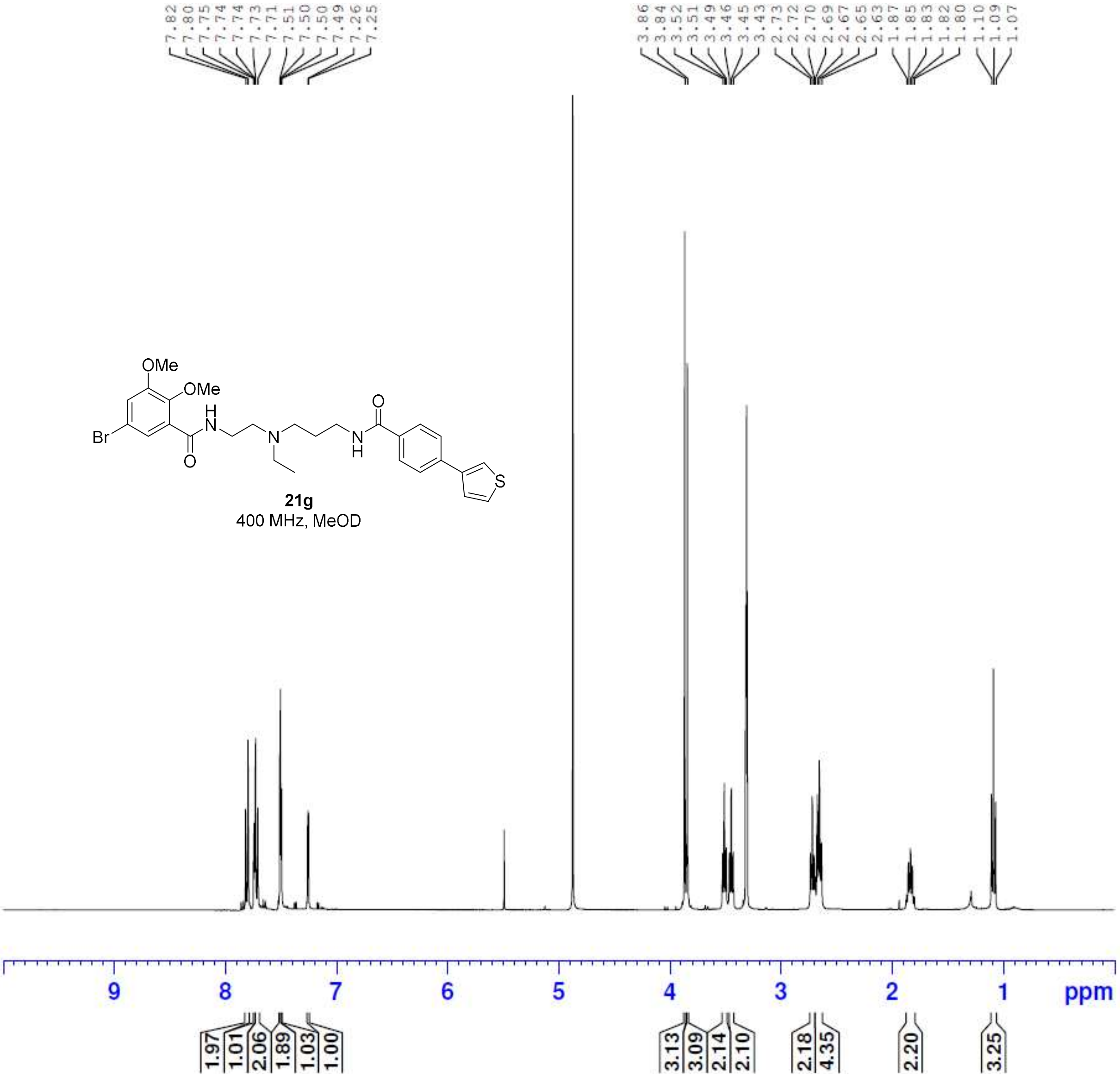
7.82 7.80 7.75 7.74 7.74 7.73 7.71 7.51 7.50 7.50 7.49 7.26 7.25
3.86 3.84 3.52 3.51 3.49 3.46 3.45 3.43 2.73 2.72 2.70 2.69 2.67 2.65 2.63 1.87 1.85 1.83 1.82 1.80 1.10 1.09 1.07
OMe
OMe
Br
H
N
O
N
O
N
H
S
21g
400 MHz, MeOD
9 8 7 6 5 4 3 2 1 ppm
1.97 1.01 2.06 1.89 1.03 1.00
3.13 3.09 2.14 2.10
2.18 4.35
2.20
3.25

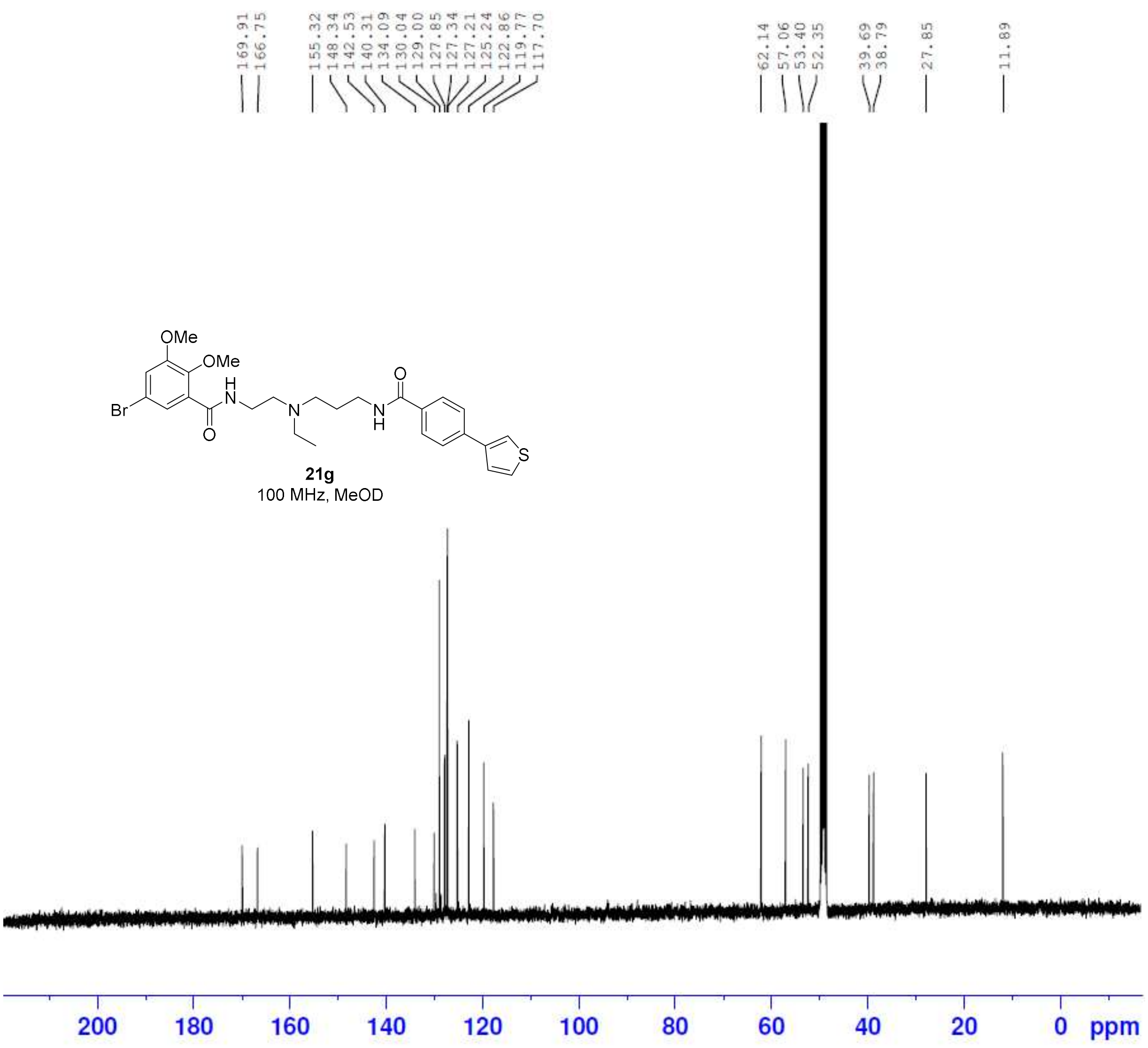
169.91
166.75
155.32
148.34
142.53
140.31
134.09
130.04
129.00
127.85
127.34
127.21
125.24
122.86
119.77
117.70
62.14
57.06
53.40
52.35
39.69
38.79
27.85
11.89
OMe
OMe
Br
H
N
N
O
O
N
H
S
21g
100 MHz, MeOD
200
180
160
140
120
100
80
60
40
20
0
ppm

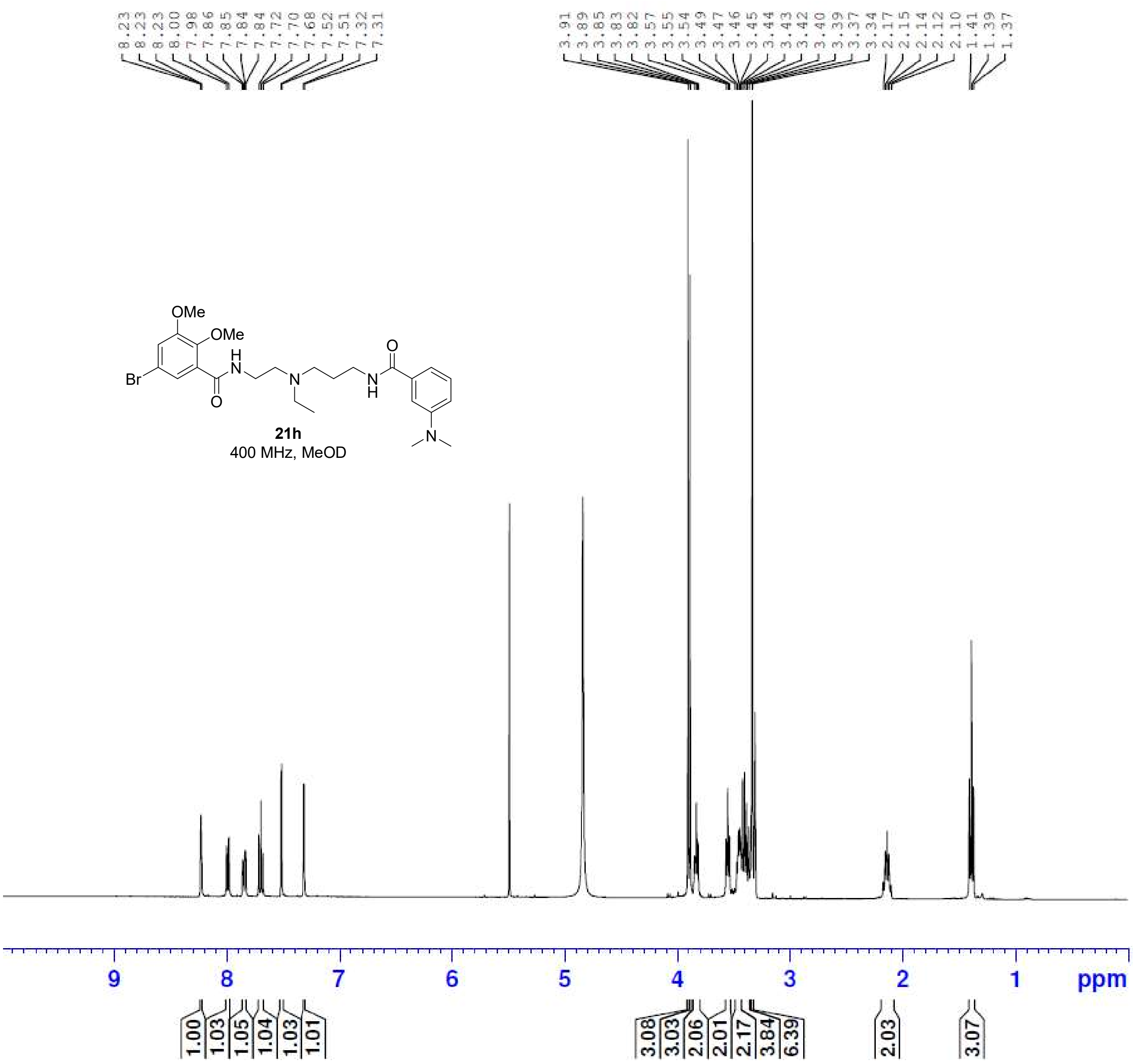
8.23
8.23
8.23
8.00
7.98
7.86
7.85
7.84
7.84
7.72
7.70
7.68
7.52
7.51
7.32
7.31
3.91
3.89
3.85
3.83
3.82
3.57
3.55
3.54
3.49
3.47
3.46
3.45
3.44
3.43
3.42
3.40
3.39
3.37
3.34
2.17
2.15
2.14
2.12
2.10
1.41
1.39
1.37
OMe
OMe
H
N
Br
O
N
O
N
H
N
21h
400 MHz, MeOD
9
8
7
6
5
4
3
2
1
ppm
1.00
1.03
1.05
1.04
1.03
1.01
3.08
3.03
2.06
2.01
2.17
3.84
6.39
2.03
3.07

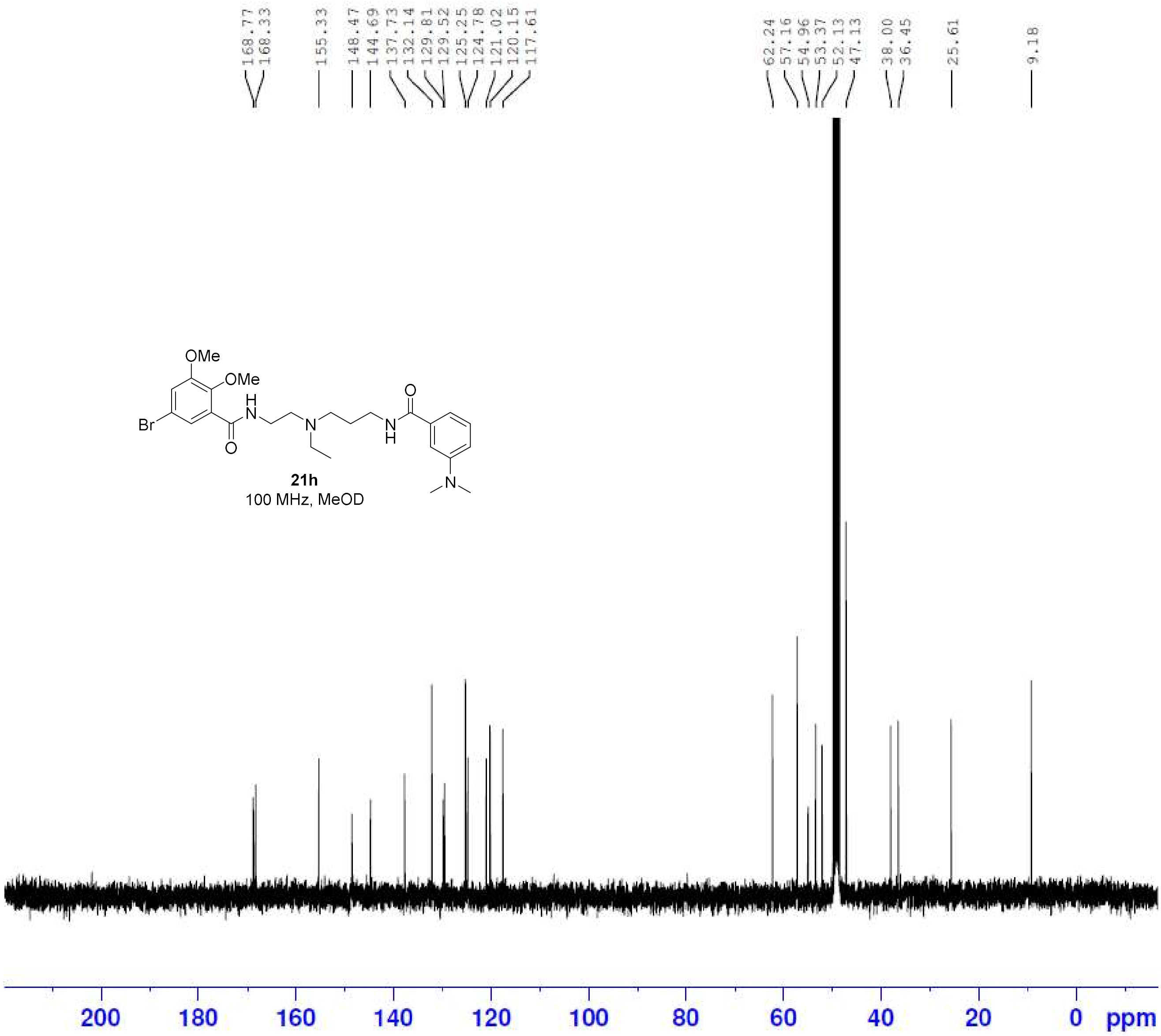
168.77
168.33
155.33
148.47
144.69
137.73
132.14
129.81
129.52
125.25
124.78
121.02
120.15
117.61
62.24
57.16
54.96
53.37
52.13
47.13
38.00
36.45
25.61
9.18
OMe
OMe
Br
H
N
N
N
H
O
O
N
21h
100 MHz, MeOD
200
180
160
140
120
100
80
60
40
20
0
ppm

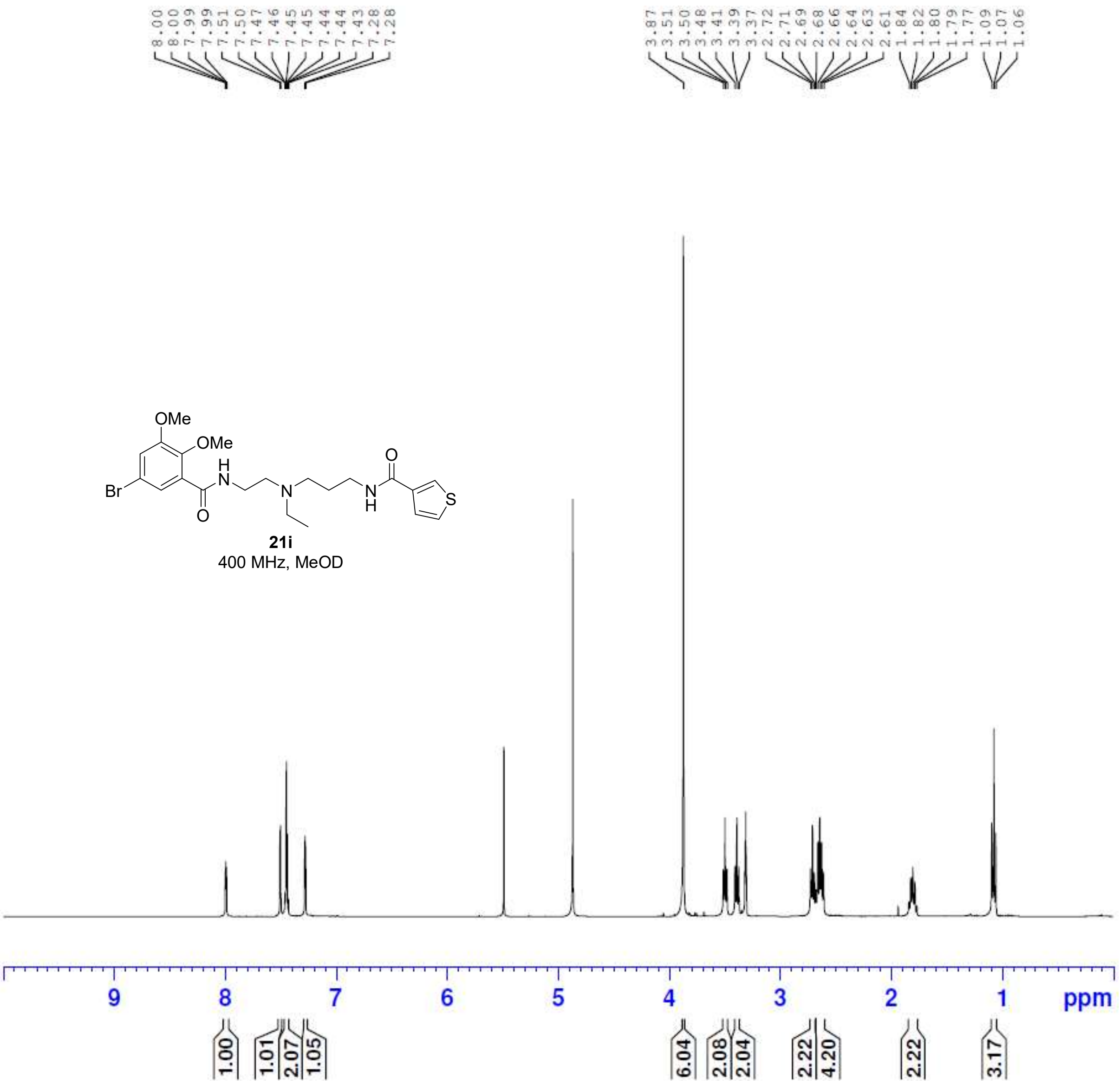
8.00
8.00
7.99
7.99
7.51
7.50
7.47
7.46
7.45
7.45
7.44
7.44
7.43
7.43
7.28
7.28
3.87
3.51
3.50
3.48
3.41
3.39
3.37
2.72
2.71
2.69
2.68
2.66
2.64
2.63
2.61
1.84
1.82
1.80
1.79
1.77
1.09
1.07
1.06
OMe
OMe
Br
H
N
N
N
H
O
O
S
21i
400 MHz, MeOD
9
8
7
6
5
4
3
2
1
ppm
1.00
1.01
2.07
1.05
6.04
2.08
2.04
2.22
4.20
2.22
3.17

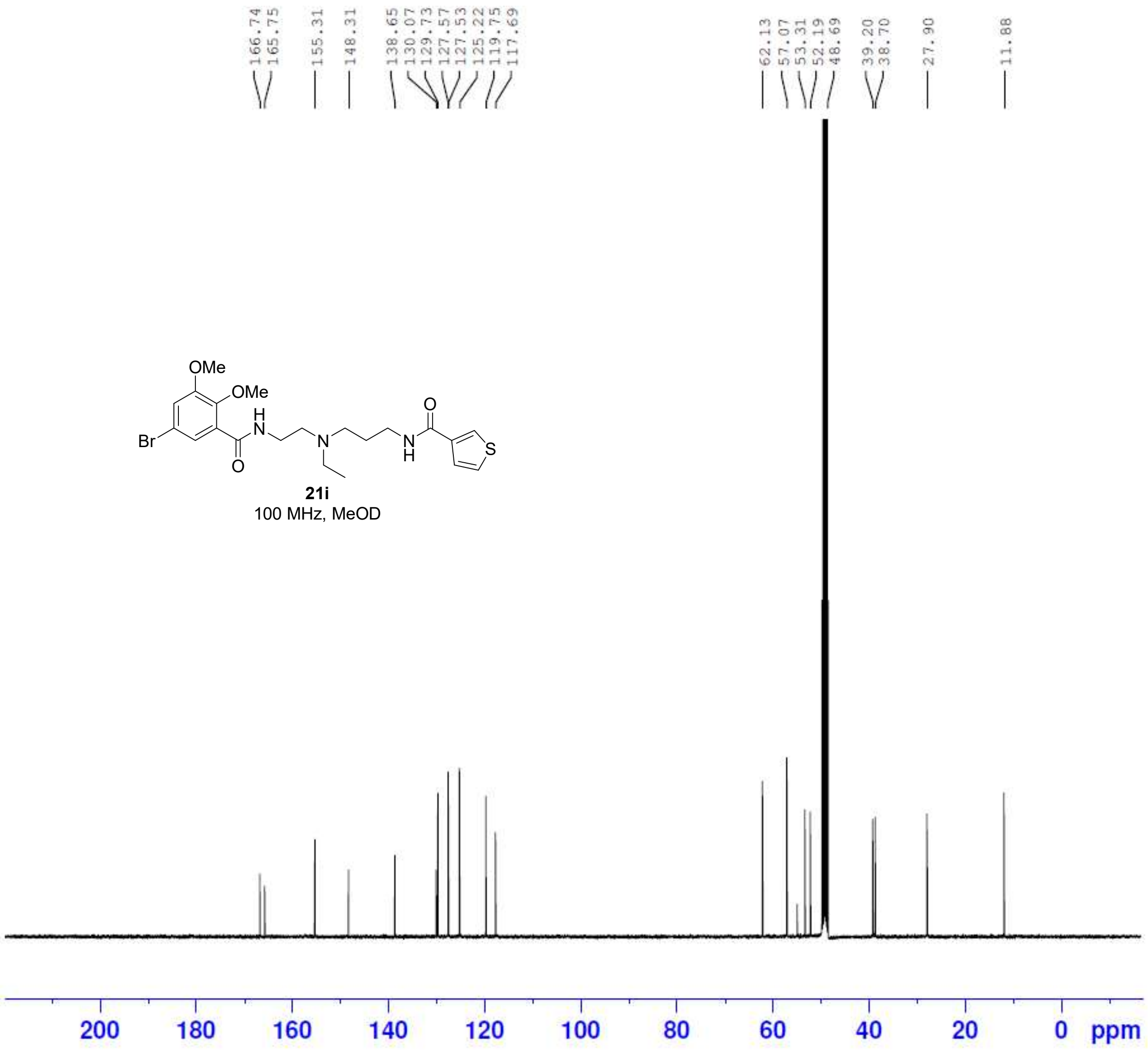

21i

100 MHz, MeOD

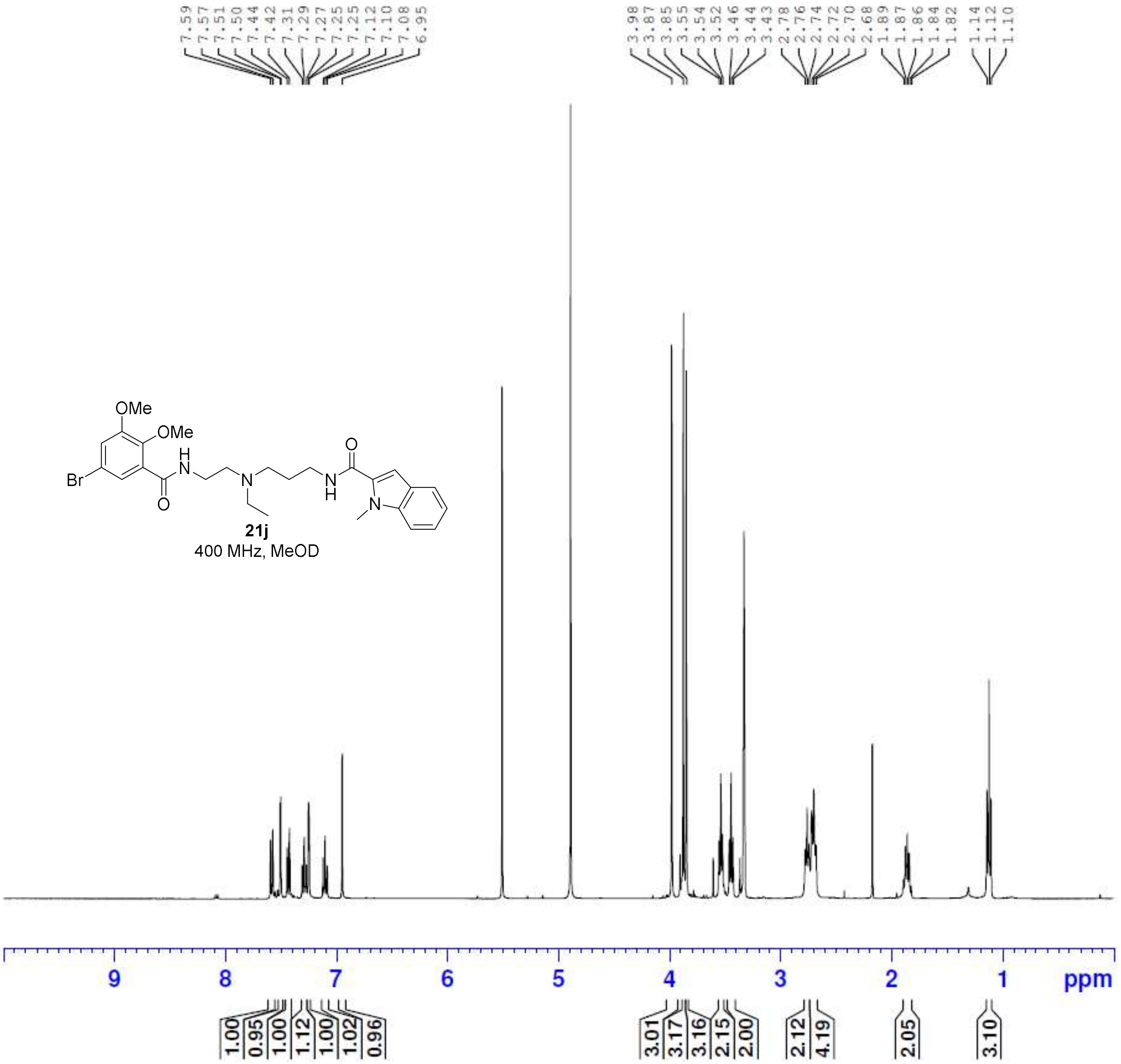
7.59 7.57 7.51 7.50 7.44 7.42 7.31 7.29 7.27 7.25 7.25 7.12 7.10 7.08 6.95
3.98 3.87 3.85 3.55 3.54 3.52 3.46 3.44 3.43 2.78 2.76 2.74 2.72 2.70 2.68 1.89 1.87 1.86 1.84 1.82
1.14 1.12 1.10
OMe
OMe
Br
H
N
N
O
N
H
O
N
21j
400 MHz, MeOD
9 8 7 6 5 4 3 2 1 ppm
1.00 0.95 1.00 1.12 1.00 1.02 0.96
3.01 3.17 3.16 2.15 2.00
2.12 4.19
2.05
3.10

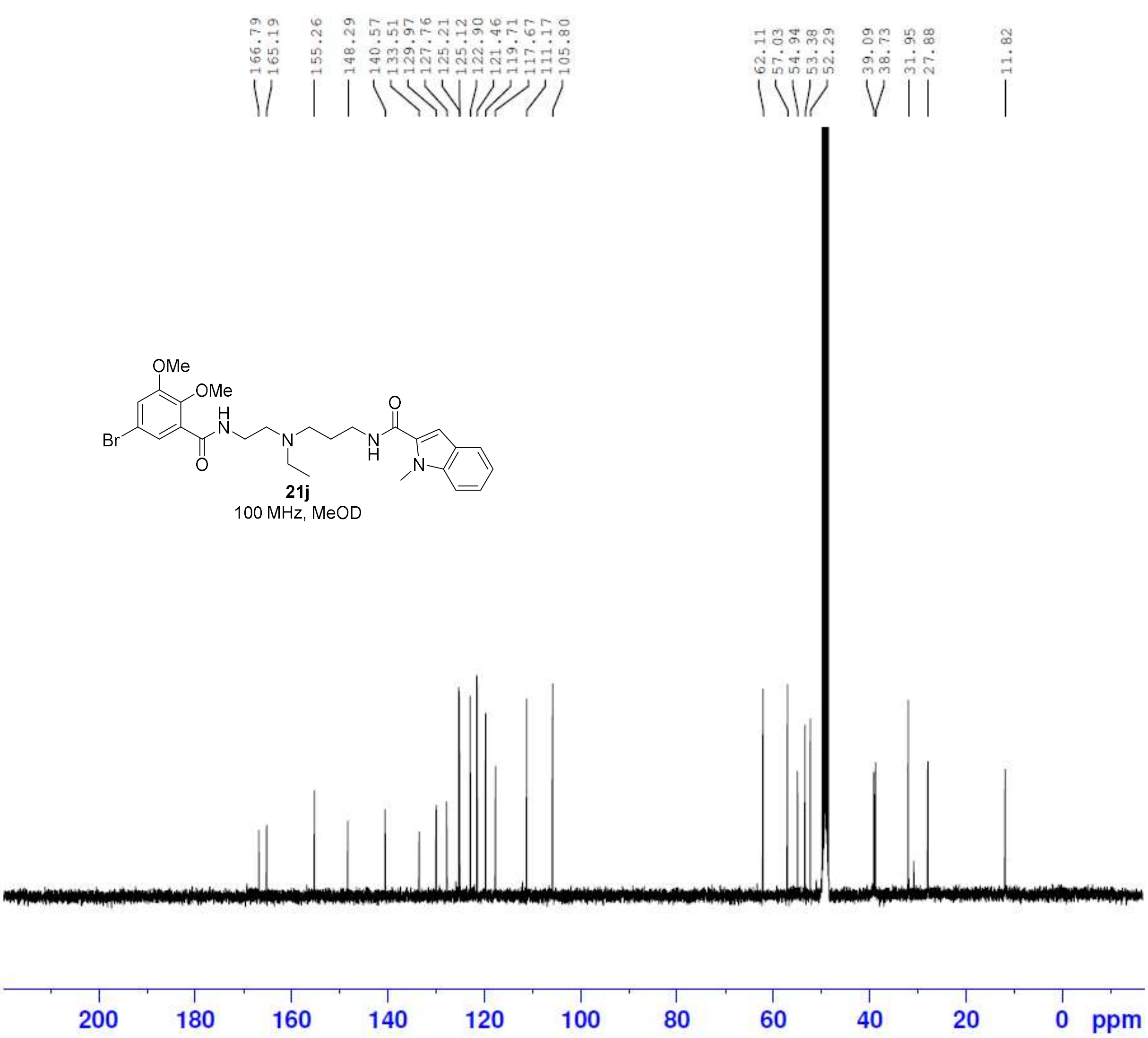

21j
100 MHz, MeOD
